# Reversible Control of Gene Expression by Guest-Modified Adenosines in a Cell-Free System via Host–Guest Interaction

**DOI:** 10.1021/jacs.4c04262

**Published:** 2024-06-28

**Authors:** Hidenori Okamura, Takeyuki Yao, Fumi Nagatsugi

**Affiliations:** † Institute of Multidisciplinary Research for Advanced Materials, Tohoku University, 2-1-1 Katahira, Aoba-ku, Sendai 980-8577, Japan; ‡ Department of Chemistry, Graduate School of Science, Tohoku University, Miyagi 980-8578, Japan

## Abstract

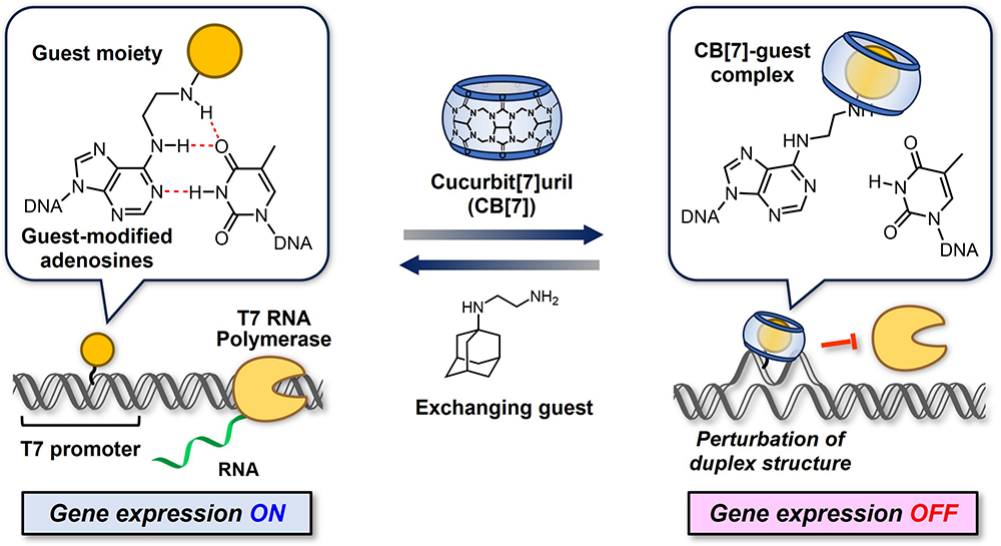

Gene expression technology has become an indispensable tool for elucidating biological processes and developing biotechnology. Cell-free gene expression (CFE) systems offer a fundamental platform for gene expression-based technology, in which the reversible and programmable control of transcription can expand its use in synthetic biology and medicine. This study shows that CFE can be controlled via the host–guest interaction of cucurbit[7]uril (CB[7]) with *N*^6^-guest-modified adenosines. These adenosine derivatives were conveniently incorporated into the DNA strand using a post-synthetic approach and formed a selective and stable base pair with complementary thymidine in DNA. Meanwhile, alternate addition of CB[7] and the exchanging guest molecule induced the reversible formation of a duplex structure through the formation and dissociation of a bulky complex on DNA. The kinetics of the reversibility was fine-tuned by changing the size of the modified guest moieties. When incorporated into a specific region of the T7 promoter sequence, the guest-modified adenosines enabled tight and reversible control of in vitro transcription and protein expression in the CFE system. This study marks the first utility of the host–guest interaction for gene expression control in the CFE system, opening new avenues for developing DNA-based technology, particularly for precise gene therapy and DNA nanotechnology.

## Introduction

Gene expression technology is indispensable for the elucidation of biological processes and the development of biotechnology, including gene therapy. Cell-free gene expression (CFE) is a minimal yet versatile platform for studying gene expression in life science.^1,2^ The CFE system allows the production of functional RNA and protein from natural or synthetic DNA genes under conditions that are incompatible with living cells without the limitations of molecular transport. Thus, the composition and concentration of the components can be customized in a scalable manner to determine the optimal conditions for specific applications. Owing to these advantages, CFE systems have been used to investigate biological processes,^3,4^ biomolecular manufacturing,^5−7^ synthetic biology,^8−10^ the construction of synthetic cells for biosensing,^11,12^ intercellular communication,^13^ and therapeutic applications.^14,15^

To expand CFE applications, CFE systems should be controllable and programmable. A potential approach is the utilization of stimulus-responsive chemical entities to modulate the structures and protein-interactive mode of DNA or RNA.^16−18^ Thus, photoresponsive nucleic acids have been exclusively implemented for gene expression control in CFE systems, as exemplified by the photocontrol of transcription activity using azobenzene derivatives^19,20^ and bulky photocleavable moieties.^21−23^ Although these methods allow remote and spatiotemporal control of gene expression, light can induce a potential dysfunction of the biological components of CFE systems.^20,24^ Furthermore, photochemistry-based methods present intrinsic limitations for in vivo applications because of the low permeability of light.

Ligand-based approaches are another pathway for controlling CFE systems. The ligand-driven control of nucleic acids affords potential biocompatibility by preventing the dysfunction of CFE components. In addition, the ligands can be designed to be delivered deep in the tissue and activated at a specific site or an environment. Moreover, the levels and rates of gene expression can be programmed by fine-tuning the binding property between the ligands and nucleic acids. Riboswitches represent a major class of ligand-based solutions for translation control in CFE systems.^25,26^ For example, activation of synthetic cell functions was demonstrated through the development of histamine-responsive riboswitches.^27^ Alternatively, transcription suppression has been demonstrated using DNA binders^28,29^ or by inserting DNA aptamers into DNA.^30^ Precise OFF–ON control at the transcription level is advantageous because it enables stimulus-responsive signal amplification in an all-or-nothing manner. However, most of the reported approaches are limited to irreversible transcription suppression with a leaky off-state, which hampers their application in CFE-based technologies.

Thus, we aimed at creating a ligand-responsive molecular system that allows robust and reversible control of transcription in a cell-free environment. T7 RNA polymerase is widely used in CFE systems, and its transcription efficiency is dependent on the conformation of the promoter region.^31^ Therefore, the reversible control of duplex formation in the promoter region would directly lead to transcriptional switching. To directly achieve this, chemically modified nucleosides should be incorporated to dynamically change the local structure of the DNA duplex in response to specific ligand binding. However, to be applicable to reversible gene expression control in CFE systems, such modified nucleosides must exhibit the following: (1) natural-like base-pairing properties in the absence of ligands to not disturb DNA hybridization and RNA polymerase recognition, (2) sufficient affinity for a specific ligand, and (3) induction of dynamic structural change in response to the ligand binding.

In addition, the ligands must be compatible with CFE systems. To create ligand-responsive nucleosides that satisfy the aforementioned criteria, we considered harnessing the host–guest chemistry of cucurbit[7]uril (CB[7]).^32,33^ CB[7] exhibits a hollow structure that enables the formation of bulky inclusion complexes with hydrophobic guests. Compared with the well-known host–guest interactions of cyclodextrins whose equilibrium association constants (*K*_a_) lie in the millimolar to micromolar range,^34,35^ CB[7] shows considerably higher affinity to certain guest molecules. In optimal cases, *K*_a_ of CB[7] and guest molecules exceeds 10^15^ M^–1^.^36−38^ CB[7] can be displaced from the guest molecule through a guest exchange reaction by the addition of higher-affinity guest molecules. Although its compatibility with CFE systems remains undetermined, the host–guest chemistry of CB[7] can be tolerated under biological conditions.^39−42^ Owing to the unique characteristics of CB[7], we postulate that the reversible duplex formation can be achieved via host–guest interaction by appropriately incorporating a guest moiety onto DNA.

**Figure 1:**
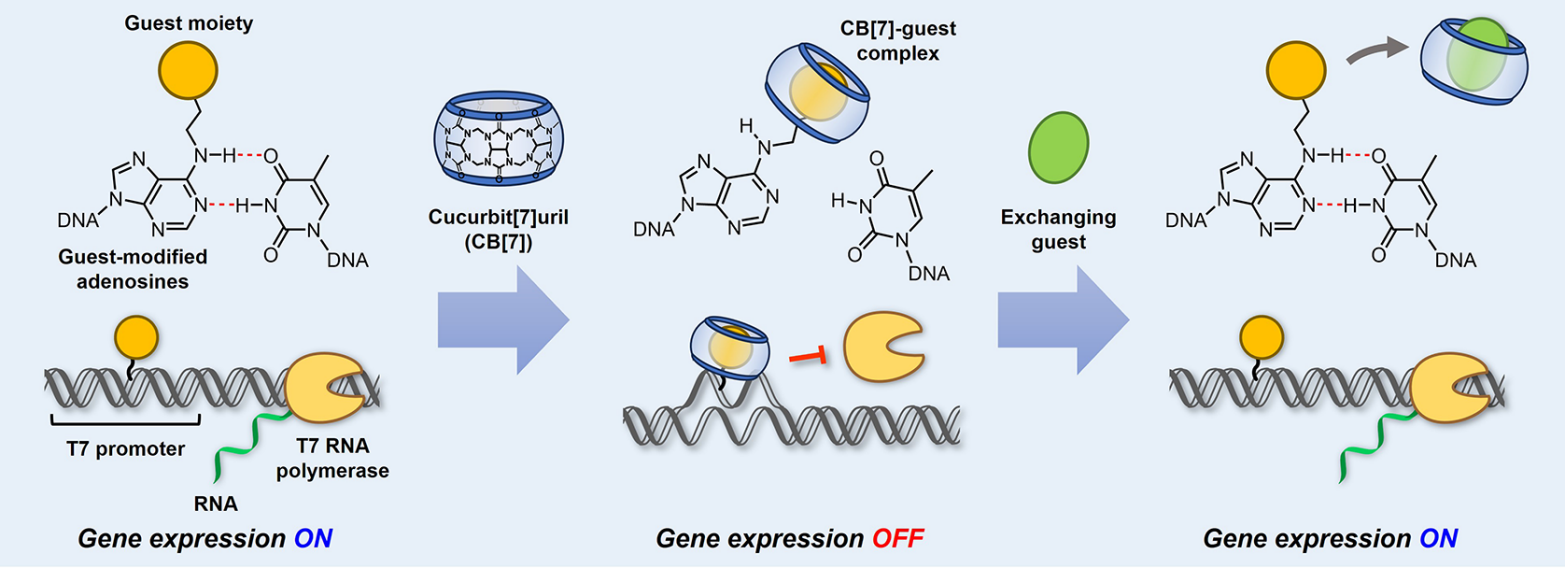
Reversible gene expression system driven by guest-modified adenosines via the host–guest interaction of CB[7]. The guest-modified adenosines formed stable base pairs with thymidine in the DNA duplex, allowing gene expression driven by the T7 promoter. Upon adding CB[7], the formation of a bulky CB[7]-guest complex sterically destabilized the DNA duplex, thereby inhibiting the initiation of the transcription by T7 polymerase. The addition of an exchanging guest facilitated the decomplexation of CB[7] to reform the DNA duplex, enabling the iterative OFF–ON control of gene expression.

Herein, we describe the development of guest-modified adenosine derivatives bearing a guest moiety at the *N*^6^-position for the reversible control of gene expression in CFE systems (Figure 1). In the DNA duplex, these nucleosides were expected to form a stable base pair with thymidine (T). In addition, the complexation of the guest moiety with CB[7] was anticipated to decrease the local stability of the duplex by sterically disturbing the base pair formation with T and those of the adjacent bases. Furthermore, the addition of exchanging guest molecules with higher affinity was considered to facilitate the decomplexation of CB[7] from the adenosine derivative and recover the original duplex. We expected that by incorporating such guest-modified adenosines into the T7 promoter sequence, reversible control of CFE could be achieved via host–guest interaction.

## Results and Discussion

### Design and Synthesis of Guest-Modified Adenosines

**Figure 2:**
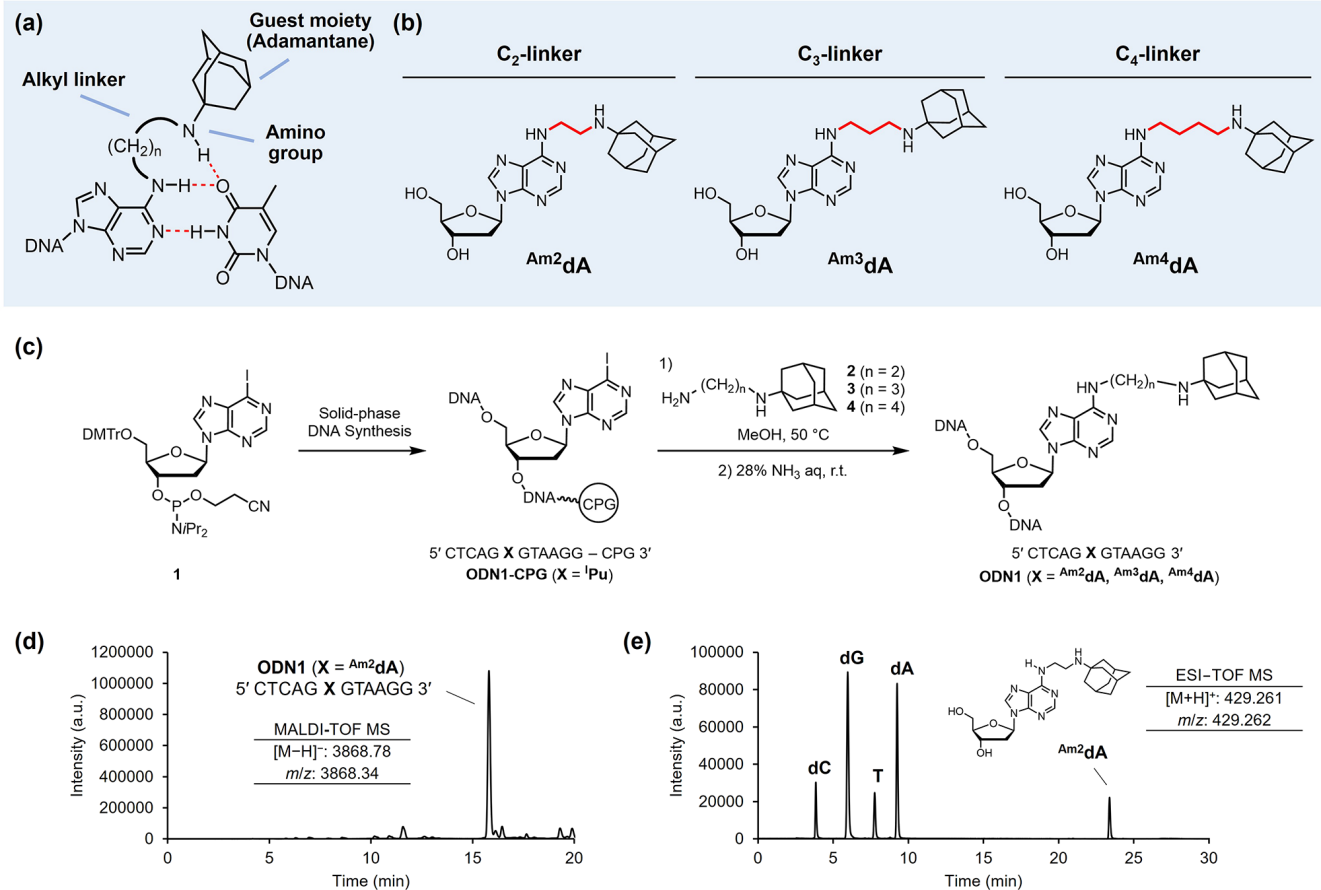
(a) Guest-modified adenosines. Here, 1-aminoadamantane was attached to the *N*^6^-position of adenosine as a guest moiety through an alkyl linker. The amino group endowed the base pair with additional stability through hydrogen bonding. (b) Structures of adamantane-modified adenosines (^**Am2**^**dA**, ^**Am3**^**dA**, and ^**Am4**^**dA**) with different linker lengths. (c) Synthesis of ODN incorporating ^**Am2**^**dA**, ^**Am3**^**dA**, and ^**Am4**^**dA** via post-synthetic approach. (d) Crude RP-HPLC chart and MALDI-TOF MS data of **ODN1** (**X** = ^**Am2**^**dA**). (e) RP-HPLC chart of digested **ODN1** (**X** = ^**Am2**^**dA**). The formation of the ^**Am2**^**dA** nucleoside was confirmed by ESI-TOF MS measurement.

Figure 2a shows the molecular design of the guest-modified adenosine. The guest moiety was attached to the adenine core at the *N*^6^-position through an alkyl linker. In the primary design, 1-aminoadamantane was selected as the guest moiety because of its considerably high affinity for CB[7] (*K*_d_ = 2.4 × 10^–13^ M).^38^ The secondary amino group was expected to impart additional stability to the base pair through the formation of an additional hydrogen bond with the carbonyl group at the 4-position of thymine in a clamp-like recognition mode. To competitively destabilize the DNA duplex via host–guest interaction, the linker length was considered critical for achieving an effective steric clash with the Watson–Crick interface and neighboring bases. For determining the appropriate linker length, we initially designed three adenosine derivatives, each modified with 1-aminoadamantane through the C_2_-, C_3_-, and C_4_-linkers (^**Am2**^**dA**, ^**Am3**^**dA**, and ^**Am4**^**dA**, respectively; Figure 2b).

The oligodeoxynucleotides (ODNs) incorporating ^**Am2**^**dA**, ^**Am3**^**dA**, and ^**Am4**^**dA** were prepared using a post-synthetic approach in which the modified nucleosides were synthesized from the corresponding convertible nucleosides within an ODN. This method enabled the systematic and convenient preparation of chemically modified ODNs while circumventing the redundant synthesis of the corresponding phosphoramidite building blocks. We hypothesized that 6-iodopurine 2′-deoxyriboside (^**I**^**Pu**) in the solid support-bound ODN would undergo an S_N_Ar reaction with adamantane-tethered alkylamines to afford ODNs with guest-modified adenosines at specific positions.^43,44^ The ^**I**^**Pu** phosphoramidite (**1**) was synthesized and incorporated into 12-mer **ODN1** using an automated DNA synthesizer, as described in our previous report.^45^Scheme S1 describes the preparation of adamantane-tethered alkyl amines **2**–**4**. The CpG-bound **ODN1** (**X** = ^**I**^**Pu**) was reacted with each amine in methanol at 50 °C (Figure 2c). After ammonium hydroxide treatment for deprotection and cleavage from the solid support, the crude ODN was analyzed by RP-HPLC. As exemplified in the case of ^**Am2**^**dA**, the appearance of a major peak indicated the progress of the post-synthetic modification (Figure 2d). The structural integrity and purity of the isolated **ODN1** (**X** = ^**Am2**^**dA**) were confirmed by MALDI-TOF MS and RP-HPLC, respectively (Table S1, Figure S1).

To further ensure the formation of ^**Am2**^**dA** in the DNA, the purified **ODN1** (**X** = ^**Am2**^**dA**) was digested into nucleosides by nucleases. The RP-HPLC of the digest revealed the formation of a nucleoside apart from four canonical nucleosides (Figure 2e), which was confirmed via ESI-TOF MS to be an ^**Am2**^**dA** nucleoside. The **ODN1** incorporating the other guest-modified adenosines was similarly prepared and characterized (Table S1, Figures S1 and S2).

### Base-Pairing Properties of Adamantane-Modified Adenosines

**Figure 3:**
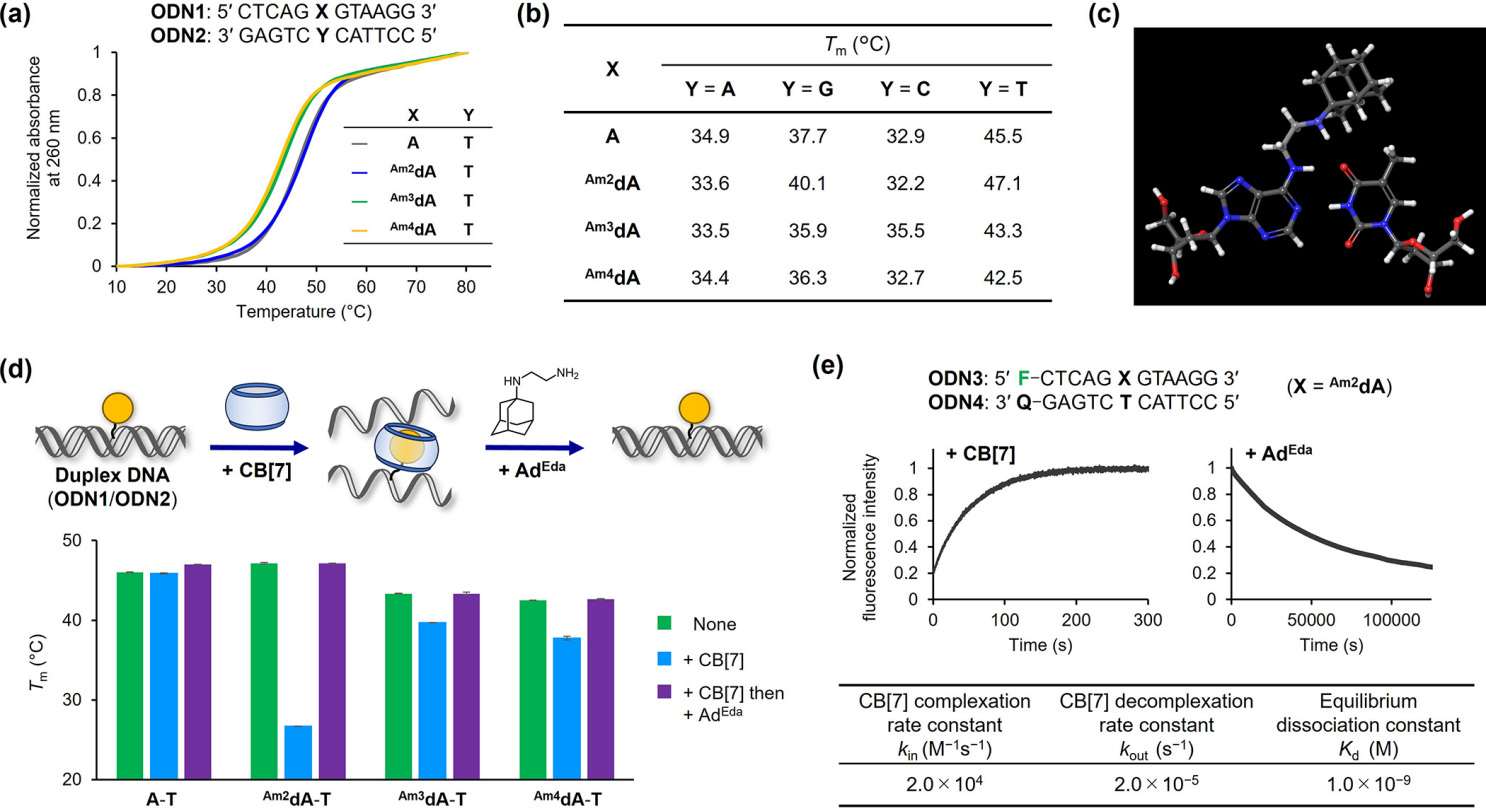
(a) UV melting curves of DNA duplexes (**ODN1**/**ODN2**) incorporating **A**-**T**, ^**Am2**^**dA**-**T**, ^**Am3**^**dA**-**T**, and ^**Am4**^**dA**-**T**. Conditions: DNA duplex (2 μM) in sodium phosphate buffer (10 mM, pH 7.0) and NaCl (150 mM). (b) *T*_m_ of DNA duplexes containing adamantane-modified adenosines. (c) Speculated recognition mode of the ^**Am2**^**dA**-**T** pair. (d) *T*_m_ of DNA duplexes containing the **A**-**T**, ^**Am2**^**dA**-**T**, ^**Am3**^**dA**-**T**, and ^**Am4**^**dA**-**T** pairs after sequential addition of CB[7] and Ad^Eda^. Conditions: DNA duplex (2 μM), CB[7] (4 μM), Ad^Eda^ (10 μM) in sodium phosphate buffer (10 mM, pH 7.0), and NaCl (150 mM). The error bars represent the standard errors (*N* = 3). (e) Time course fluorescence monitoring of the dye-labeled DNA duplex (**F**: FAM, **Q**: Dabcyl) containing the ^**Am2**^**dA**-**T** pair after sequential addition of CB[7] and Ad^Eda^. The kinetic parameters were determined via a nonlinear least-squares regression analysis of the respective curves. Conditions: DNA duplex (100 nM), CB[7] (1 μM), Ad^Eda^ (2 μM) in sodium phosphate buffer (10 mM, pH 7.0), and NaCl (150 mM) at 37 °C. Fluorescence was monitored at λ_ex_ = 495 nm and λ_em_ > 525 nm.

We investigated the base-pairing selectivity and thermal stability of ODNs incorporating the adamantane-modified adenosines by measuring their melting temperature (*T*_m_). Figure 3a shows representative UV melting curves of the DNA duplexes formed between **ODN1** (**X** = **A**, ^**Am2**^**dA**, ^**Am3**^**dA**, and ^**Am4**^**dA**) and the complementary **ODN2** (**Y** = **T**). The *T*_m_ values against four nucleobases (A, G, C, and T) are listed in Figure 3b (see Figure S3 for the UV melting curves). **ODN1** (**X** = **A**) exhibited selective duplex formation with **ODN2** (**X** = **T**) at a *T*_m_ of 45.5 °C. Similarly, ^**Am2**^**dA**, ^**Am3**^**dA**, and ^**Am4**^**dA** exhibited selectivity toward complementary T. Among the three adenosine derivatives, the thermal stability of the ^**Am2**^**dA**-**T** pair (*T*_m_ = 47.1 °C) was comparable to that of the canonical **A**-**T** pair (*T*_m_ = 45.5 °C) and higher than those of ^**Am3**^**dA**-**T** (*T*_m_ = 43.3 °C) and ^**Am4**^**dA**-**T** (*T*_m_ = 42.5 °C). The alkyl substituents on the 6-NH_2_ group are known to cause the intrinsic destabilization of the base-pairing toward T because of its preference for *anti*-conformation,^46,47^ which may have accounted for the lower thermal stability of ^**Am3**^**dA-T** and ^**Am4**^**dA-T** compared with that of the **A**-**T** pair. The stability of the ^**Am2**^**dA**-**T** pair was attributed to the formation of an additional hydrogen bond between the secondary amino and 4-carbonyl groups of thymine, which compensated for the energy penalty caused by the *anti*-to-*syn* isomerization (Figure 3c).

To investigate the potential contribution of the additional hydrogen bond to the thermal stability of the ^**Am2**^**dA**-**T** pair, we synthesized additional adenosine derivatives bearing ethyl (^**Et**^**dA**) and aminoethyl (^**AEt**^**dA**) groups at the *N*^6^-position (Figure S4; Table S1, Figures S1 and S2) and evaluated their base pairing properties. Unlike the ^**Am2**^**dA**-**T** pair, the ^**Et**^**dA**-**T** pair destabilized duplex formation (*T*_m_ = 42.7 °C) presumably due to the conformational penalty accompanying the *syn*-to-*anti* isomerization of the ethyl moiety at the *N*^6^-position. Contrarily, the ^**AEt**^**dA**-**T** pair with the amino group at the linker terminus exhibited stability (*T*_m_ = 45.7 °C) comparable to that of the **A**-**T**. These results support the recognition structure of ^**Am2**^**dA,** as shown in Figure 3c.

Next, we determined if the base-pairing ability of the guest-modified adenosines could be reversibly controlled by the host–guest interaction. Thus, *T*_m_ measurements were conducted with the DNA duplexes (**ODN1**/**ODN2**) containing ^**Am2**^**dA**-**T**, ^**Am3**^**dA**-**T**, ^**Am4**^**dA**-**T**, and **A**-**T** pairs at position **X**–**Y** upon sequential incubation with CB[7] and adamantane ethylenediamine (Ad^Eda^) under isothermal conditions at 37 °C (Figures 3d and S5). Ad^Eda^ was selected as an exchanging guest molecule because of its high affinity for CB[7] (*K*_d_ = 4.2 × 10^–14^ M).^38^

When the nonmodified DNA containing an **A**-**T** pair was treated with CB[7], followed by Ad^Eda^, no significant change in the *T*_m_ was observed, indicating that the additives did not alter the thermal stability of the DNA duplex. Contrarily, when the DNA containing an ^**Am2**^**dA**-**T** pair was treated with CB[7], the duplex was significantly destabilized (Δ*T*_m_ = 20.4 °C). An isothermal UV titration study revealed an inflection point at [CB7]/[DNA] ≈ 1, indicating a 1:1 interaction between CB[7] and the ^**Am2**^**dA**-modified DNA (Figure S6). Furthermore, the addition of Ad^Eda^ to the CB[7]-treated DNA duplex led to the recovery of the initial *T*_m_ value. These results show that the duplex formation can be controlled by the reversible complexation between CB[7] and the guest moiety modified on the DNA. Similarly, the DNAs bearing ^**Am3**^**dA**-**T** and ^**Am4**^**dA**-**T** pairs exhibited duplex destabilization and rehybridization upon sequential addition of CB[7] and Ad^Eda^. However, the destabilization effect observed after the addition of CB[7] (^**Am3**^**dA**-**T** and ^**Am4**^**dA**-**T**, Δ*T*_m_ = 3.6 and 4.7 °C, respectively) was smaller than that of ^**Am2**^**dA**-**T** (Δ*T*_m_ = 20.4 °C). It was speculated that although ^**Am3**^**dA** and ^**Am4**^**dA** formed a host–guest complex with CB[7], the C_3_- and C_4_-linkers were too long to induce an effective steric clash in the duplex.

Focusing on ^**Am2**^**dA**, which exhibited the highest transition in *T*_m_ owing to the host–guest interaction, we elucidated the kinetics of the complexation and decomplexation between CB[7] and ^**Am2**^**dA** in the DNA duplex. We prepared a duplex comprising FAM-labeled **ODN3** containing ^**Am2**^**dA** and Dabcyl-labeled complementary **ODN4** (Table S1, Figures S1 and S2) for monitoring the fluorescence changes after sequential treatment with CB[7] and Ad^Eda^. Figure 3e shows that the addition of CB[7] increased the FAM-derived fluorescence intensity, indicating the dissociation of the duplex. The enhanced initial slope of the signals with an increase in the CB[7] concentration suggested that the dissociation of the duplex was a bimolecular process driven by the complexation of ^**Am2**^**dA** and CB[7] (Figure S7). Assuming that the dissociation and association of the DNA duplex proceeded immediately after the host–guest interaction, the time course of each process reflected the complexation and decomplexation rates of ^**Am2**^**dA** and CB[7], respectively. Thus, the duplex dissociation process was analyzed as a pseudo-first-order reaction in the presence of excess CB[7]. The nonlinear least-squares fitting of the curve provided the apparent rate constant of CB[7] complexation as *k*_in_ = 2.0 × 10^4^ M^–1^ s^–1^. The measurements using different concentrations of CB[7] and DNA provided similar *k*_in_ values, thereby validating the approximation (Figures S7 and S8).

Next, the kinetics of the decomplexation reaction were investigated. When Ad^Eda^ was added to a mixture of CB[7]-complexed **ODN3** (**X** = ^**Am2**^**dA**) and **ODN4**, a time course quenching of the fluorescence was observed (Figure 3e), suggesting the rehybridization of the duplex after the guest exchange reaction. An increase in the concentration of Ad^Eda^ or the other guest molecules did not significantly affect the decomplexation rate of CB[7] from ^**Am2**^**dA** (Figure S9). This implied that the decomplexation proceeded via an “S_N_1-type” mechanism (i.e., the guest exchange reaction proceeded through the spontaneous exclusion of the guest moiety from CB[7], followed by substitution with the higher-affinity guest molecules).^48^ Thus, the reaction was analyzed as a first-order kinetic path. The apparent decomplexation rate constant of *k*_out_ = 2.0 × 10^–5^ s^–1^ was obtained via a nonlinear least-squares fit calculation. Using the *k*_in_ and *k*_out_ values, the apparent equilibrium dissociation constant (*K*_d_ = *k*_out_/*k*_in_) was calculated as *K*_d_ = 1.0 × 10^–9^ M; this *K*_d_ value was larger than that of the free 1-aminoadamantane binding to CB[7] (*K*_d_ = 2.4 × 10^–13^ M),^38^ presumably due to bulky CB[7] encountering steric hindrance when binding to the guest moiety embedded in the major groove of the DNA duplex. Nevertheless, *K*_d_ provides a general basis for typical biological applications with working concentrations within a nanomolar range.

### Structural Refinement to Tune the Kinetics of the Reversible Duplex Formation

**Figure 4:**
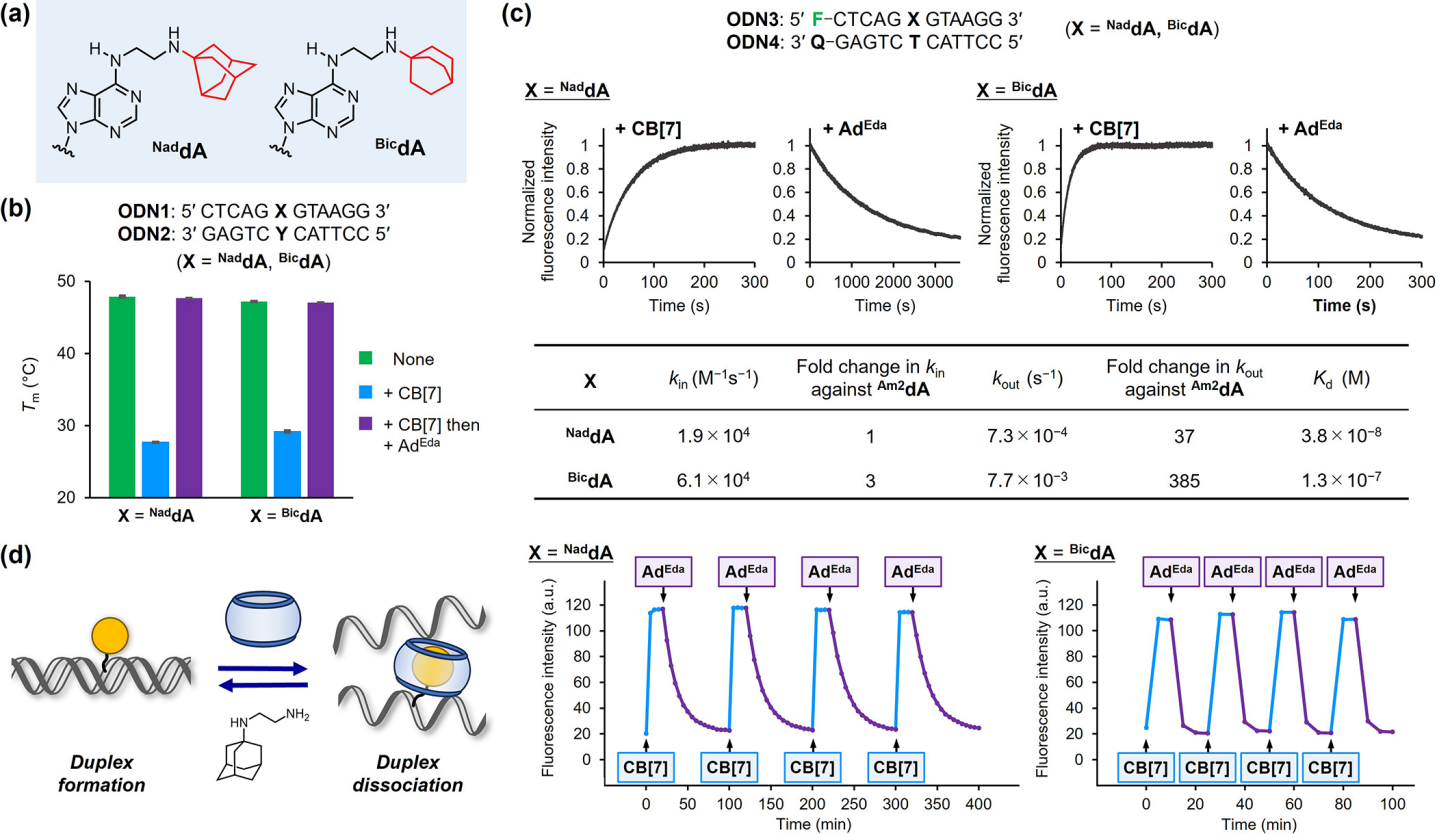
(a) ^**Nad**^**dA** and ^**Bic**^**dA** bearing noradamantane and bicyclo[2.2.2]octane as guest moieties. (b) *T*_m_ of DNA duplexes containing the ^**Nad**^**dA**-**T** and ^**Bic**^**dA**-**T** pairs after the sequential addition of CB[7] and Ad^Eda^. Conditions: DNA duplex (2 μM), CB[7] (4 μM), Ad^Eda^ (10 μM) in sodium phosphate buffer (10 mM, pH 7.0), and NaCl (150 mM). The error bars represent the standard errors (*N* = 3). (c) Time course fluorescence monitoring of the dye-labeled DNA duplex (**F**: FAM, **Q**: Dabcyl) containing ^**Nad**^**dA**-**T** and ^**Bic**^**dA**-**T** pairs after sequential addition of CB[7] and Ad^Eda^. The kinetic parameters were determined through a nonlinear least-squares regression analysis of the respective curves. Conditions: DNA duplex (100 nM), CB[7] (1 μM), Ad^Eda^ (2 μM) in sodium phosphate buffer (10 mM, pH 7.0), and NaCl (150 mM) at 37 °C. The fluorescence was monitored at λ_ex_ = 495 nm and λ_em_ > 525 nm. (d) Iterative switching of duplex formation by DNA containing ^**Nad**^**dA**-**T** and ^**Bic**^**dA**-**T** pairs. CB[7] (0.5, 2, 6, and 15 μM) and Ad^Eda^ (1, 4, 10, and 25 μM) were alternately added while monitoring the fluorescence. Incubation interval: ^**Nad**^**dA**, 20 min of CB[7], followed by 80 min for Ad^Eda^; ^**Bic**^**dA**, 10 min of CB[7], followed by 15 min for Ad^Eda^. Conditions: DNA duplex (100 nM), CB[7] (1 μM), Ad^Eda^ (2 μM) in sodium phosphate buffer (10 mM, pH 7.0), and NaCl (150 mM) at 37 °C. Fluorescence was monitored at λ_ex_ = 495 nm and λ_em_ = 503 nm.

Subsequently, we fine-tuned the kinetics of the guest exchange reaction. As shown in Figure 3e, ^**Am2**^**dA** exhibited a slow rate for duplex rehybridization (i.e., slow kinetics for the guest exchange reaction). This was presumably attributed to the constrictive binding property of CB[7]. The carbonyl portal was narrower than the cavity, resulting in steric barriers to the guest decomplexation process.^49^ Based on this hypothesis, we designed ^**Nad**^**dA** and ^**Bic**^**dA** bearing 3-aminonoradamantane and 1-aminobicyclo[2.2.2]octane, respectively (Figure 4a). These guest moieties had a smaller molecular size than 1-aminoadamantane and were expected to pass through the carbonyl portal of CB[7] with faster kinetics. ODNs bearing ^**Nad**^**dA** and ^**Bic**^**dA** were prepared using the post-synthetic method (Table S1, Figures S1 and S2).

The base-pairing properties of ^**Nad**^**dA** and ^**Bic**^**dA** were investigated by *T*_m_ measurements of the DNA duplexes (**ODN1**/**ODN2**). These adenosine derivatives demonstrated T-selective base-pairing without compromising the thermal stability of the duplexes (Figure S10). Furthermore, ^**Nad**^**dA** and ^**Bic**^**dA** exhibited reversible base-pairing behavior owing to the host–guest interaction, as evidenced by the decrease and subsequent recovery of the *T*_m_ values after sequential treatment with CB[7] and Ad^Eda^, respectively (Figures 4b and S11). The kinetics of the reversible duplex formation by ^**Nad**^**dA** and ^**Bic**^**dA** were investigated via stopped-flow fluorescence measurements and analyzed as described above (Figures 4c, S12, and S13). The complexation rate (*k*_in_) of CB[7] with ^**Nad**^**dA**- and ^**Bic**^**dA**-modified DNAs were in the same order as that of ^**Am2**^**dA**. Contrarily, a considerable enhancement in the decomplexation rates (*k*_out_) was observed; the guest exchanging kinetics of ^**Nad**^**dA** and ^**Bic**^**dA** were 37 and 385 times faster than that of ^**Am2**^**dA**, respectively. This was presumably attributed to the smaller molecular size of noradamantane and bicyclo[2.2.2]octane, which enhanced the crossing rate through the carbonyl portal of CB[7]. Accordingly, the ^**Nad**^**dA** and ^**Bic**^**dA** showed higher *K*_d_ values than that of ^**Am2**^**dA** (Figure 4c). However, the binding affinities still lay within a nanomolar range. Owing to the improved kinetics of the guest exchange reaction, we investigated whether the guest-modified adenosines could iteratively control the duplex formation (Figure 4d). Thus, ^**Nad**^**dA**- and ^**Bic**^**dA**-containing DNAs were alternately treated with CB[7] and Ad^Eda^ at the indicated timing under isothermal conditions while monitoring the fluorescence change. For ^**Nad**^**dA**, the alternating addition of CB[7] and Ad^Eda^ induced the dissociation and rehybridization of the duplex, respectively, and the processes were repeatable for at least four cycles without noticeable degeneration. The ^**Bic**^**dA**-modified DNA exhibited reversible duplex formation repeatedly but with faster kinetics for rehybridization. The results demonstrated the robustness of the reversible duplex formation by guest-modified adenosines via the CB[7]-based host–guest interaction.

Although the present study developed guest-modified adenosine derivatives, Xiao et al. reported the formation of a reversible base pair via host–guest interaction.^50^ They attached guest moieties on the amino group of adenosine and cytidine through the C_1_-linker and demonstrated the reversible duplex formation using CB[7]. However, their nucleosides (e.g., **A**^**AD**^) inherently destabilized the duplex regardless of the host–guest interaction (Figure S14) and exhibited a low affinity for complexation with CB[7]. Contrarily, the present guest-modified adenosines displayed more natural base-pairing properties (i.e., high selectivity and stability toward pairing with thymine) and nanomolar affinity for CB[7] with tunable reversible kinetics, thereby making them more promising candidates in nucleic acid–based applications.

### Reversible Control of In Vitro Transcription

**Figure 5:**
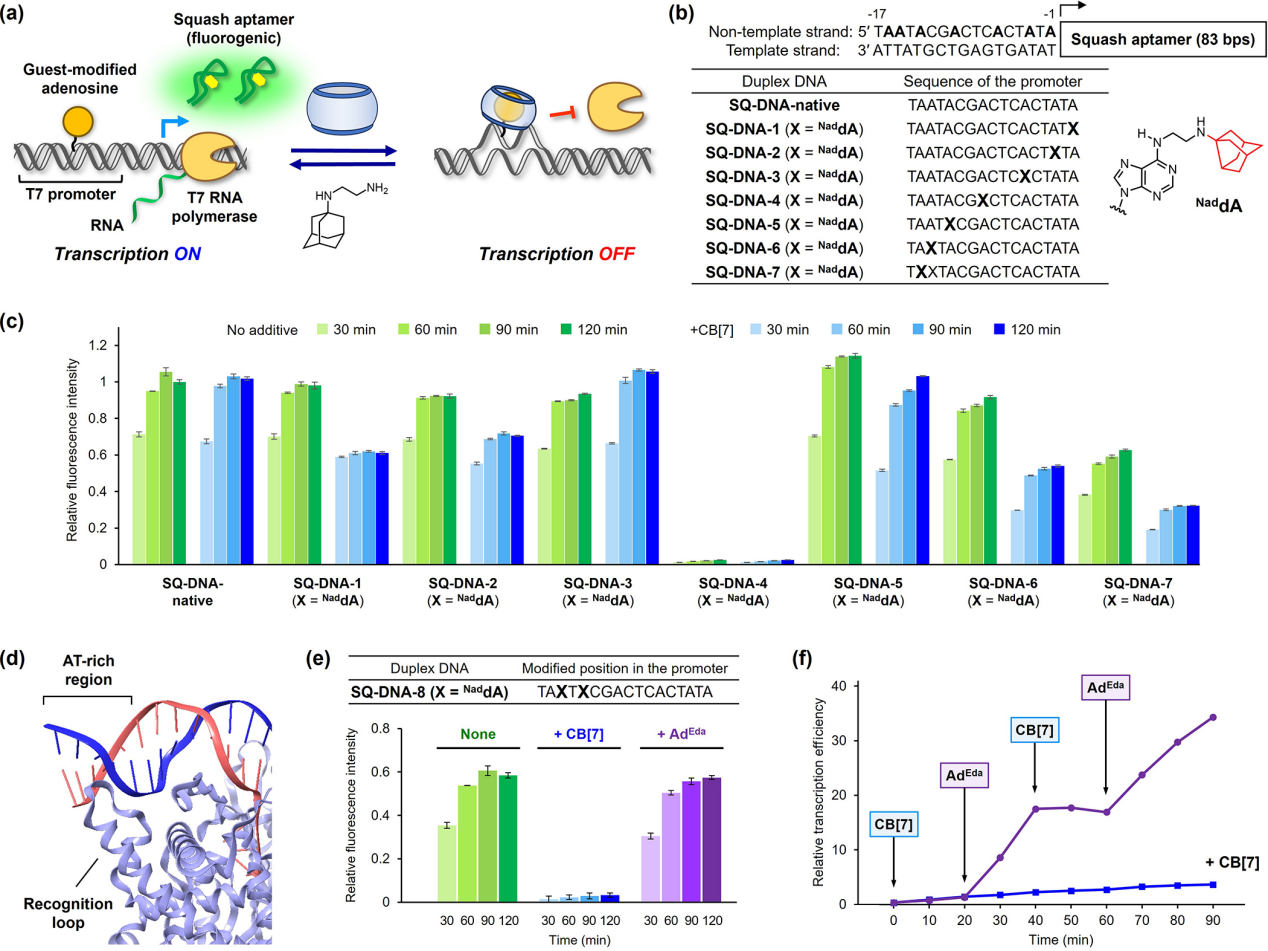
(a) Transcription switching system by T7 promoter incorporating guest-modified adenosines. Fluorescence Squash aptamer was used to monitor the efficiency of the transcription control driven by the host–guest interaction of CB[7]. (b) Sequence design of **SQ-DNA**. Each adenosine in the nontemplate strand of the T7 promoter was substituted with ^**Nad**^**dA**. (c) Time course of relative fluorescence intensity obtained from the transcription of each **SQ-DNA** (**X** = ^**Nad**^**dA**) in the absence or presence of CB[7] (8 μM). (d) Crystal structure of a T7 RNA polymerase–T7 promoter complex (PDB: 1CEZ).^52^ Interaction of the AT-rich region with the recognition loop is shown. (e) Transcription reaction of **SQ-DNA-8** (**X** = ^**Nad**^**dA**) after the sequential addition of CB[7] (8 μM) and Ad^Eda^ (10 μM). (f) Iterative transcription switching of **SQ-DNA-8** (**X** = ^**Nad**^**dA**) by the alternate addition of CB[7] (8, 20 μM) and Ad^Eda^ (10, 25 μM). The reaction in the presence of CB[7] is shown as a blue line. Conditions: DNA (1 μM) and T7 RNA polymerase ver. 2.0 (10 U/μL) in Tris–HCl buffer (40 mM, pH 8.0), dithiothreitol (5 mM), MgCl_2_ (20 mM), spermidine, and rNTP (2 mM each) at 37 °C. Fluorescence measurement of the reaction mixture was performed in the presence of DFHBI-1T at 25 °C with λ_ex_ = 451 nm and λ_em_ = 503 nm.

After the successful demonstration of the reversible duplex formation by the guest-modified adenosines, we developed the T7 RNA promoter whose activity can be controlled by the host–guest interaction. We designed an in vitro fluorescence reporter system for the convenient monitoring of promoter activity (Figure 5a). This assay system utilizes DNA comprising a chemically modified T7 RNA promoter and a fluorogenic Squash aptamer^51^ (Figure S15) and enables the quantitative analysis of promoter activity through fluorescence measurements of the transcribed aptamer in the presence of DFHBI-1T.

We initially screened suitable positions to substitute the guest-modified adenosines in the promoter sequence. Thus, each of the seven **A** in the nontemplate strand of the Squash-coding DNA (**SQ-DNA**) was replaced with ^**Nad**^**dA** (Figure 5b). The DNA duplex was prepared by the primer extension reaction of ^**Nad**^**dA**-modified **ODN5** (Table S1, Figures S1 and S2) against the 100-mer complementary template, **ODN6** (Figure S16). Transcription reactions were performed for each **SQ-DNA** with T7 RNA polymerase in the absence or presence of CB[7]. The time course of the relative transcription efficiency was determined from the fluorescence measurements of the transcribed Squash aptamer (Figure S17), and the results are summarized in Figure 5c. In the case of **SQ-DNA-native** without chemical modification, the reaction practically provided the same level of fluorescence intensity regardless of the absence or presence of CB[7]. This confirmed that CB[7] did not interfere with the transcription processes. The transcription efficiencies were subsequently investigated with ^**Nad**^**dA**-substituted **SQ-DNAs**. In the absence of CB[7], all except **SQ-DNA-4** exhibited fluorescence signals that were comparable to that of **SQ-DNA-native**, indicating the high tolerance of ^**Nad**^**dA** substitution by RNA polymerase. Oppositely, when CB[7] was added prior to the transcription, the transcription levels of the DNAs were altered. In particular, **SQ-DNA-5–7** significantly suppressed the transcription efficiency upon the addition of CB[7]. In these sequences, ^**Nad**^**dA** was substituted in the AT-rich region of the promoter, where the recognition loop of the polymerase interacted with DNA at the minor groove side (Figure 5d).^52^ Thus, the CB[7]-triggered transcription suppression with **SQ-DNA-5–7** was attributed to the partial dissociation of the DNA duplex in the AT-rich region, thereby causing the perturbation of polymerase recognition.

Having confirmed the AT-rich region as an effective modification site for transcription control, we prepared **SQ-DNA-8** incorporating two ^**Nad**^**dA** at the −13- and −15-positions from the transcription initiation site (Figures 5e and S18), anticipating a clear-cut switching of the transcription. When tested for transcription (Figures 5e and S19), despite the substitution with two unnatural nucleosides in the AT-rich region, **SQ-DNA-8** (**X** = ^**Nad**^**dA**) afforded a considerable yield of transcripts (ca. 60% compared with **SQ-DNA-native**). The addition of CB[7] reduced the transcription to a negligible level. The suppression was observed from as low as 1 μM of CB[7] and reached its maxima around 8 μM (Figure S20). Moreover, the transcription activity was fully recovered upon the addition of Ad^Eda^. Owing to these favorable results, we attempted the repetitive OFF–ON control of the transcription (Figure 5f). The alternating addition of CB[7] and Ad^Eda^ during incubation led to an iterative suppression and reactivation of the transcription, respectively. The results showed that the incorporation of guest-modified adenosines into the T7 promoter enabled the robust and reversible precise control of gene expression through host–guest interaction.

In addition to ^**Nad**^**dA**, we investigated the same transcription control using the other guest-modified adenosines. For **SQ-DNA-8** (**X** = ^**Am2**^**dA**), CB[7] again triggered the suppression of the transcription (Figure S21). Notably, the suppression effect was observed at an even lower CB[7] concentration compared with that of ^**Nad**^**dA**, presumably because of the higher affinity of ^**Am2**^**dA** with CB[7] (Figure S22). Contrarily, the recovery of the transcription activity after the Ad^Eda^ treatment was slow, consistent with the low decomplexation rate of CB[7] from ^**Am2**^**dA** (Figure 3e). **SQ-DNA-8** (**X** = ^**Bic**^**dA**) demonstrated reversible transcription control (Figure S23) while requiring a higher CB[7] concentration to achieve the same level of transcription suppression (Figure S24). The results imply that transcription levels and kinetics can be programmed by utilizing different guest-modified adenosines. Apart from our original guest-modified adenosines, we performed the same experiments using **A**^**AD**^ reported by Xiao et al.^50^ The presence of **A**^**AD**^ in the T7 promoter by itself inhibited transcription regardless of the host–guest interaction (Figure S25). These results highlight the importance of the natural-like base-pairing properties of our guest-modified nucleosides for duplex hybridization and interaction with DNA-binding proteins.

### Reversible Control of Gene Expression in a Cell-Free System

**Figure 6:**
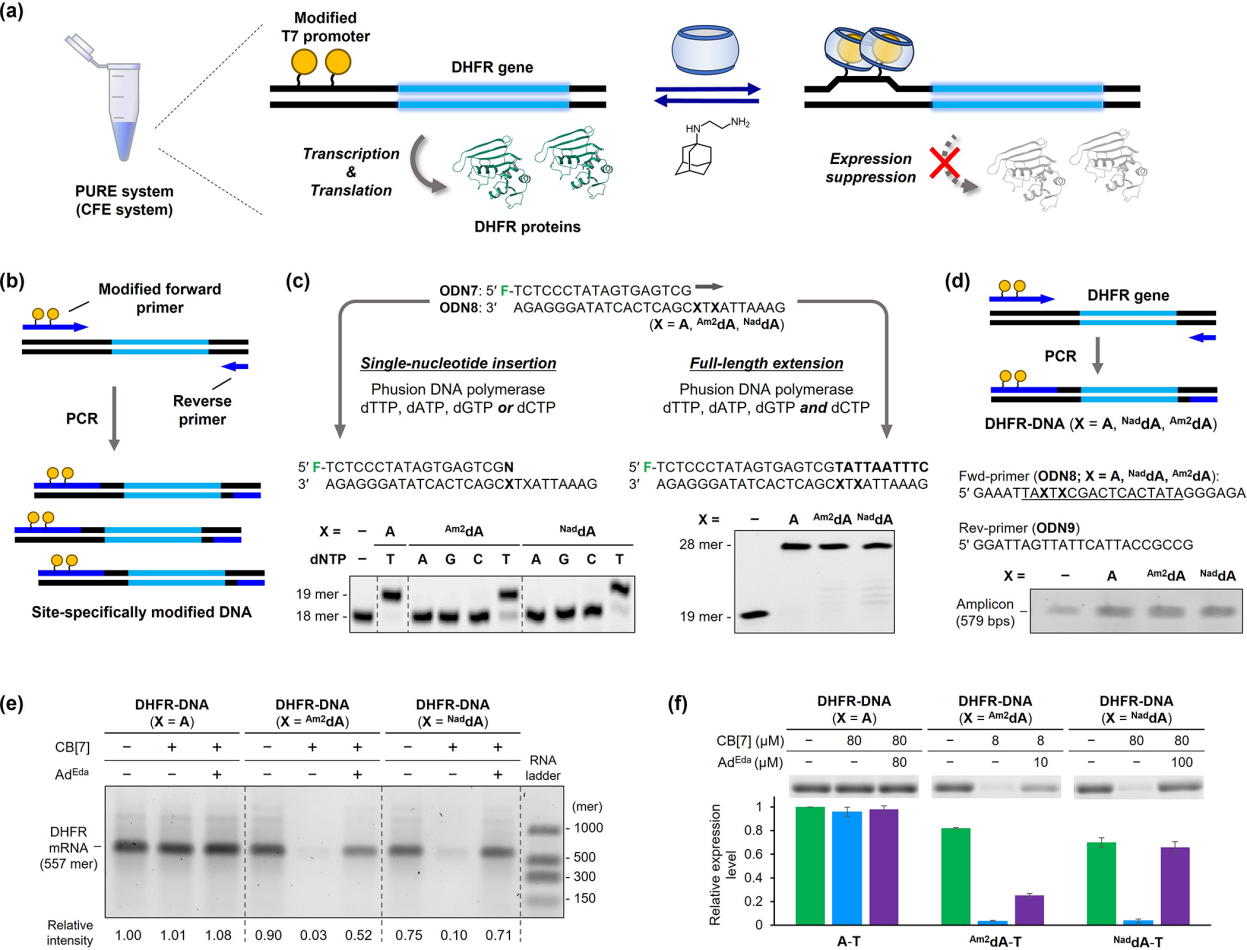
(a) Gene expression control in CFE systems. **DHFR-DNA** was endowed with the T7 promoter bearing the guest-modified adenosines and expressed DHFR proteins under the control of the host–guest interaction of CB[7]. (b) Workflow of site-specific incorporation of guest-modified adenosines into long DNA via PCR. (c) Single nucleotide insertion (left) and full-length strand elongation (right) against template **ODN8** incorporating **A**, ^**Am2**^**dA**, or ^**Nad**^**dA** at position **X**. The reaction was analyzed by denaturing PAGE. Conditions (single nucleotide insertion): **ODN7** (0.1 μM), **ODN8** (0.15 μM), and Phusion DNA polymerase (0.02 U/μL) with each dNTP (50 μM) at 37 °C for 5 min; full-length extension: **ODN7** (30 nM), **ODN8** (45 nM), and Phusion DNA polymerase (0.02 U/μL) with dNTP (400 μM each) at 55 °C for 30 min. (d) PCR-mediated preparation of **DHFR-DNA** incorporating ^**Am2**^**dA** and ^**Nad**^**dA**. PCR was conducted using **DHFR-DNA** (10 ng), **ODN8** (**X** = **A**, ^**Am2**^**dA**, and ^**Nad**^**dA**) (0.5 μM), **ODN9** (0.5 μM), dNTP (400 μM), and Phusion DNA polymerase (0.02 U/μL). The amplicons were detected via agarose-gel electrophoresis. (e) Transcription reaction of **DHFR-DNA** (**X** = **A**, ^**Am2**^**dA**, and ^**Nad**^**dA**; 10 ng) after sequential addition of CB[7] (80 μM) and Ad^Eda^ (100 μM). The reaction was performed with T7 RNA polymerase ver. 2.0 (10 U/μL) and rNTP (2 mM) at 37 °C for 2 h and analyzed by agarose-gel electrophoresis. (f) Gene expression control of **DHFR-DNA** (**X** = **A**, ^**Am2**^**dA**, and ^**Nad**^**dA**; 10 ng) in the PUREfrex system. The reaction was performed after sequentially adding CB[7] and Ad^Eda^ at 37 °C for 2 h. The relative protein expression levels were determined via agarose-gel electrophoresis using FluoroTect Green_Lys_ in vitro Translation Labeling System.

Finally, we determined if gene expression can be controlled by the modified T7 promoter in a CFE system (Figure 6a). Thus, we designed 579 bp DNA comprising the T7 promoter incorporating the guest-modified adenosines, the Shine–Dalgarno (SD) sequence, and a dihydrofolate reductase (DHFR) gene (Figure S26). The nonmodified version of the DHFR gene has been shown to express DHFR proteins in a PURE system.^2^ To prepare such a long DNA duplex incorporating the guest-modified adenosines, we considered employing a polymerase chain reaction (PCR)-mediated substitution system (Figure 6b).^17^ This method, which utilizes a chemically modified primer, was expected to provide the amplified DNA sequence while substituting the designated position with the guest-modified adenosines. Prior to testing this approach, we examined whether the guest-modified adenosines can be tolerated in a PCR through single nucleotide insertion and full-length extension or not (Figure 6c). The enzymatic reaction was performed using the PCR-compatible Phusion DNA polymerase with FAM-labeled **ODN7** and **ODN8** containing ^**Am2**^**dA** and ^**Nad**^**dA** at the +1-position from the initiation site (Table S1, Figures S1 and S2). When the single insertion was performed against **ODN8** (**X** = ^**Am2**^**dA** or ^**Nad**^**dA**), the primer was only elongated in the presence of dTTP. Furthermore, extension in the presence of all four dNTPs provided the full-length products in comparable efficiencies with fully natural **ODN8** (**X** = **A**). These results indicate that our guest-modified adenosines can function as adenosine analogues in polymerase-mediated elongation and amplification.

Thereafter, we conducted the PCR-mediated substitution reaction of **DHFR-DNA** using **ODN8** as a forward primer (Figure 6d). The ^**Am2**^**dA**- and ^**Nad**^**dA**-modified primers successfully underwent the PCR and afforded the amplified products at an efficiency that was comparable to that obtained with the nonmodified primer. The functionality of the chemoenzymatically synthesized **DHFR-DNA** was assessed via the in vitro transcription reaction using T7 RNA polymerase. The transcription efficiency was monitored by tracing the formation of the corresponding mRNA on agarose-gel (Figure 6e). **DHFR-DNA** (**X** = ^**Am2**^**dA**) and **DHFR-DNA** (**X** = ^**Nad**^**dA**) enabled the transcription of the DHFR mRNA with ∼75% efficiency of the original **DHFR-DNA** (**X** = **A**). Furthermore, the transcription activity was suppressed and recovered upon treatment with CB[7] and Ad^Eda^, respectively. **DHFR-DNA** (**X** = ^**Nad**^**dA**) exhibited a higher recovery rate than **DHFR-DNA** (**X** = ^**Am2**^**dA**). The results were consistent with the in vitro transcription reactions of **SQ-DNA-8**, confirming the successful incorporation of the guest-modified adenosines into DNA via PCR.

Finally, we investigated the protein expression control in a PURE system.^2^ The expression levels were analyzed by detecting and quantifying the translated DHFR proteins on SDS-PAGE via the in situ incorporation of fluorophore-labeled lysine. For the nonmodified **DHFR-DNA** (**X** = **A**), the expression of DHFR proteins was confirmed by a fluorescence band (Figure 6f and S27). The expression level was barely affected by the addition of CB[7] and Ad^Eda^, confirming that these additives did not interfere with the protein expression in the CFE system. Subsequently, **DHFR-DNA** (**X** = ^**Am2**^**dA**) and **DHFR-DNA** (**X** = ^**Nad**^**dA**) were tested for the CFE. Despite the chemical modification, both DNAs retained moderate levels of protein expression (>65%). Moreover, the sequential addition of CB[7] and Ad^Eda^ induced the suppression and recovery of the DHFR expression. Corresponding to the in vitro transcription results (Figure 6e), the recovery was faster with ^**Nad**^**dA**-modified DNA compared with the ^**Am2**^**dA**-modified DNA, plausibly due to the higher guest exchange rate. Furthermore, a clear correlation was observed between the protein expression levels and CB[7] concentration (Figure S28), indicating that the suppression was attributed to transcription inhibition via the host–guest interaction between the guest-modified adenosines and CB[7]. Overall, these results demonstrate the capability of the T7 promoter with guest-modified adenosines for reversible gene expression control in CFE systems.

## Conclusions

In the present study, we developed guest-modified adenosines that can reversibly control duplex formation by host–guest interaction and successfully demonstrated the reversible control of gene expression in CFE systems. When incorporated into the AT-rich region of the T7 promoter sequence, the modified adenosines efficiently suppressed the transcription through the formation of a bulky complex with CB[7], whereas complete reactivation was achieved by displacing CB[7] with the exchanging guest. Several studies have harnessed the host–guest chemistry of CB[7] to control the structures and functions of nucleic acids.^50,53−57^ However, to the best of our knowledge, this is the first report demonstrating gene expression control in a CFE system by the host–guest chemistry of CB[7]. Noteworthily, our system enabled the programming of the magnitude and rate of gene expression by selecting suitable guest structures involved in the host–guest interaction. Such a property may be useful in studying kinetically controlled transcription and genetic circuits. Thus, we expect our host–guest-based system to be a useful tool in the repertoire of cell-free regulatory gene expression research and synthetic biology.

In addition to their applications in gene expression control, our guest-modified adenosines would find applications in stimulus-responsive DNA nanotechnology,^58,59^ owing to their ability to dramatically alter duplex stability while retaining canonical A-like base-pairing abilities. In addition, the modified nucleosides were compatible with the DNA PCR. This compatibility can expand the scope of dynamic DNA nanodevices and nanostructures whose function can be regulated by host–guest interaction as a specific chemical input.

Another feature of our reversible base-pairing system is its potential applicability under biological conditions. Recent studies have demonstrated that the host–guest chemistry of CB[7] is compatible ex vivo and in vivo.^39−42^ Thus, this study has potential implications for the precise regulation of exogenous genes in living systems, leading to new approaches to precisely controlling the function of therapeutic synthetic genes. This includes the engineering of guest-modified promoters, which can drive transcription in mammalian cells. Currently, we are making efforts toward gene expression control under conditions beyond CFE systems. This will be reported in due course.

## Data Availability

All data are available in the main article and Supporting Information.

## Supporting Information Available

General protocols, synthesis of amine compounds, preparation and characterization of synthesized ODNs, and NMR charts (PDF)
