# Supplementary material for: Reversible Control of Gene Expression by Guest-Modified Adenosines in a Cell-Free System via Host–Guest Interaction
Source: J Am Chem Soc. 2024 Jun 28;146(27):18513–23. doi: 10.1021/jacs.4c04262 (PMC11240562; doi:10.1021/jacs.4c04262)
Supplement: Supplementary file 1 — ja4c04262_si_001.pdf [file ja4c04262_si_001.pdf]

Supporting information

Reversible control of gene expression by guest-modified adenosines in a cell-free system via host–guest interaction

Hidenori Okamura,[†,‡,*] Takeyuki Yao,[†,‡] Fumi Nagatsugi[†,‡,*]

[†]Institute of Multidisciplinary Research for Advanced Materials, Tohoku University, 2-1-1 Katahira, Aoba-ku, Sendai, Miyagi 980-8577, Japan
[‡]Department of Chemistry, Graduate School of Science, Tohoku University, 6-3, Aramaki Aza-Aoba, Aoba-ku, Sendai 980-8578, Japan

E-mail: hidenori.okamura.b8@tohoku.ac.jp, fumi.nagatsugi.b8@tohoku.ac.jp

1. General notes and protocols....2
2. Synthesis of the amine compounds used for post-synthetic modification....8
3. Analytical data of the ODNs used in this study....12
4. Base pairing properties of ^{Am2}dA, ^{Am3}dA, and ^{Am4}dA....20
5. Base pairing properties of ^{Et}dA and ^{AEt}dA....21
6. Reversibility and kinetic analyses of the duplex formation (^{Am2}dA, ^{Am3}dA, ^{Am4}dA)....22
7. Reversibility and kinetic analyses of the duplex formation (^{Nad}dA, ^{Bic}dA)....25
8. Reversibility and kinetic analyses of the duplex formation (A^{AD})....27
9. In vitro transcription of SQ-DNA incorporating the guest-modified adenosines....28
10. Gene expression control in CFE system using DHFR-DNA....36
11. NMR charts of the synthesized compounds....38
12. References....48

1. General notes and protocols

Materials and instrumentation

All the chemicals and solvents were purchased from commercial suppliers (FUJIFILM Wako Pure Chemical, the Tokyo Chemical Industry, Kanto Chemical, Nacalai Tesque, Strem Chemicals, BLDpharm, Glen Research, and Sigma-Aldrich) and used without further purification. The reactions were conducted under an argon atmosphere in oven-dried glassware unless otherwise specified. The NMR spectra were recorded with Bruker AVANCE III 400, Bruker AVANCE III 500 or Bruker AVANCE III 600 spectrometer. The chemical shifts were calibrated to the residual solvents as follows: ^{1}H NMR - $CDCl_3$ (7.26 ppm), MeOD-*d*$_4$ (3.31 ppm), DMSO-*d*$_6$ (2.50 ppm); ^{13}C NMR - $CDCl_3$ (77.2 ppm), MeOD-*d*$_4$ (49.0 ppm), DMSO-*d*$_6$ (39.5 ppm). All the NMR spectra were analyzed using Bruker TopSpin 3.6.2. The high-resolution electrospray ionization (ESI) mass spectrometry measurement was performed using a Bruker MicrOTOF-QII. The oligodeoxynucleotides (ODNs) were synthesized using an Applied Biosystems 392 DNA/RNA Synthesizer. The ODNs composed of all-canonical nucleosides were purchased from Japan Bio Services Co., Ltd. The HPLC purification and analysis were performed using a HPLC system composed of PU-2089 plus, UV-2075 plus, FP-2020 plus and CO-2067 plus (JASCO).The MALDI-TOF MS measurements were performed using an Autoflex speed (Bruker) utilizing a 3-hydroxypicolinic acid/diammonium hydrogen citrate matrix. UV spectroscopic measurement and UV melting temperature measurement were conducted using V-730 UV-visible spectrophotometer equipped with a PAC-743 peltier temperature cotroller (JASCO). Fluorescence spectra were measured on spectrofluorometer FP-6500 equipped with EHC-573T temperature controller (JASCO) and FP-8300 equipped with a EHC-813 temperature controller (JASCO). Stopped-flow fluorescence measurements were performed using RSP-2000 equipped with a temperature-controlled reaction chamber (Unisoku) connected with a heat bath circulator. Light was collected from 75 W xenon arc lamp housing equipped with a MD200 monochromator (Unisoku) to the reaction chamber through a bundled optical fiber. PCR was performed using T100 Thermal Cycler (Bio-Rad). The gel images were obtained and quantified by ChemiDoc MP Imaging System (Bio-Rad).

Solid-phase DNA synthesis

The ODNs were synthesized on a 1 μmol scale using an automated synthesizer with conventional phosphoramidite chemistry in DMT-OFF mode. The phosphoramidites were coupled on solid support (CPGs tethered with Pac-dA, Tac-dG, Ac-dC, T or 3'-dabcyl) using 0.25 M 5-benzylthio-1H-tetrazole (BTT) in CH_3CN as an activator, 3% dichloroacetic acid (DCA) in CH_2Cl_2 as a deblocking solution, and 5% Tac_2O and 16% *N*-methylimidazole in THF/pyridine as capping reagents. The coupling time was 30 s for the canonical nucleosides (Pac-dA, Tac-dG, Ac-dC and T phosphoramidites) and 600 s for the non-natural building blocks (6-fluorescein phosphoramidite and 6-iodopurine-2'-deoxyriboside (^{I}Pu) phosphoramidite[1]). After the synthesis, the CPGs were dried under reduced pressure overnight.

Post-synthetic conversion of ^{I}Pu into the modified adenosines in ODNs

The ODN-bound CPGs (0.1 μmol) were placed in a 1.5 mL screw-capped eppendorf tube. A solution of amine (compound **2**-**6**, 30-40% ethylamine in ethanol, or ethylenediamine; 5-20 μL) in MeOH (400 μL) was added, and the mixture was kept overnight at 50 °C in a shaker (Note: the reaction can also proceed efficiently at room temperature). After cooling to room temperature, 28% NH_4OH (400 μL) was added to the mixture, and the shaking was continued for another 2 h at room temperature. The reaction mixture was concentrated using a centrifugal evaporator. The residue was diluted with distilled H_2O and washed twice with EtOAc. The aqueous layer was collected, filtered through a filter unit (DISMIC-13HP, ADVANTEC) and evaporated under reduced pressure. The crude ODN was purified by reverse-phase HPLC using a Nacalai Tesque COSMOSIL 5C_{18}-MS-II column (4.6ID × 250 mm) with 0.1 M triethylammonium acetate buffer at pH 7.0 (buffer A) and CH_3CN (buffer B). A linear gradient of 5 to 25% of buffer B over 20 min was applied at the flow rate of 3 mL/min except for ODN1 (X = A^{AD}) and ODN5-8 (X = A^{AD}) in which a linear gradient of 10 to 35% for over 20 min and 10 to 60% for over 20 min was utilized. The column oven was set to 50 °C, and the peaks were detected at 254 nm. The appropriate fraction was collected and lyophilized. The purified ODN was dissolved in distilled H_2O and ion-exchanged using Strong Acidic Cation Exchange Resin No.6 (Na^+-form, FUJIFILM Wako Pure Chemical). The eluent was desalted by passing through Nap-10 column (Cytiva) and lyophilized. The purity and structural integrity of each synthesized ODN were confirmed by RP-HPLC and MALDI-TOF MS analyses, respectively.

Enzymatic digestion of the ODNs incorporating guest-modified nucleosides

To a solution of ODN (2 nmol) in 1× Nucleoside Digestion Mix Reaction Buffer (New England Biolabs) was added Nucleoside Digestion Mix (4 μL, New England Biolabs), and the reaction mixture (40 μL in total) was incubated at 37 °C for 4 h. The digested mixture was analyzed by reverse-phase HPLC using a Nacalai Tesque COSMOSIL 5C_{18}-MS-II column (4.6ID × 250 mm) with 50 mM ammonium formate (buffer A) and CH_3CN (buffer B). A linear gradient of 5 to 15% over 10 min followed by 15 to 40% over 20 min of buffer B was applied at the flow rate of 1 ml/min at 35°C, and the peaks were detected at 254 nm. For A^{AD}-containing ODNs, a linear gradient of 5 to 15% over 10 min followed by 15 to 100% over 20 min of buffer B was applied.The appropriate fraction was collected and analyzed by ESI-TOF MS measurement.

UV melting temperature measurement of duplex DNA

A solution containing 2 μM of each ODN, 10 mM sodium phosphate buffer (pH 7.0), and 150 mM NaCl was heated at 90 °C and gradually cooled down to room temperature for annealing. UV melting curves were recorded with a quartz cell with a 1 cm path length at temperatures between 10 and 80 °C with a ramping and a scanning rate of 1.0 °C/min at 260 nm. The melting curves from alternating addition of CB[7] and Ad^{Eda} were recorded after incubating the ODN solution with 4 μM of CB[7] for 30 min and 10 μM of Ad^{Eda} for 30 h at 37 °C. Each T_m value is presented as an average of three measurements. The absorbance was normalized using the following equation: Normalized Abs_{260} = {Abs_{260}(t °C)-Abs_{260}(10 °C)} / {Abs_{260}(80 °C) – Abs_{260}(10 °C)}.

Time course monitoring of the fluorescence upon the addition of CB[7]

A solution of duplex DNA was prepared by annealing **ODN3** (0.2 μM) and **ODN4** (0.3 μM) in 10 mM sodium phosphate buffer (pH 7.0) and 150 mM NaCl as described above. The buffered duplex solution (**ODN3-ODN4**) and CB[7] solution (2 μM in 10 mM sodium phosphate buffer and 150 mM NaCl at pH 7.0) were set in each chamber. After the thermal equilibration at 37 °C, an equal volume (~60 μL) of each solution was mixed in the reaction chamber at 37 °C, and the reaction progress was followed by monitoring the increase in the fluorescence (λ_{ex} = 495 nm, λ_{em} > 525 nm). Relative fluorescence intensity was defined as the ratio of the fluorescence intensity with respect to that of the end of the measurement.

Time course monitoring of the fluorescence upon the addition of Ad^{Eda}

A solution of the duplex DNA (prepared as described above) in 10 mM sodium phosphate buffer (pH 7.0), 150 mM NaCl and CB[7] (2 μM) was incubated at at 37 °C for 30 min. The duplex-CB[7] buffer solution and Ad^{Eda} buffer solution (4 μM in 10 mM sodium phosphate buffer (pH 7.0) and 150 mM NaCl) were set in each chamber. After the thermal equilibration at 37 °C, an equal volume (~60 μL) of each solution was mixed in the reaction chamber at 37 °C, and the reaction progress was followed by monitoring the decrease in the fluorescence with λ_{ex} = 495 nm and λ_{em} > 525 nm. In case of ^{Am2}dA, the time-course change of the fluorescence monitored under the same conditions except that the measurement was performed on a spectrofluorometer FP-8300 (JASCO; λ_{ex} = 495 nm and λ_{em} = 520 nm) due to its inherently slow reaction rate. Relative fluorescence intensity was defined as the ratio of the fluorescence intensity with respect to that in the beginning of the measurement.

Curve fitting for calculating apparent rate constant

(i) Reaction rate for duplex dissociation process

Assuming that the dissociation of CB[7] from the guest-modified adenosines are considerably slow under the given conditions, the duplex dissociation reaction induced by CB[7] binding to the guest-modified DNA can be approximated as a second-order reaction, and the rate equation at a given time "t" for DNA + CB → DNA · CB (complex of DNA and CB[7]) can be described as follows:

$$[\mathbf{DNA}\cdot\mathbf{CB}] = \frac{[\mathbf{DNA}]_0[\mathbf{CB}]_0\big((\mathbf{1}-\mathbf{exp}(([\mathbf{DNA}]_0-[\mathbf{CB}]_0)\,\boldsymbol{k_{in}t})\big)}{[\mathbf{CB}]_0-[\mathbf{DNA}]_0\,\mathbf{exp}\big(([\mathbf{DNA}]_0-[\mathbf{CB}]_0)\boldsymbol{k}_{\mathbf{in}}\boldsymbol{t}\big)} \qquad (\mathbf{1})$$

$[\mathrm{DNA}]_0$, $[\mathrm{CB}]_0$: initial concentrations of DNA and CB[7], k_{in}: the rate constant for duplex dissociation

In the case [CB7] ≫ [DNA], the reaction can be approximated as pseudo-first order reaction as follows:

$$[\mathbf{DNA}\cdot\mathbf{CB}] = [\mathbf{DNA}]_0(\mathbf{1}-\mathbf{exp}(-[\mathbf{CB}]_0\boldsymbol{k}_{\mathbf{in}}\boldsymbol{t})) \qquad (\mathbf{2})$$

By defining the constant as the fluorescence intensity at the end of the reaction as "b" and the fluorescence intensity at the start of the reaction as "a + b", the fitting equation can be expressed as in equation (3).

$$\boldsymbol{y} = \boldsymbol{a}\,\mathbf{exp}(-\boldsymbol{k}_{\mathbf{in}}[\mathbf{CB}]_0\boldsymbol{t}) + \boldsymbol{b} \qquad (\mathbf{3})$$

(ii) Reaction rate for duplex re-hybridization process

The duplex re-formation reaction by the addition of the exchange guest is the rate-determining reaction for the dissociation process of the CB[7]-guest complex. Therefore, this reaction can be approximated as a first-order reaction of the CB[7]-guest complex. The first-order reaction equation at a certain time "t" is expressed as in equation (4).

$$[\mathbf{DNA}] = [\mathbf{DNA}\cdot\mathbf{CB}]_{\mathbf{0}}(\mathbf{1} - \mathbf{exp}(-\boldsymbol{k}_{\mathbf{out}}\boldsymbol{t})) \qquad (\mathbf{4})$$

$[DNA \cdot CB]_0$ is the initial concentration of DNA·CB[7] complex, k_{out} is the reaction rate constant

Defining the fluorescence intensity at the end of the reaction as "b" and the fluorescence intensity at the beginning of the reaction as "a + b" as constants, the curve fitting equation is expressed as in equation (5).

$$\boldsymbol{y} = \boldsymbol{a}\,\mathbf{exp}(-\boldsymbol{k}_{\mathbf{out}}\boldsymbol{t}) + \boldsymbol{b} \qquad (\mathbf{5})$$

Preparation of SQ-DNAs by polymerase extension reaction

A solution of **ODN5** (4 μM) and the 100 mer template **ODN6** (2 μM) in 1× ThermoPol Reaction Buffer (New England Biolabs) was mixed with dNTP (400 μM each, Toyobo) and Deep Vent (exo^-) polymerase (0.04 U/μL, New England BioLabs) on ice. The reaction mixture was heated at 95 °C for 20 s followed by the incubation at 50 °C for 30 min. An aliquot (2 μL) of the reaction mixture was mixed with a loading buffer (18 μL, 2.5× TBE buffer and 50% glycerol) and analyzed on 20% polyacrylamide native gel at 200 V. The bands were stained with SYBR Gold Nucleic Acid Gel Stain (Invitrogen) and visualized using ChemiDoc MP Imaging System with a SYBR Gold filter. The extended duplex DNA (**SQ-DNA**) was purified by QIAquick Gel Extraction Kit (Qiagen) according to the manufacturer's protocol.

In vitro transcription of SQ-DNA and fluorescnce measurement of transcribed Squash aptamer

A solution of 1 μM **SQ-DNA**, 40 mM Tris-HCl buffer (pH 8.0), 5 mM dithiothreitol, 20 mM $MgCl_2$, 2 mM spermidine and rNTP (2 mM each) was mixed with T7 RNA Polymerase ver 2.0 (10 U/μL, Takara Bio), and the reaction mixture was incubated at 37 °C. Part of the reaction mixture (2 μL) was taken at each time point (0, 30, 60, 90, 120 min) and mixed with a solution (98 μL) of 5 μM DFHBI-1T, 40 mM HEPES-KOH (pH 7.4), 100 mM KCl and 0.5 mM $MgCl_2$. Fluorescence spectra were measured on a FP-6500 spectrofluorometer with λ_{ex} = 451 nm and λ_{em} = 503 nm at 25 °C. The transcription reactions in the presence of CB[7] and Ad^{Eda} were performed in the same manner except that the reaction mixture was incubated with CB[7] (8 μM) at 37 °C for 30 min prior to the addition of T7 RNA polymerase. Ad^{Eda} (10 μM) was added immediately before the addition of T7 RNA polymerase. For the iterative control of transcription activity, CB[7] and Ad^{Eda} were added to the reaction mixture at the indicated timing.

Single-nucleotide insertion against the guest-modified adenosines

To a solution of the FAM-labelled **ODN7** (0.1 μM), **ODN8** (0.15 μM) and Phusion DNA polymerase (0.02 U/μL, New England BioLabs) in 1× Phusion HF Buffer (New England Biolabs) was added each dNTP (50 μM) on ice, and the reaction mixture (10 μL in total) was incubated at 37 °C for 5 min. An aliquot (4 μL) of the reaction mixture was mixed with a loading buffer (8 μL, 95% formamide containing 20 mM EDTA) and heated at 95 °C for 5 min. The products were analyzed on 20% polyacrylamide denaturing gel containing 7 M urea at 300 V. The gel images were obtained using ChemiDoc MP Imaging System with a Alexa 488 filter.

Full-length extension using the template ODN containing the guest-modified adenosines

A solution of the FAM-labelled **ODN7** (30 nM) and **ODN8** (45 nM) in 1× Phusion HF Buffer was mixed with dNTP (400 μM each) and Phusion DNA polymerase (0.02 U/μL) on ice, and the reaction mixture (40 μL in total) was incubated at 55 °C for 30 min. An aliquot (12 μL) of the reaction mixture was mixed with a loading buffer (12 μL, 95% formamide containing 20 mM EDTA) and heated at 95 °C for 5 min. The products were analyzed on 20% polyacrylamide denaturing gel as described above.

Preparation of the modified DHFR gene by PCR

A solution (20 μL) of **DHFR-DNA**[2] (10 ng, GeneFrontier), **ODN8** (0.5 μM, Fwd-primer), **ODN9** (0.5 μM, Rev-primer), dNTP (400 μM each) and Phusion DNA Polymerase (0.02 U/μL) in 1× Phusion HF Buffer was prepared on ice. The PCR was performed using a T100 Thermal Cycler (Bio-Rad) with the following cycle: 95 °C for 10 s, 56 °C for 20 s, 70 °C for 30 s (20 cycles). The reaction mixture was diluted 200-fold with distilled H_2O and subjected to another round (20 cycles) of PCR under the same conditions as described above. The reaction mixture was mixed with 10× Loading Buffer (5 μL, Takara Bio) and analyzed by 1.5% agarose gel electrophoresis at 100 V. The bands were stained with SYBR Gold Nucleic Acid Gel Stain and visualized using ChemiDoc MP Imaging System with a SYBR Gold filter. Alternatively, the PCR products were visualized by UV shadowing for gel purification. The appropriate band was excised from the agarose gel, and the amplified DNA was isolated using NucleoSpin Gel and PCR Clean-up kit (MACHEREY-NAGEL) according to the manufacturer's protocol.

In vitro transcription of DHFR-DNA

A solution of DHFR-DNA (10 ng), 40 mM Tris-HCl buffer (pH 8.0), 5 mM dithiothreitol, 20 mM $MgCl_2$, 2 mM spermidine, RNase Inhibitor (1.75 U/μL, Nacalai Tesque) and rNTP (2 mM each, New England Biolabs) was mixed with T7 RNA Polymerase ver 2.0 (10 U/μL), and the reaction mixture (20 μL in total) was incubated at 37 °C for 2 h. The reaction was quenched with 10× Loading Buffer (2 μL, Takara Bio), and the products were analyzed by 1.5% agarose gel elctrophoresis at 100 V. The gel was run with Low Range ssRNA Ladder (New England Biolabs). The bands were stained with SYBR Gold Nucleic Acid Gel Stain and visualized using ChemiDoc MP Imaging System with a SYBR Gold filter. The transcription reactions in the presence of CB[7] and Ad^{Eda} were performed in the same manner except that the reaction mixture

was incubated with CB[7] (80 μM) at 37 °C for 30 min prior to the addition of T7 RNA polymerase. Ad^{Eda} (100 μM) was added immediately before the addition of T7 RNA polymerase.

Cell-free protein expression

Cell-free protein expression was conducted using Purefrex 1.0 (Solution I to III, GeneFrontier) with FluoroTect™ $Green_{Lys}$ in vitro Translation Labeling System (Promega) to quantify the expressed protein by fluorescence. A mixture (10 uL) of the DHFR-DNA (10 ng), Solution I (5 μL), Solution II (0.5 μL), Solution III (0.5 μL), FluoroTect $Green_{Lys}$ tRNA (0.5 μL) and RNase Inhibitor (1.75 U/μL) was incubated at 37 °C. After the indicated reaction time, RNase A Solution (1 μL, Promega) was added, and the reaction mixture was incubated at 37 °C for additional 15 min to digest the unreacted FluoroTect™ $Green_{Lys}$ tRNA. The digest was mixed with Sample Buffer Solution without 2-ME (2x) for SDS-PAGE (11 μL, Nacalai Tesque) and heated at 95 °C for 3 min. The sample was analyzed by SDS-PAGE using SuperSep Ace, 12.5%, 17 well (FUJIFILM Wako Pure Chemical) at 300 V. The gel image was obtained using ChemiDoc MP Imaging System with an Alexa 488 filter. The reactions in the presence of CB[7] and Ad^{Eda} were performed in the same manner except that a mixture of DNA, CB[7] (8 or 80 μM) and Solution I was incubated at 37 °C for 30 min before the addition of the other reagents. Ad^{Eda} (10 or 100 μM) was added immediately before the addition of Solution II, Solution III, FluoroTect $Green_{Lys}$ tRNA and RNase inhibitor.

2. Synthesis of the amine compounds used for post-synthetic modification

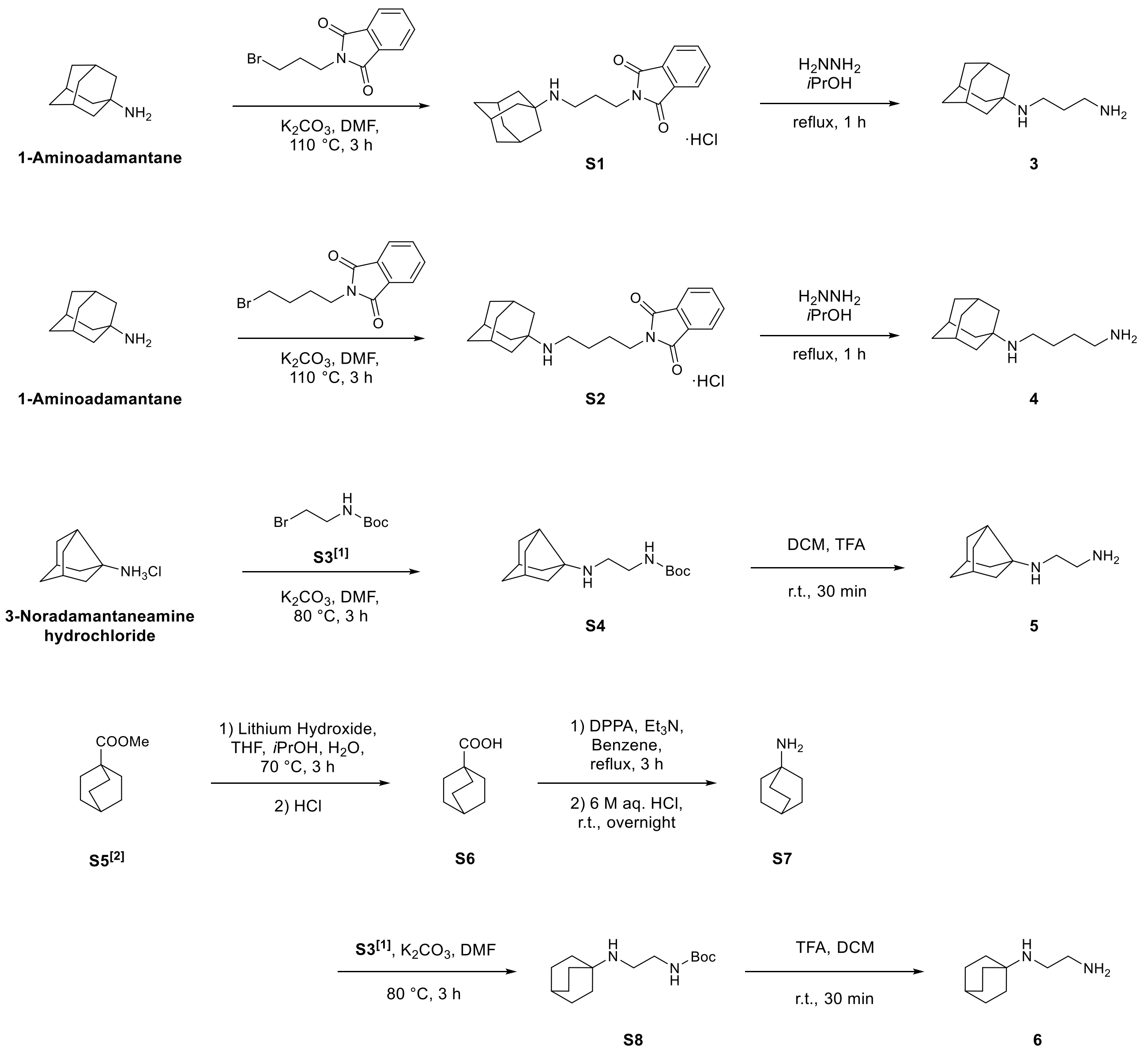

Scheme S1. Synthesis of the amine compounds. *N*-(1-Adamantyl)ethylenediamine (**2**), ethylamine (30-40% in ethanol), ethylenediamine were purchased from the Tokyo Chemical Industry.

N-(1-Adamantylaminopropyl)phthalimide hydrochloride (**S1**)

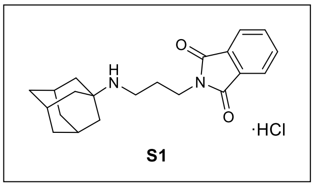

To a solution of 1-aminoadamantane (1.51 g, 9.98 mmol, 2.0 equiv.) and *N*-(3-bromopropyl)phthalimide (1.34 g, 5.00 mmol, 1.0 equiv.) in DMF (10 mL) was added K_2CO_3 (691 mg, 5.00 mmol, 1.0 equiv.), and the mixture was stirred at 110 °C for 3 h. The resulting suspension was filtered, and the filtrate was evaporated under reduced pressure. The residue was treated with 0.2 M HCl and filtered. The filter cake was washed with ether and dried in vacuo to afford **S1** as a white powder (1.51 g, 4.03 mmol, 81%).

^{1}H NMR ($CDCl_3$, 400 MHz) δ 7.86 (2H, dd, *J* = 5.4, 3.0 Hz), 7.75 (2H, dd, *J* = 5.4, 3.0 Hz), 3.90 (2H, t, *J* = 6.2 Hz), 2.93 (2H, t, *J* = 6.2 Hz), 2.43-2.41 (2H, m), 2.22-2.16 (3H, m), 2.08-1.97 (6H, m), 1.73-1.68 (6H, m). ^{13}C NMR ($CDCl_3$, 126 MHz) δ 168.8, 134.4, 132.1, 123.7, 57.8, 38.8, 37.7, 35.7, 35.2, 29.2, 26.7. ESI-HRMS (*m*/*z*): [M+H]$^+$ calcd. for $C_{21}H_{27}N_2O_2^+$ 339.2067, found 339.2071.

N-(1-Adamantyl)propane-1,3-diamine (**3**)

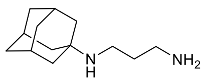

3

To a solution of **S1** (800 mg, 2.13 mmol, 1.0 equiv.) in *i*PrOH (3.54 mL) was added hydrazine monohydrate (172 μL, 3.55 mmol, 1.7 equiv.), and the mixture was refluxed for 5 h. The resulting suspension was filtered, and the filtrate was concentrated under reduced pressure. The residue was purified by silica gel column chromatography (NH_2-silica gel, DCM:MeOH = 20:1 to 5:1) to afford **3** as a pale yellow oil (393.0 mg, 1.89 mmol, 89%).

^{1}H NMR (DMSO-*d$_6$*, 400 MHz) δ 2.58-2.53 (2H, m), 2.52-2.48 (2H, m), 1.99 (3H, s), 1.63-1.54 (12H, m), 1.42-1.38 (2H, quin., J = 4.6 Hz). ^{13}C NMR (DMSO-*d$_6$*, 151 MHz) δ 49.6, 42.3, 40.1, 37.7, 36.4, 34.7, 29.0. ESI-HRMS (*m*/*z*): [M+H]$^+$ calcd. for $C_{13}H_{25}N_2^+$ 209.2012, found 209.2012.

N-(1-Adamantylaminobutyl)phthalimide hydrochloride (**S2**)

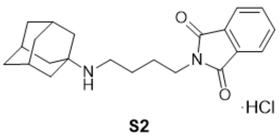

S2

To a solution of *N*-(4-bromobutyl)phthalimide (1.41 g, 5.00 mmol, 1.0 equiv.) and 1-aminoadamantane (1.51 g, 9.98 mmol, 2.0 equiv.) in DMF (10 mL) was added K_2CO_3 (691 mg, 5.00 mmol, 1.0 equiv.), and the mixture was stirred at 110 °C for 3 h. The resulting suspension was filtered, and the filtrate was evaporated under reduced pressure. The residue was treated with 0.2 M HCl and filtered. The filter cake was washed with ether and dried in vacuo to afford **S2** as a white powder (1.55 g, 3.99 mmol, 80%).

^{1}H NMR (DMSO-*d$_6$*, 400 MHz) δ 8.73 (2H, brs), 7.90-7.82 (4H, m), 3.61 (2H, t, *J* = 5.8 Hz), 2.97-2.83 (2H, m), 2.13-2.07 (3H, m), 1.87-1.82 (6H, m), 1.72-1.54 (10H, m). ^{13}C NMR (DMSO-*d$_6$*, 126 MHz) δ 168.0, 134.5, 131.6, 123.1, 55.9, 38.3, 37.5, 36.9, 35.2, 28.4, 25.3, 23.7. ESI-HRMS (*m*/*z*): [M+H]$^+$ calcd. for $C_{22}H_{29}N_2O_2^+$ 353.2224, found 353.2223.

N-(1-Adamantyl)butane-1,4-diamine (**4**)

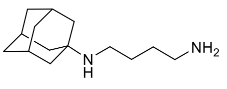

4

To a solution of **S2** (800 mg, 2.06 mmol, 1.0 equiv.) in *i*PrOH (3.40 mL) was added hydrazine monohydrate (165 μL, 3.40 mmol, 1.7 equiv.), and the mixture was refluxed for 2 h. The resulting suspension was filtered, and the filtrate was concentrated under reduced pressure. The residue was purified by silica gel column chromatography (NH_2-silica gel, DCM:MeOH = 20:1) to afford **4** as a pale yellow oil (446.8 mg, 2.01 mmol, 98%).

^{1}H NMR (DMSO-*d$_6$*, 600 MHz) δ 2.50 (2H, t, *J* = 4.4 Hz), 2.46 (2H, t, *J* = 4.4 Hz), 2.02-1.97 (3H, m), 1.64-1.53 (12H, m), 1.37-1.32 (4H, m). ^{13}C NMR (DMSO-*d$_6$*, 151 MHz) δ 49.5, 42.4, 41.8, 39.8, 36.4, 31.5, 29.0, 28.3. ESI-HRMS (*m*/*z*): [M+H]$^+$ calcd. for $C_{14}H_{27}N_2^+$ 223.2169, found 223.2181.

N-Boc-*N*-(3-noradamantyl)ethylenediamine (**S4**)

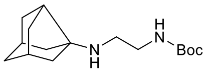

S4

To a solution of **S3**[3] (302.3 mg, 1.35 mmol, 1.0 equiv.) and 3-noradamantanamine hydrochloride (234 mg, 1.35 mmol, 1.0 equiv.) in DMF (2.7 mL) was added K_2CO_3 (745 mg, 5.40 mmol, 4.0 eqiuv.), and the mixture was stirred at 80 °C for 3 h. The resulting suspension was filtered, and the filtrate was concentrated under reduced pressure. The residue was purified by silica gel column chromatography (DCM:MeOH = 100:0 to 10:1 with 1% triethylamine) to afford **S4** as a white powder (166.6 mg, 0.594 mmol, 44%).

^{1}H NMR (MeOD-d_4, 400 MHz) δ 3.18 (2H, t, *J* = 6.4 Hz), 2.70 (2H, t, *J* = 6.6 Hz), 2.27-2.23 (2H, m), 2.11-2.08 (1H, m), 1.91-1.88 (2H, m), 1.82-1.76 (4H, m), 1.67-1.53 (4H, m), 1.44 (9H, s). ^{13}C NMR (MeOD-d_4, 151 MHz) δ 158.6, 80.1, 69.5, 47.1, 45.1, 44.5, 43.6, 41.6, 38.8, 36.0, 28,7. ESI-HRMS (*m*/*z*): [M+H]$^+$ calcd. for $C_{16}H_{29}N_2O_2^+$ 281.2224, found 281.2238.

N-(3-Noradamantyl)ethylenediamine (**5**)

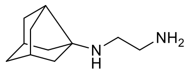

5

To a solution of **S4** (158.1 mg, 0.564 mmol, 1.0 equiv.) in DCM (2.8 mL) was added trifluoroacetic acid (2.8 mL), and the mixture was stirred at room temperature for 30 min. The resulting solution was concentrated under reduced pressure. The residue was purified by silica gel column chromatography (NH_2-silica gel, DCM:MeOH = 50:1 to 10:1) to afford **5** as a pale yellow oil (98.7 mg, 0.547 mmol, 96%).

^{1}H NMR (MeOD-d_4, 400 MHz) δ 2.79-2.73 (2H, m), 2.71-2.66 (2H, m), 2.28-2.23(2H, m), 2.15-2.09 (1H, m), 1.95-1.86 (2H, m), 1.84-1.77 (4H, m), 1.70-1.53 (4H, m). ^{13}C NMR ($CDCl_3$, 151 MHz) δ 68.5, 49.1, 47.2, 43.9, 42.9, 42.5, 37.6, 35.2. ESI-HRMS (*m*/*z*): [M+H]$^+$ calcd. for $C_{11}H_{21}N_2^+$ 181.1699, found 181.1692.

1-Bicyclo[2.2.2]octane carboxylic acid (**S6**)

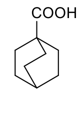

S6

To a solution of **S5**[4] (1.03 g, 6.15 mmol, 1.0 equiv.) in a mixture of THF (10.2 mL), *i*PrOH (3.1 mL) and H_2O (10.2 mL) was added lithium hydroxide monohydrate (0.774 g, 18.4 mmol, 3.0 equiv.), and the reaction mixture was stirred at 60 °C for 3 h. The resulting solution was diluted with EtOAc. The organic layer was washed with H_2O, brine, dried over Na_2SO_4 and filtered. The solvent was removed under reduced pressure to afford **S6** as a white powder (0.893 g, 5.79 mmol, 94%).

^{1}H NMR (DMSO-d_6, 400 MHz) δ 11.92 (1H, brs), 1.64-1.60 (6H, m), 1.57-1.55 (1H, m), 1.53-1.49 (6H, m). ^{13}C NMR (MeOD-d_4, 151 MHz) δ 182.2, 39.1, 29.2, 26.4, 25.2. ESI-HRMS (*m*/*z*): [M-H]$^-$ calcd. for $C_9H_{13}O_2^-$ 153.0921, found 153.0916.

1-Bicyclo[2.2.2]octanamine (**S7**)

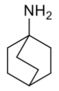

S7

To a solution of **S6** (191 mg, 1.24 mmol, 1.0 equiv.) in dry benzene (12.4 mL) was added triethylamine (259 μL, 1.86 mmol, 1.5 equiv.) and diphenylphosphoryl azide (324 μL, 1.49 mmol, 1.2 equiv.), and the reaction mixture was refluxed for 3 h. The volatiles were removed under reduced pressure. The residue was dissolved in 6 M HCl (12 mL) and Et_2O (6 mL), and the mixture was stirred overnight at room temperature. The aqueous layer was extracted with Et_2O, basified with Na_2CO_3, and extracted with DCM. The organic layer was dried over Na_2SO_4 and concentrated under reduced pressure. The residue was purified by silica gel column chromatography (NH_2-silica gel, DCM:MeOH = 50:1) to afford **S7** as a white powder (102 mg, 0.815 mmol, 66%).

^{1}H NMR (MeOD-*d$_4$*, 400 MHz) δ 1.69-1.63 (6H, m), 1.55-1.47 (7H, m). ^{13}C NMR (MeOD-*d$_4$*, 151 MHz) δ 47.6, 34.7, 27.6, 25.4. ESI-HRMS (*m*/*z*): [M+H]$^+$ calcd. for $C_8H_{16}N^+$ 126.1277, found 126.1293.

N-Boc-*N*-(1-bicyclo[2.2.2]octanyl)ethylenediamine (**S8**)

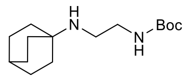

S8

To a solution of **S7** (106 mg, 0.843 mmol, 1.0 equiv.) and **S3**[1] (190 mg, 0.843 mmol, 1.0 equiv.) in DMF (4.2 mL) was added K_2CO_3 (467 mg, 3.37 mmol, 4.0 equiv.), and the mixture was stirred at 80 °C for 3 h. The suspension was filtered, and the filtrate was concentrated under reduced pressure. The residue was purified by silica gel column chromatography (DCM:MeOH = 50:1 to 10:1 with 1% triethylamine) to afford **S8** as a white powder (60.4 mg, 0.211 mmol, 20%).

^{1}H NMR (MeOD-*d$_4$*, 400 MHz) δ 3.10 (2H, t, *J* = 6.4 Hz), 2.57 (2H, t, *J* = 6.6 Hz), 1.68-1.64 (6H, m), 1.54-1.50 (7H, m), 1.43 (9H, s). ^{13}C NMR (MeOD-*d$_4$*, 151 MHz) δ 158.6, 80.0, 51.4, 42.3, 41.7, 31.3, 28.7, 27.3, 25.3. ESI-HRMS (*m*/*z*): [M+H]$^+$ calcd. for $C_{15}H_{29}N_2O_2^+$ 269.2224, found 269.2253.

N-(1-Bicyclo[2.2.2]octanyl)ethylenediamine (**6**)

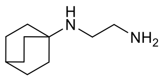

6

To a solution of **S8** (135 mg, 0.503 mmol, 1.0 equiv.) in DCM (3.1 mL) was added trifluoroacetic acid (3.1 mL), and the mixture was stirred at room temperature for 30 min. The resulting solution was concentrated under reduced pressure. The crude residue was purified by silica gel column chromatography (NH_2-silica gel, DCM:MeOH = 100:0 to 10:1) to afford **6** as a pale yellow oil (67.8 mg, 0.403 mmol, 80%).

^{1}H NMR (MeOD-*d$_4$*, 400 MHz) δ 2.70 (2H, t, *J* = 6.2 Hz), 2.57 (2H, t, *J* = 6.4 Hz), 1.67-1.53 (13H, m). ^{13}C NMR (MeOD-*d$_4$*, 151 MHz) δ 51.5, 44.6, 42.6, 31.3, 27.4, 25.3. ESI-HRMS (*m*/*z*): [M+H]$^+$ calcd. for $C_{10}H_{21}N_2^+$ 169.1699, found 169.1706.

3. Analytical data of the ODNs used in this study

Table S1. Sequences and MADLI-TOF MS data of the ODNs used in this study.

Sequence name	Sequence (5' to 3')	Yield (%)[d]	Calcd. $[M-H]^-$	Found (*m*/*z*)
**ODN1** (**X** = **A**)[a]	CTCAG **A** GTAAGG	–	–	–
**ODN1** (**X** = ^{Am2}**dA**)	CTCAG ^{Am2}**dA** GTAAGG	26%	3868.78	3868.34
**ODN1** (**X** = ^{Am3}**dA**)	CTCAG ^{Am3}**dA** GTAAGG	17%	3882.80	3882.76
**ODN1** (**X** = ^{Am4}**dA**)	CTCAG ^{Am4}**dA** GTAAGG	7%	3896.81	3897.54
**ODN1** (**X** = ^{Et}**dA**)	CTCAG ^{Et}**dA** GTAAGG	31%	3719.66	3719.59
**ODN1** (**X** = ^{AEt}**dA**)	CTCAG ^{AEt}**dA** GTAAGG	31%	3734.67	3735.38
**ODN1** (**X** = ^{Nad}**dA**)	CTCAG ^{Nad}**dA** GTAAGG	32%	3854.27	3855.27
**ODN1** (**X** = ^{Bic}**dA**)	CTCAG ^{Bic}**dA** GTAAGG	21%	3719.66	3719.59
**ODN1** (**X** = **A**^{AD})	CTCAG **A**^{AD} GTAAGG	15%	3839.76	3839.62
**ODN2** (**Y** = **A**)[a]	CCTTAC A CTGAG	–	–	–
**ODN2** (**Y** = **G**)[a]	CCTTAC G CTGAG	–	–	–
**ODN2** (**Y** = **C**)[a]	CCTTAC C CTGAG	–	–	–
**ODN2** (**Y** = **T**)[a]	CCTTAC T CTGAG	–	–	–
**ODN3** (**X** = ^{Am2}**dA**)[b]	**F**-CTCAG ^{Am2}**dA** GTAAGG	17%	4407.91	4407.15
**ODN3** (**X** = ^{Nad}**dA**)[b]	**F**-CTCAG ^{Nad}**dA** GTAAGG	11%	4393.90	4393.53
**ODN3** (**X** = ^{Bic}**dA**)[b]	**F**-CTCAG ^{Bic}**dA** GTAAGG	20%	4381.90	4382.27
**ODN4** (**Y** = **T**)[a,c]	CCTTAC T CTGAG-**Q**	–	–	–
**ODN5-native**[a]	TAATACGACTCACTATAGG	–	–	–

**ODN5-1** (**X** = **^{Nad}dA**)	TAATACGACTCACTAT **^{Nad}dA** GG	26%	5955.10	5955.78
**ODN5-2** (**X** = **^{Nad}dA**)	TAATACGACTCACT **^{Nad}dA** TAGG	29%	5955.10	5955.80
**ODN5-3** (**X** = **^{Nad}dA**)	TAATACGACTC **^{Nad}dA** CTATAGG	22%	5955.10	5956.01
**ODN5-4** (**X** = **^{Nad}dA**)	TAATACG **^{Nad}dA** CTCACTATAGG	24%	5955.10	5955.25
**ODN5-5** (**X** = **^{Nad}dA**)	TAAT **^{Nad}dA** CGACTCACTATAGG	26%	5955.10	5955.80
**ODN5-6** (**X** = **^{Nad}dA**)	TA **^{Nad}dA** TACGACTCACTATAGG	17%	5955.10	5955.08
**ODN5-7** (**X** = **^{Nad}dA**)	T **^{Nad}dA** ATACGACTCACTATAGG	14%	5955.10	5954.95
**ODN5-8** (**X** = **^{Nad}dA**)	TA **^{Nad}dA** T **^{Nad}dA** CGACTCACTATAGG	16%	6120.25	6120.59
**ODN5-8** (**X** = **^{Am2}dA**)	TA **^{Am2}dA** T **^{Am2}dA** CGACTCACTATAGG	14%	6148.27	6148.58
**ODN5-8** (**X** = **^{Bic}dA**)	TA **^{Bic}dA** T **^{Bic}dA** CGACTCACTATAGG	16%	6096.25	6096.88
**ODN5-8** (**X** = **A^{AD}**)	TA **A^{AD}** T **A^{AD}** CGACTCACTATAGG	13%	6090.23	6090.97
**ODN6**[a]	GGTAGACTACCGCTTAGAGAGTTTACGGCTCTACT<br>TCCTATCCTAACCCAAACCGTATTATTGGGCTCAC<br>CTTGTAGCCTACCTATAGTGAGTCGTATTA	–	–	–
**ODN7**[a,b]	**F**-TCTCCCTATAGTGAGTCG	–	–	–
**ODN8-native**[a]	GAAATTAATACGACTCACTATAGGGAGA	–	–	–
**ODN8** (**X** = **^{Am2}dA**)	GAAATTA **^{Am2}dA** T **^{Am2}dA** CGACTCACTATAGGGAGA	3%	9004.76	9004.28
**ODN8** (**X** = **^{Nad}dA**)	GAAATTA **^{Nad}dA** T **^{Nad}dA** CGACTCACTATAGGGAGA	4%	8976.74	8975.42
**ODN9**[a]	GGATTAGTTATTCATTACCGCCG	–	–	–

[a]Purchased from Japan Bio Services Co.,LTD.
[b]Labelled with 6-fluorescein (**F**) at 5′-terminal.
[c]Labelled with Dabcyl (**Q**) at 3′-terminal
[d]Isolated yield of the ODN after HPLC purification and desalting. The yield was deteremined by UV measurement.

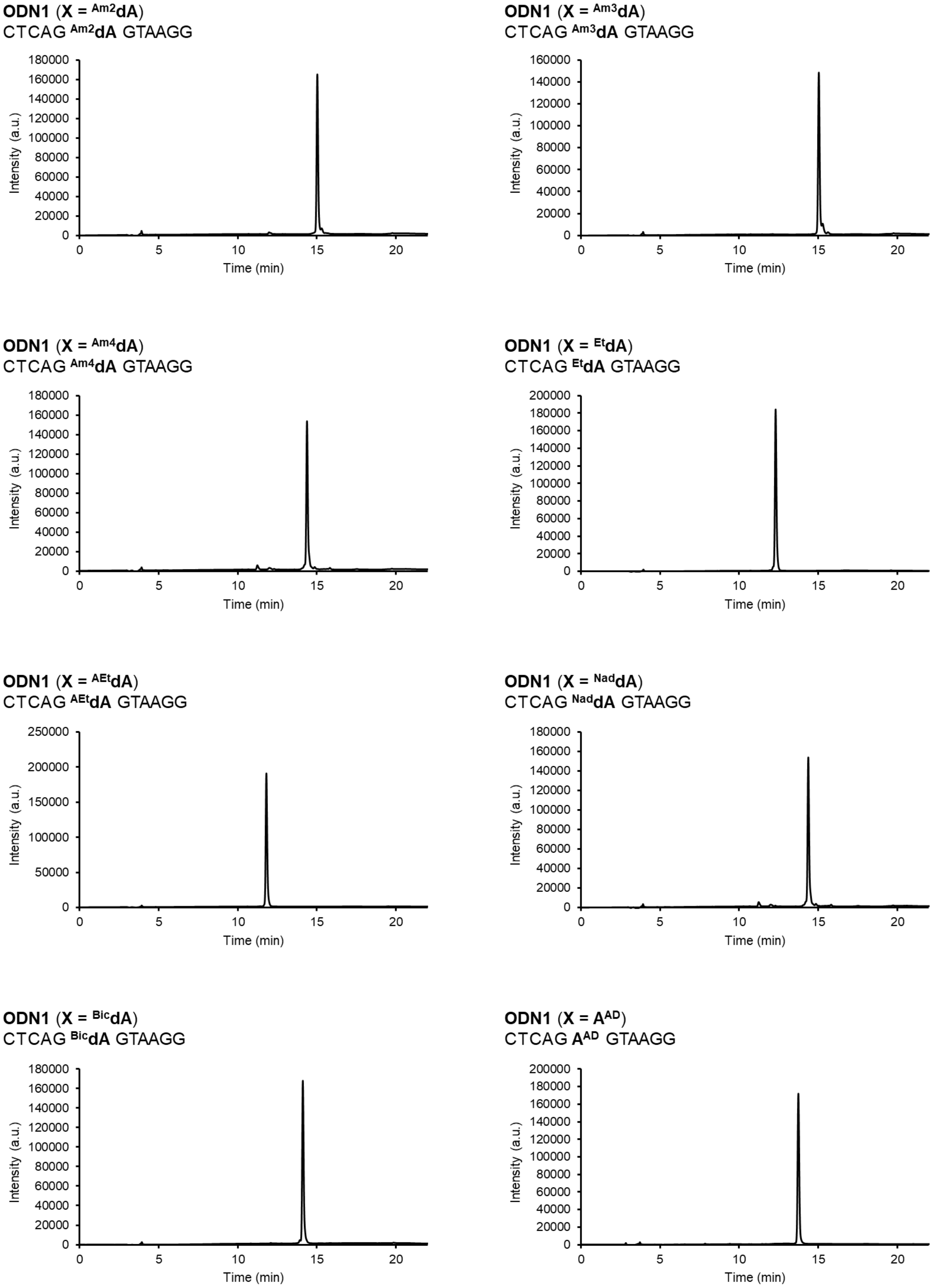

Figure S1. Analytical HPLC traces of the purified ODNs.

ODN3 (**X** = ^{Am2}**dA**)
FAM-CTCAG ^{Am2}**dA** GTAAGG

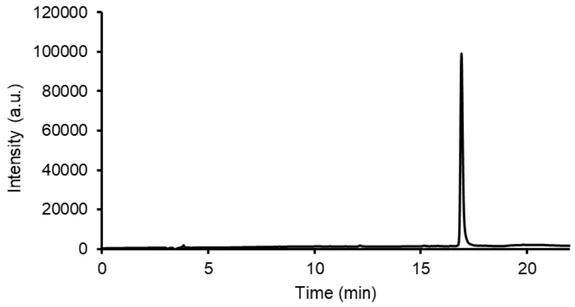

ODN3 (**X** = ^{Nad}**dA**)
FAM-CTCAG ^{Nad}**dA** GTAAGG

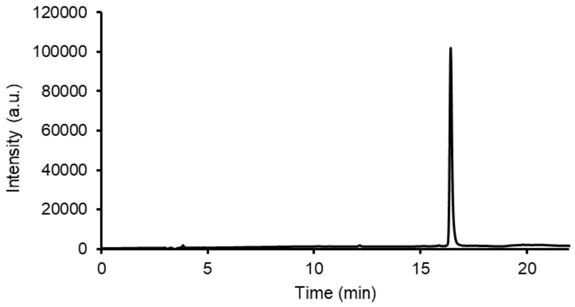

ODN3 (**X** = ^{Bic}**dA**)
FAM-CTCAG ^{Bic}**dA** GTAAGG

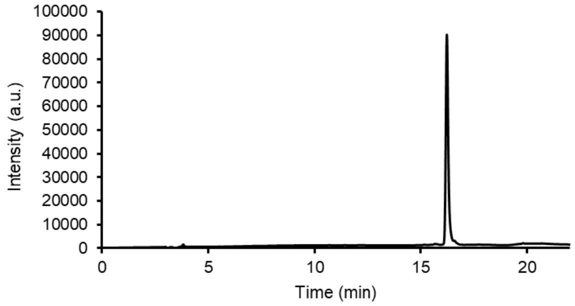

ODN5-1 (**X** = ^{Nad}**dA**)
TAATACGACTCACTAT ^{Nad}**dA** GG

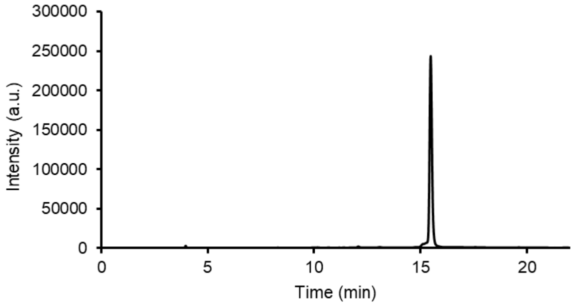

ODN5-2 (**X** = ^{Nad}**dA**)
TAATACGACTCACT ^{Nad}**dA** TAGG

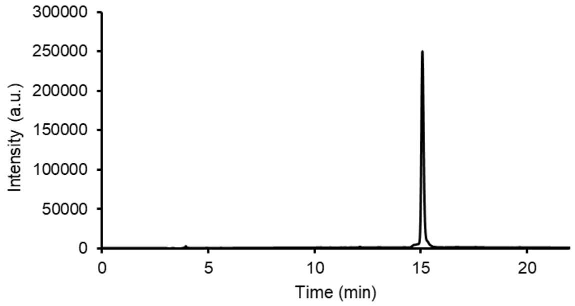

ODN5-3 (**X** = ^{Nad}**dA**)
TAATACGACTC ^{Nad}**dA** CTATAGG

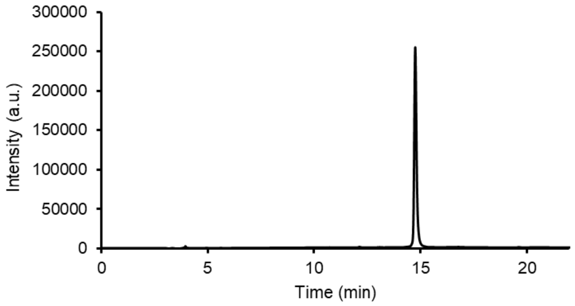

ODN5-4 (**X** = ^{Nad}**dA**)
TAATACG ^{Nad}**dA** CTCACTATAGG

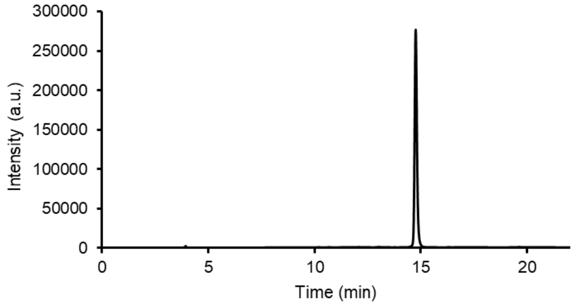

ODN5-5 (**X** = ^{Nad}**dA**)
TAAT ^{Nad}**dA** CGACTCACTATAGG

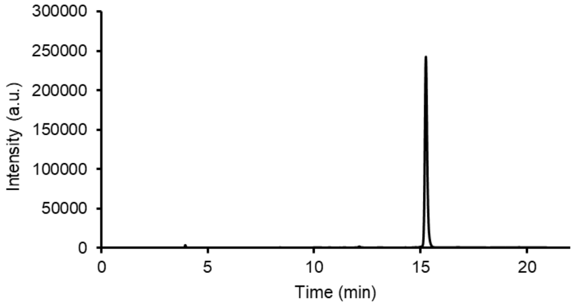

Figure S1 (continued). Analytical HPLC traces of the purified ODNs.

ODN5-6 (**X** = ^{Nad}**dA**)
TA ^{Nad}**dA** TACGACTCACTATAGG

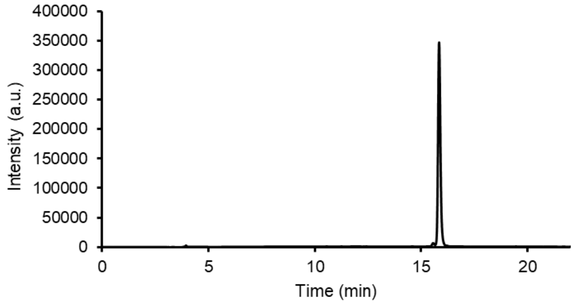

ODN5-7 (**X** = ^{Nad}**dA**)
T ^{Nad}**dA** ATACGACTCACTATAGG

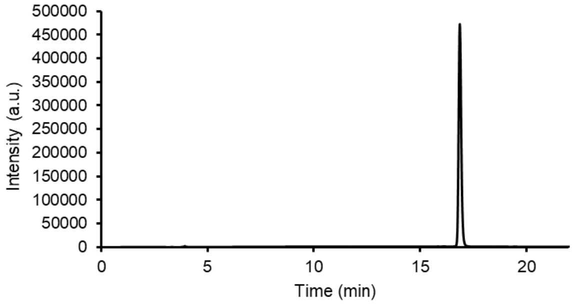

ODN5-1 (**X** = ^{Nad}**dA**)
TAATACGACTCACTAT ^{Nad}**dA** GG

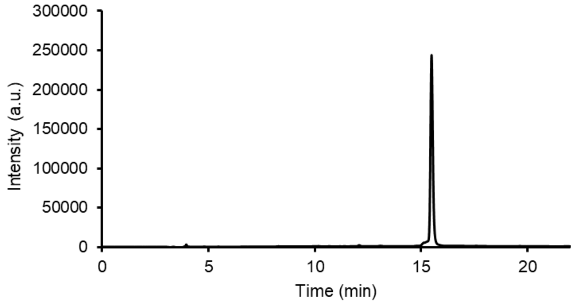

ODN5-2 (**X** = ^{Nad}**dA**)
TAATACGACTCACT ^{Nad}**dA** TAGG

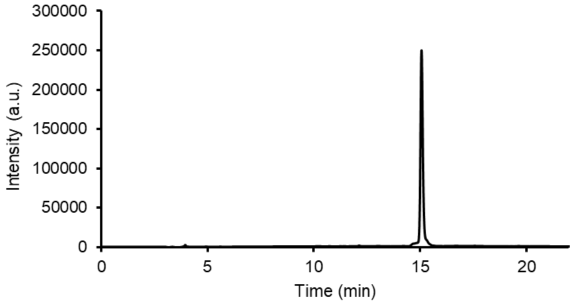

ODN5-3 (**X** = ^{Nad}**dA**)
TAATACGACTC ^{Nad}**dA** CTATAGG

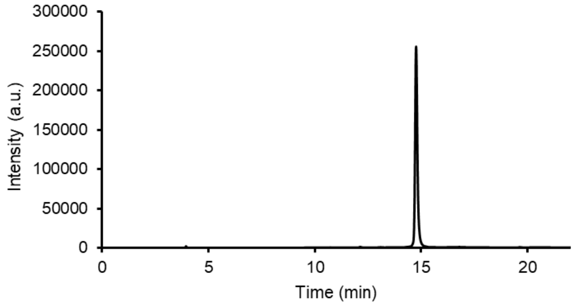

ODN5-4 (**X** = ^{Nad}**dA**)
TAATACG ^{Nad}**dA** CTCACTATAGG

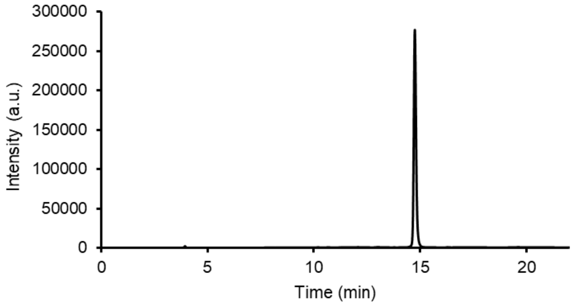

ODN5-5 (**X** = ^{Nad}**dA**)
TAAT ^{Nad}**dA** CGACTCACTATAGG

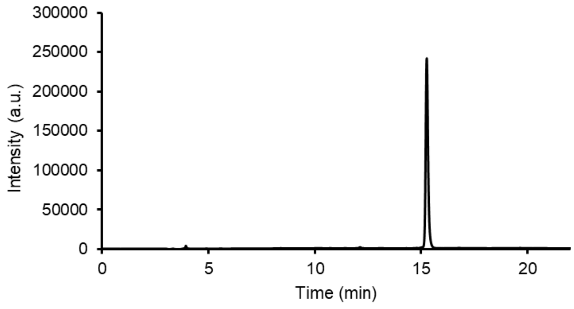

ODN5-6 (**X** = ^{Nad}**dA**)
TA ^{Nad}**dA** TACGACTCACTATAGG

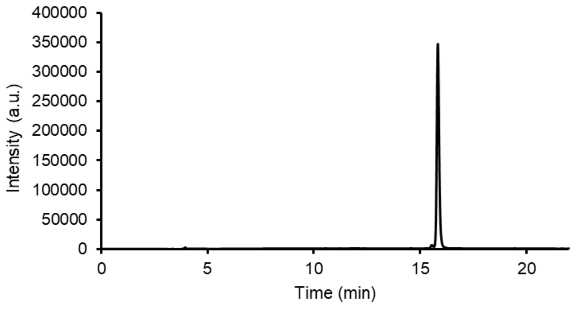

Figure S1 (continued). Analytical HPLC traces of the purified ODNs.

ODN5-7 (**X = ^{Nad}dA**)
T ^{Nad}**dA** ATACGACTCACTATAGG

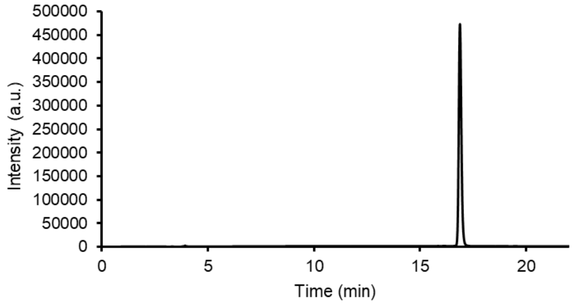

ODN5-8 (**X = ^{Nad}dA**)
TA ^{Nad}**dA** T ^{Nad}**dA** CGACTCACTATAGG

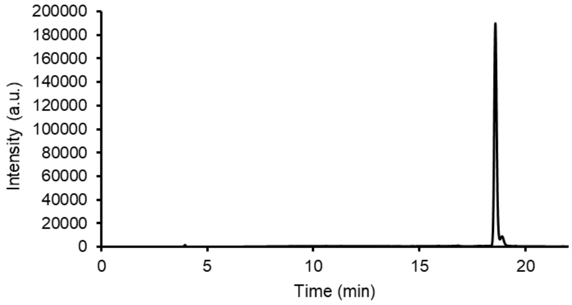

ODN5-8 (**X = ^{Am2}dA**)
TA ^{Am2}**dA** T ^{Am2}**dA** CGACTCACTATAGG

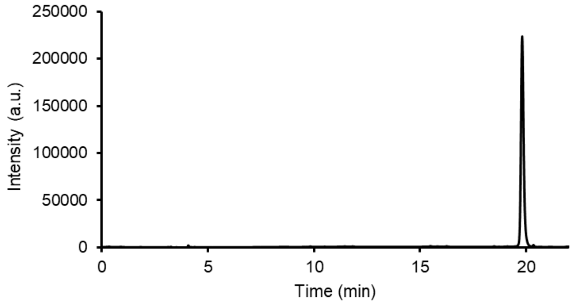

ODN5-8 (**X = ^{Bic}dA**)
TA ^{Bic}**dA** T ^{Bic}**dA** CGACTCACTATAGG

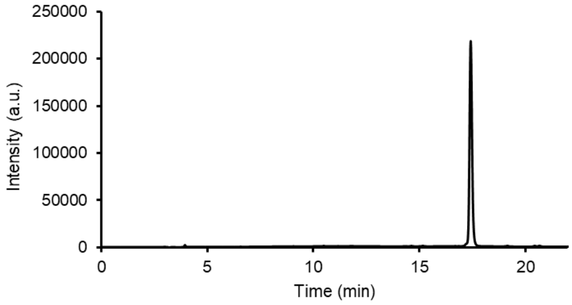

ODN5-8 (**X = A^{AD}**)
TA **A^{AD}** T **A^{AD}** CGACTCACTATAGG

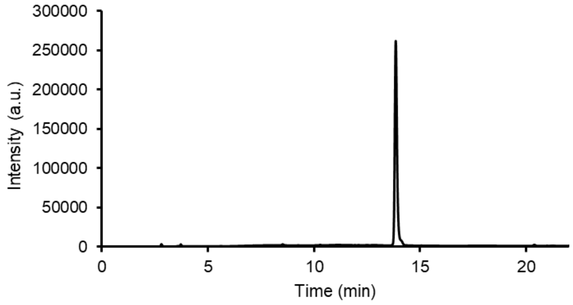

ODN8 (**X = ^{Am2}dA**)
GAAATTA ^{Am2}**dA** T ^{Am2}**dA** CGACTCACTATAGGGAGA

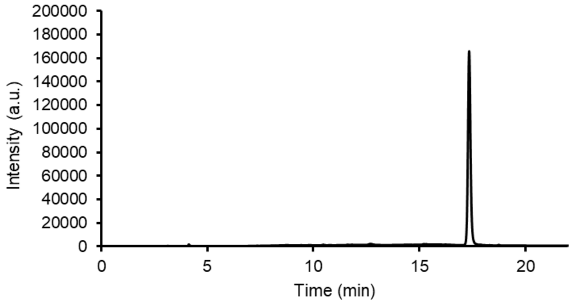

ODN8 (**X = ^{Nad}dA**)
GAAATTA ^{Nad}**dA** T ^{Nad}**dA** CGACTCACTATAGGGAGA

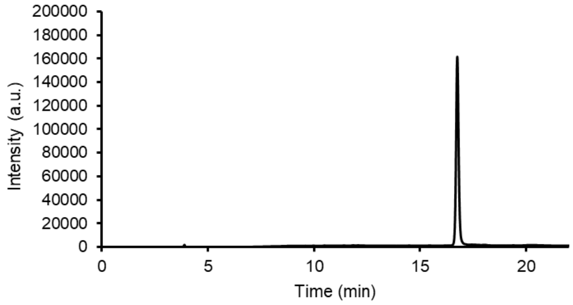

Figure S1 (continued). Analytical HPLC traces of the purified ODNs.

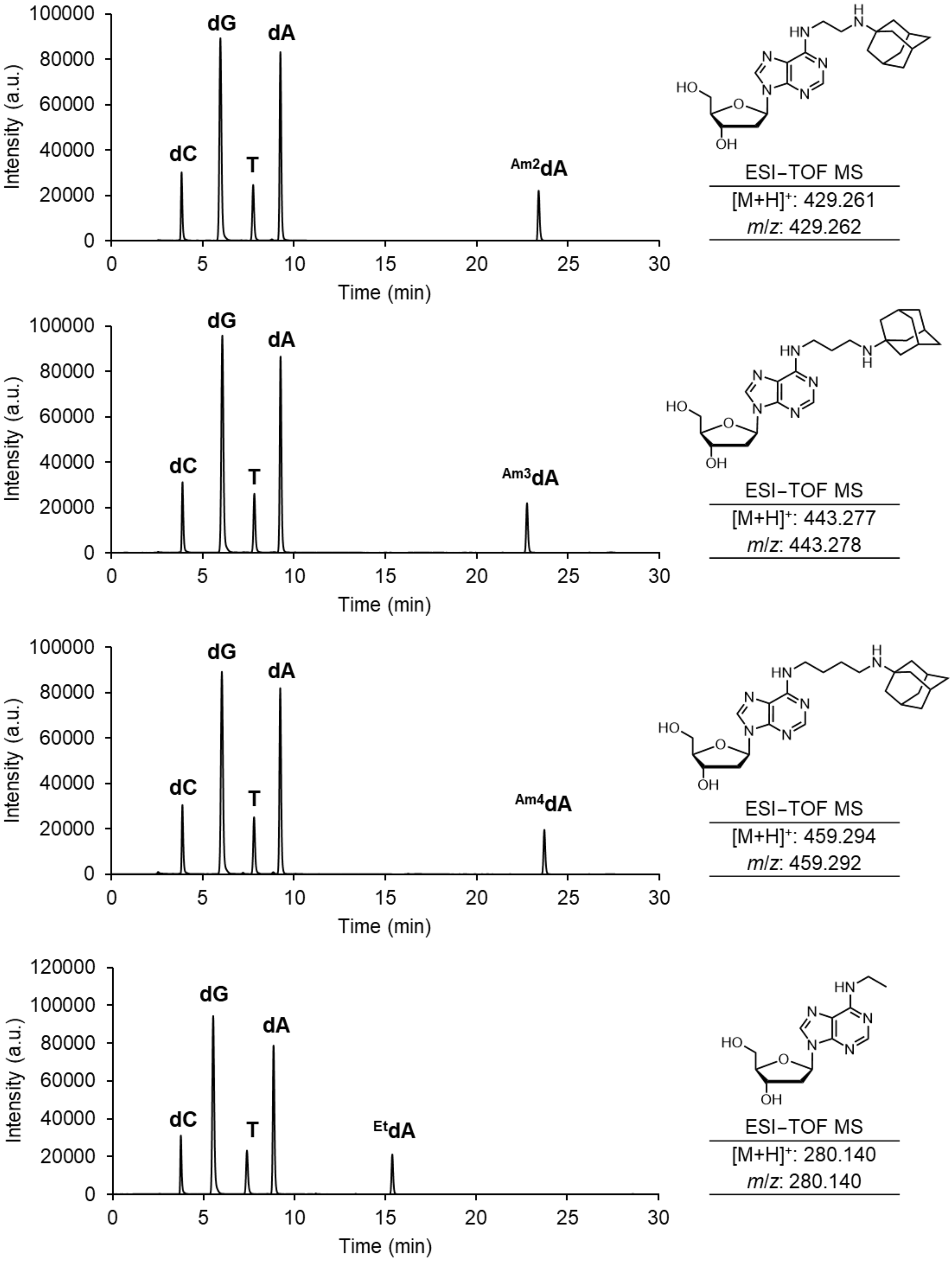

Figure S2. Analytical HPLC traces of the digested ODNs.

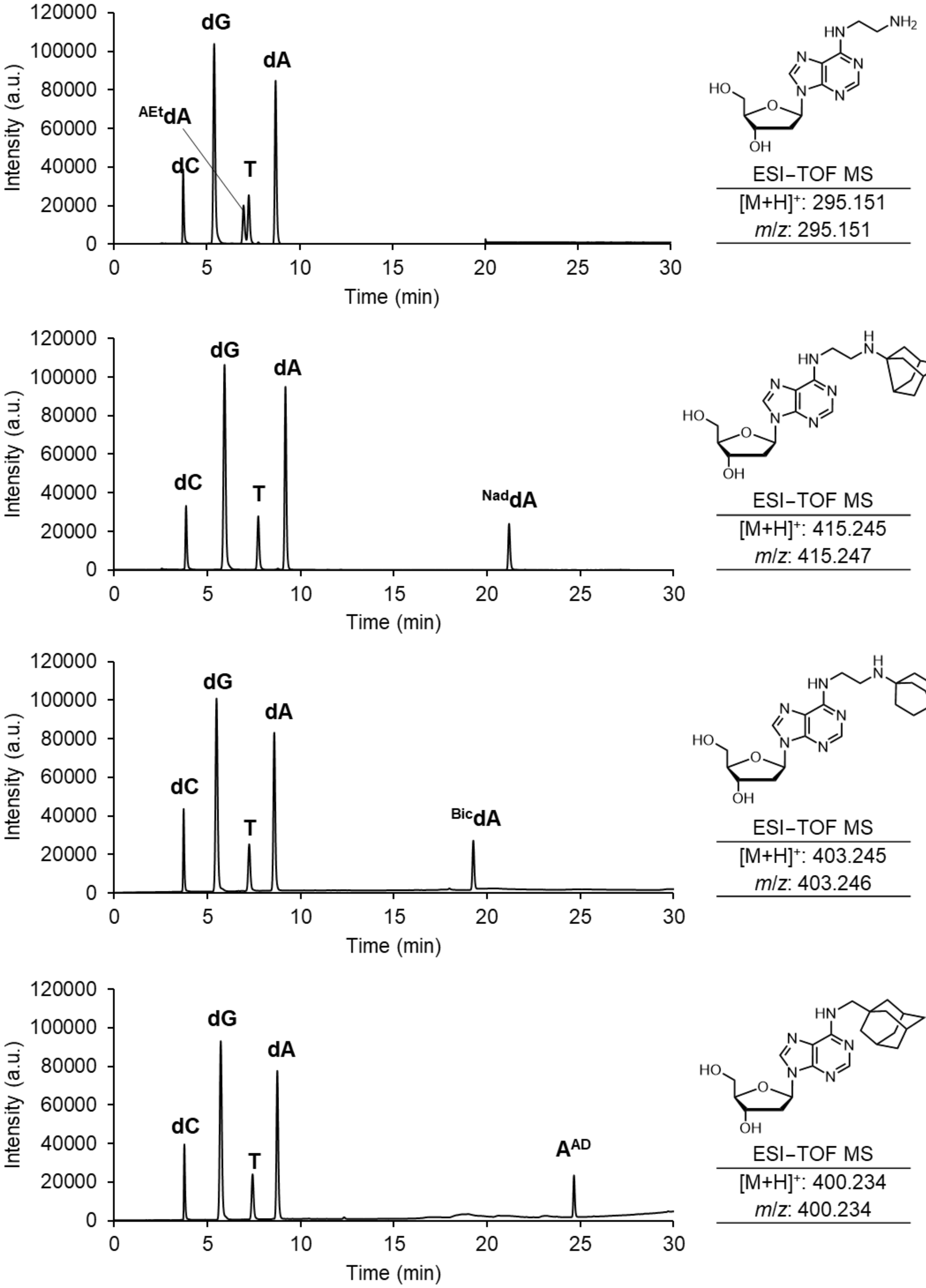

Figure S2 (continued). Analytical HPLC traces of the digested ODNs.

4. Base pairing properties of ^{Am2}dA, ^{Am3}dA, and ^{Am4}dA

ODN1: 5′ CTCAG **X** GTAAGG 3′
ODN2: 3′ GAGTC **Y** CATTCC 5′

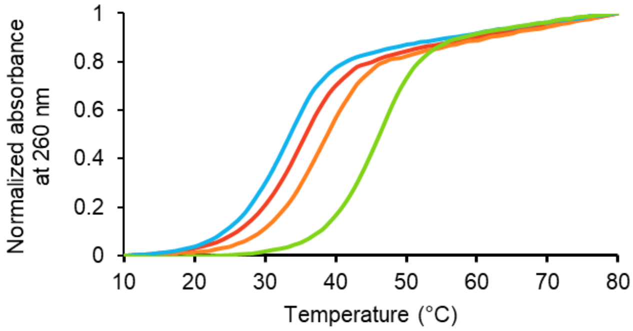

X	Y	T_m (°C)	S.E.
**A**	**A**	34.9	±0.02
**A**	**G**	37.7	±0.14
**A**	**C**	32.9	±0.07
**A**	**T**	45.5	±0.11

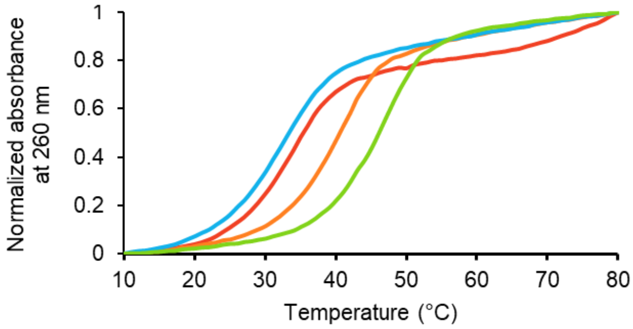

X	Y	T_m (°C)	S.E.
**^{Am2}dA**	**A**	33.6	±0.08
**^{Am2}dA**	**G**	40.1	±0.21
**^{Am2}dA**	**C**	32.2	±0.02
**^{Am2}dA**	**T**	47.1	±0.09

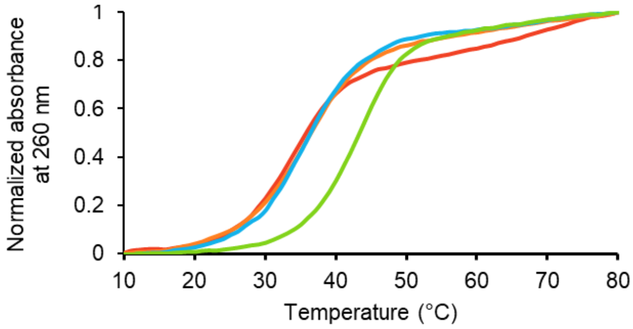

X	Y	T_m (°C)	S.E.
**^{Am3}dA**	**A**	33.5	±0.14
**^{Am3}dA**	**G**	35.9	±0.10
**^{Am3}dA**	**C**	35.5	±0.18
**^{Am3}dA**	**T**	43.3	±0.04

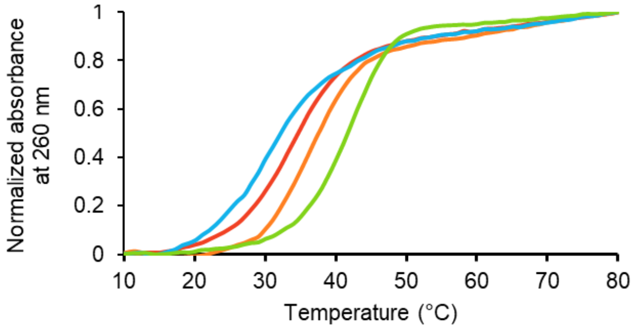

X	Y	T_m (°C)	S.E.
**^{Am4}dA**	**A**	34.4	±0.34
**^{Am4}dA**	**G**	36.3	±0.09
**^{Am4}dA**	**C**	32.7	±1.02
**^{Am4}dA**	**T**	42.5	±0.02

Figure S3. UV melting curves and T_m values of the DNA duplexes containing **A**, **^{Am2}dA**, **^{Am3}dA**, and **^{Am4}dA** at position **X** and each canonical nucleoside at position **Y**. Each T_m value is average of three measurements and provided with standard errors (S.E.).

5. Base pairing properties of ^{Et}dA and ^{AEt}dA

(a)

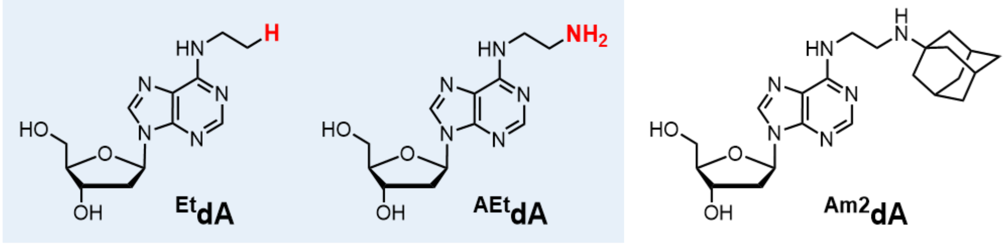

(b)

ODN1: 5′ CTCAG **X** GTAAGG 3′
ODN2: 3′ GAGTC **Y** CATTCC 5′

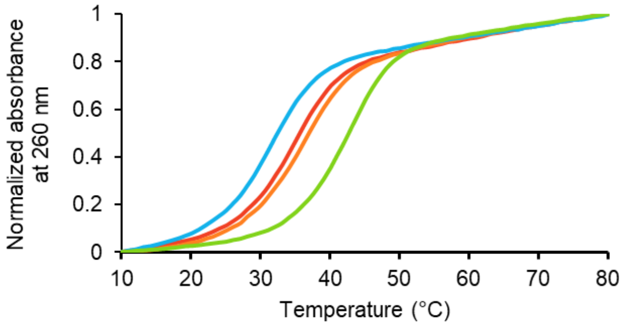

	X	Y	T_m (°C)	S.E.
red	^{Et}**dA**	**A**	34.9	±0.02
orange	^{Et}**dA**	**G**	36.1	±0.04
blue	^{Et}**dA**	**C**	31.6	±0.04
green	^{Et}**dA**	**T**	42.7	±0.02

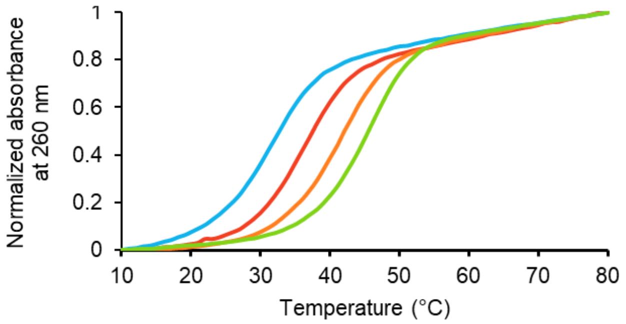

	X	Y	T_m (°C)	S.E.
red	^{AEt}**dA**	**A**	36.5	±0.04
orange	^{AEt}**dA**	**G**	41.6	±0.05
blue	^{AEt}**dA**	**C**	31.8	±0.07
green	^{AEt}**dA**	**T**	45.7	±0.02

(c)

**X-Y**	**A-T**	^{Et}**dA-T**	^{AEt}**dA-T**	^{Am2}**dA-T**
T_m (°C)	45.5	42.7	45.7	47.1

Figure S4. (a) Structures of ^{Et}**dA** and ^{Eda}**dA** for investigating the recognition mode of ^{Am2}**dA-T** pair. (b) UV melting curves and T_m values of the DNA duplexes containing ^{Et}**dA** and ^{AEt}**dA** at position **X** and each canonical nucleoside at position **Y**. Each T_m value is average of three measurements and provided with standard errors (S.E.). (c) Comparison of the T_m values of the DNA duplexes (**ODN1**/**ODN2**) containing **A-T**, ^{Et}**dA-T**, ^{AEt}**dA-T**, and ^{Am2}**dA-T** pairs at position **X-Y**.

6. Reversibility and kinetic analyses of the duplex formation (^{Am2}dA, ^{Am3}dA, ^{Am4}dA)

ODN1: 5′ CTCAG **A** GTAAGG 3′
ODN2: 3′ GAGTC **T** CATTCC 5′

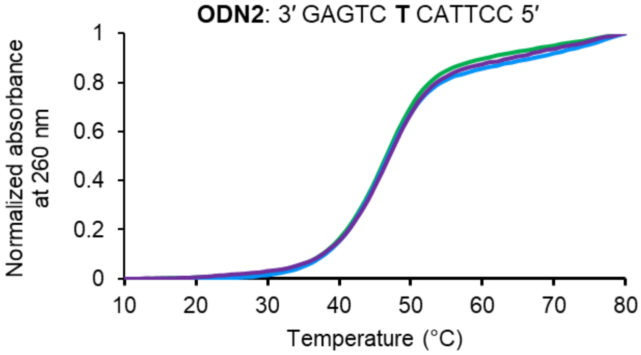

	Additives	T_m (°C)	S.E.
	None	46.0	±0.04
	+ CB[7]	45.9	±0.05
	+ CB[7] then + Ad^{Eda}	46.4	±0.05

ODN1: 5′ CTCAG **^{Am2}dA** GTAAGG 3′
ODN2: 3′ GAGTC **T** CATTCC 5′

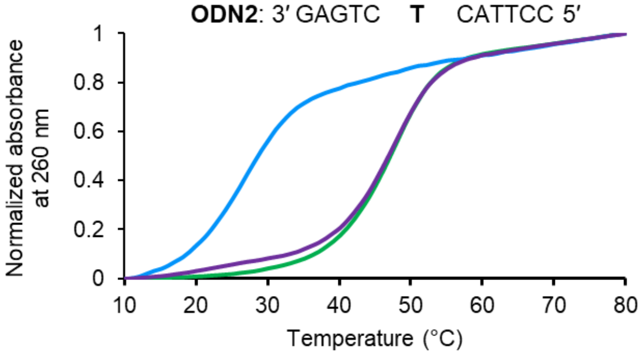

	Additives	T_m (°C)	S.E.
	None	47.1	±0.09
	+ CB[7]	26.7	±0.02
	+ CB[7] then + Ad^{Eda}	47.1	±0.04

ODN1: 5′ CTCAG **^{Am3}dA** GTAAGG 3′
ODN2: 3′ GAGTC **T** CATTCC 5′

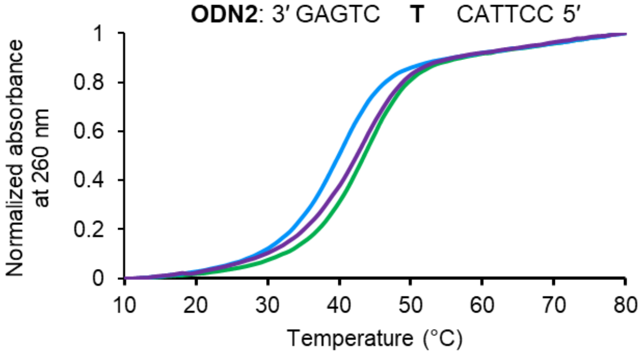

	Additives	T_m (°C)	S.E.
	None	43.3	±0.04
	+ CB[7]	39.7	±0.03
	+ CB[7] then + Ad^{Eda}	43.3	±0.20

ODN1: 5′ CTCAG **^{Am4}dA** GTAAGG 3′
ODN2: 3′ GAGTC **T** CATTCC 5′

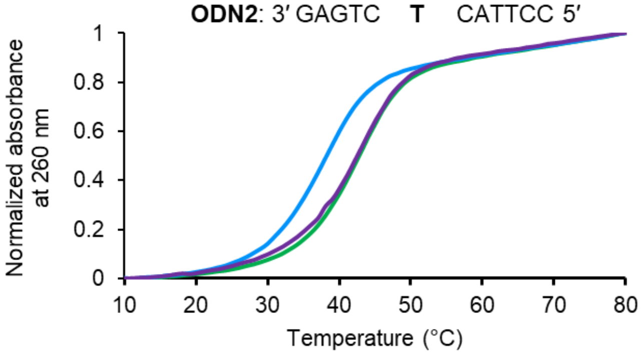

	Additives	T_m (°C)	S.E.
	None	42.5	±0.02
	+ CB[7]	37.8	±0.22
	+ CB[7] then + Ad^{Eda}	42.6	±0.06

Figure S5. UV melting curves and summary of T_m values of the DNA duplexes containing **A**-**T**, **^{Am2}dA**-**T**, **^{Am3}dA**-**T** and **^{Am4}dA**-**T** pairs upon alternating treatment with CB[7] and Ad^{Eda}.

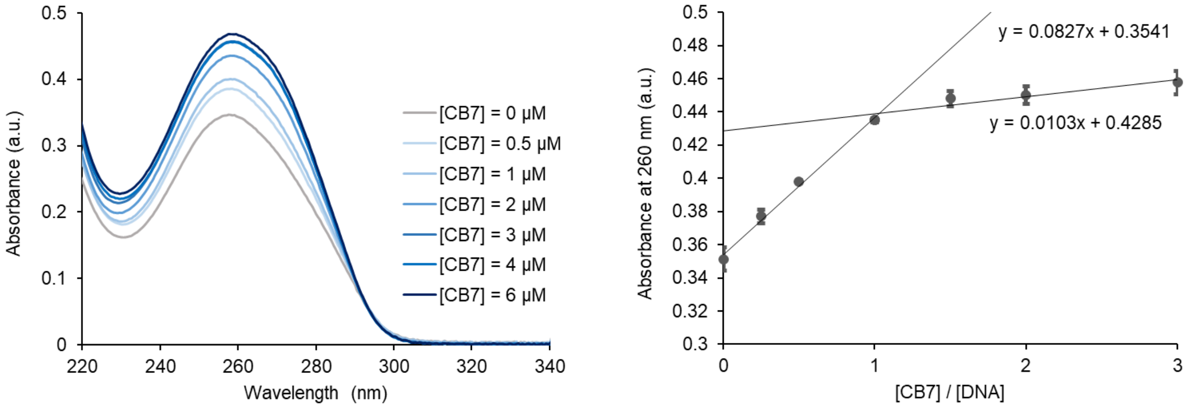

Figure S6. (a) UV absorption spectra of DNA duplex containing **^{Am2}dA-T** pair in the presence of different concentrations of CB[7]. Conditions: 2 μM of each ODN, 150 mM NaCl, 10 mM sodium phosphate buffer at 37 °C. (b) Plot of absorbance at 260 nm versus the ratio of CB[7] and DNA (i.e., [CB7]/[DNA]).

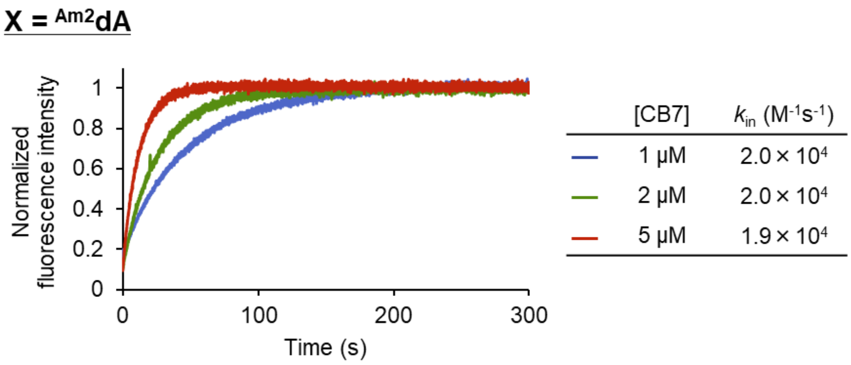

Figure S7. The time course change of fluorescence obtained from the DNA duplex containing **^{Am2}dA** in the presence of different concentration of CB[7]. The signal change was monitored by stopped-flow measurement (λ_{ex} = 495 nm and λ_{em} > 525 nm). The kinetic parameters were determined by a non-linear least squares regression analysis of the respective curve.

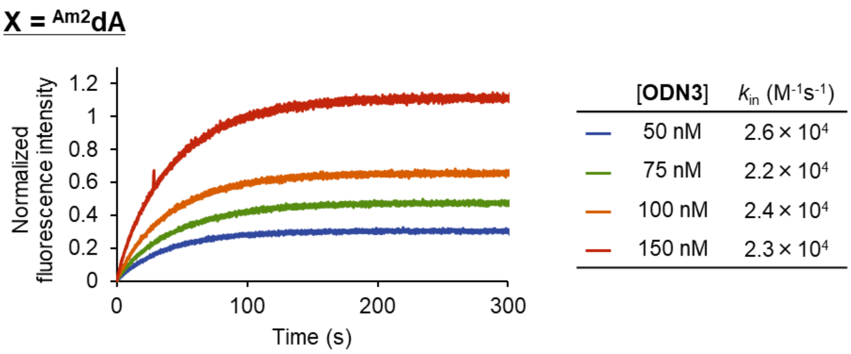

Figure S8. The time course change of fluorescence obtained by using different concentrations of the DNA duplex containing **^{Am2}dA**. 1 μM CB[7] was added and the signal change was monitored by stopped-flow measurement (λ_{ex} = 495 nm and λ_{em} > 525 nm). The kinetic parameters were determined by a non-linear least squares regression analysis of the respective curve.

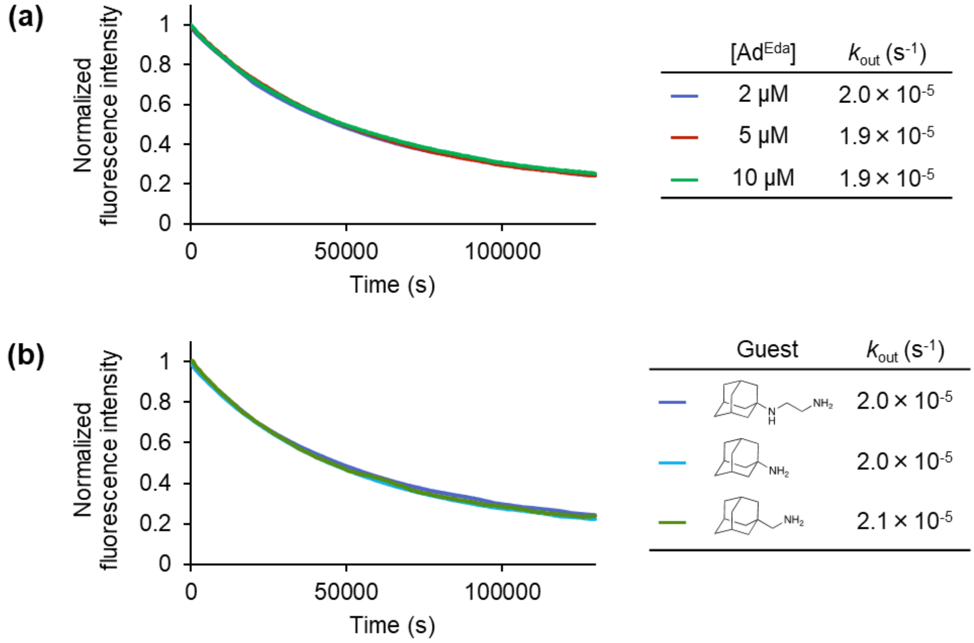

Figure S9. The time course change of fluorescence obtained from the guest exchange reaction of the DNA containing **^{Am2}dA** complexed with CB[7]. (a) The guest exchange reaction in the presence of different concentrations of Ad^{Eda}. (b) The guest exchange reaction using different exchanging guest (2 μM). The signal change was monitored by fluoresncece measurement (λ_{ex} = 495 nm and λ_{em} = 520 nm). The kinetic parameters were determined by a non-linear least squares regression analysis of the respective curve.

7. Reversibility and kinetic analyses of the duplex formation (^{Nad}dA, ^{Bic}dA)

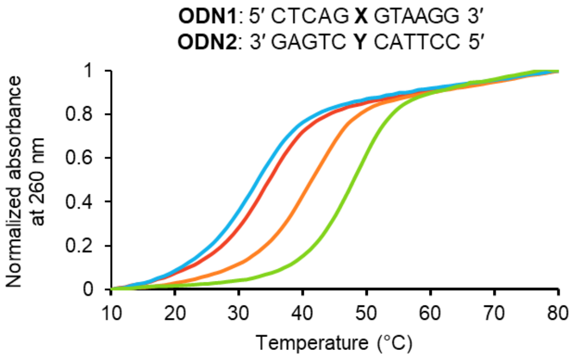

	X	Y	T_m (°C)	S.E.
—	^{Nad}**dA**	**A**	34.0	±0.03
—	^{Nad}**dA**	**G**	41.2	±0.01
—	^{Nad}**dA**	**C**	32.2	±0.09
—	^{Nad}**dA**	**T**	47.9	±0.03

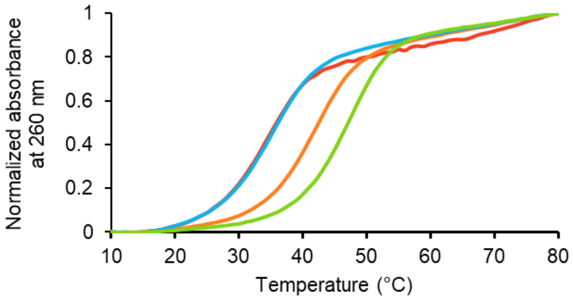

	X	Y	T_m (°C)	S.E.
—	^{Bic}**dA**	**A**	34.3	±0.04
—	^{Bic}**dA**	**G**	41.9	±0.08
—	^{Bic}**dA**	**C**	35.1	±0.02
—	^{Bic}**dA**	**T**	47.2	±0.02

Figure S10. UV melting curves and summary of T_m values of the DNA duplexes containing ^{Nad}**dA** and ^{Bic}**dA** at position **X** and each canonical nucleoside at position **Y**.

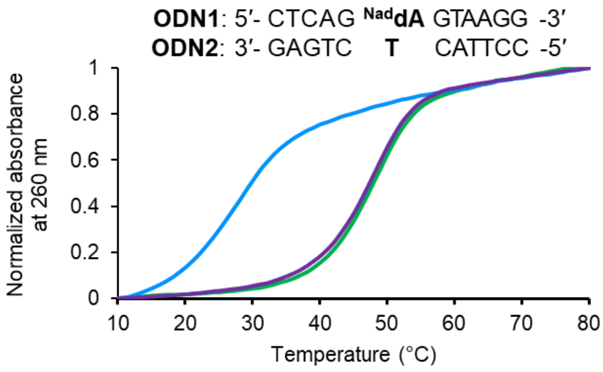

	Additives	T_m (°C)	S.E.
—	None	47.9	±0.03
—	+ CB[7]	27.7	±0.08
—	+ CB[7] then + Ad^{Eda}	47.7	±0.02

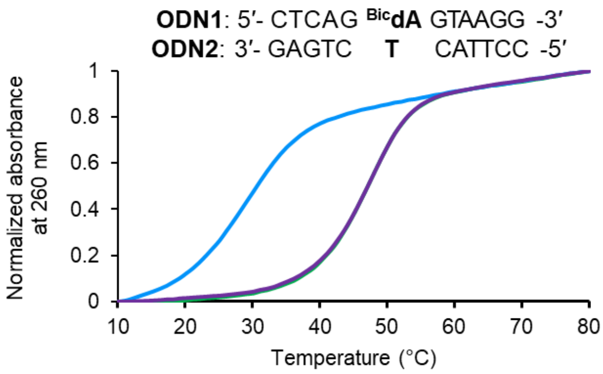

	Additives	T_m (°C)	S.E.
—	None	47.2	±0.02
—	+ CB[7]	29.2	±0.07
—	+ CB[7] then + Ad^{Eda}	47.1	±0.06

Figure S11. UV melting curves and summary of T_m values of the DNA duplexes containing ^{Nad}**dA-T** and ^{Bic}**dA-T** pairs upon alternating treatment with CB[7] and Ad^{Eda}.

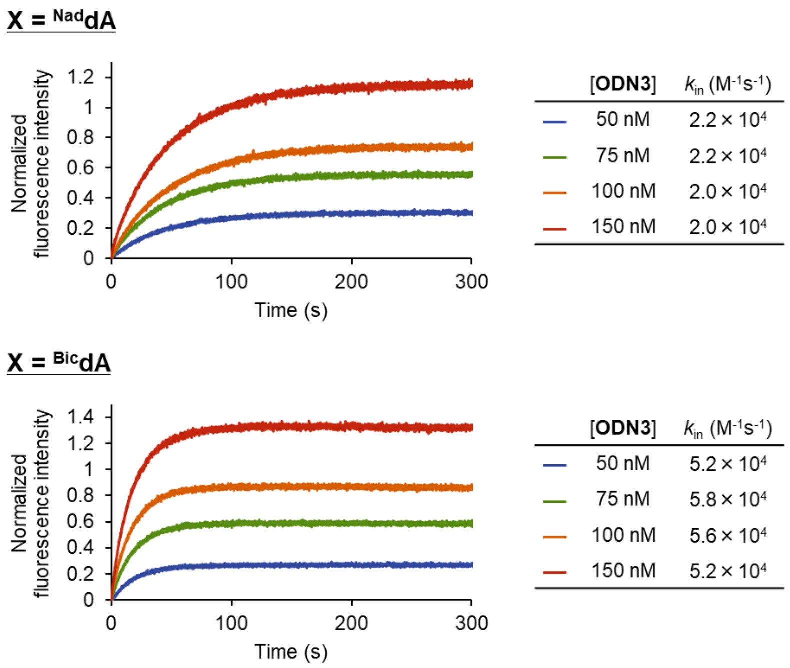

Figure S12. The time course change of fluorescence obtained by using different concentration of DNA duplex containing **^{Nad}dA** and **^{Bic}dA**. 1 μM CB[7] was added and the signal change was monitored by stopped-flow measurement (λ_{ex} = 495 nm and λ_{em} > 525 nm). The kinetic parameters were determined by a non-linear least squares regression analysis of the respective curve.

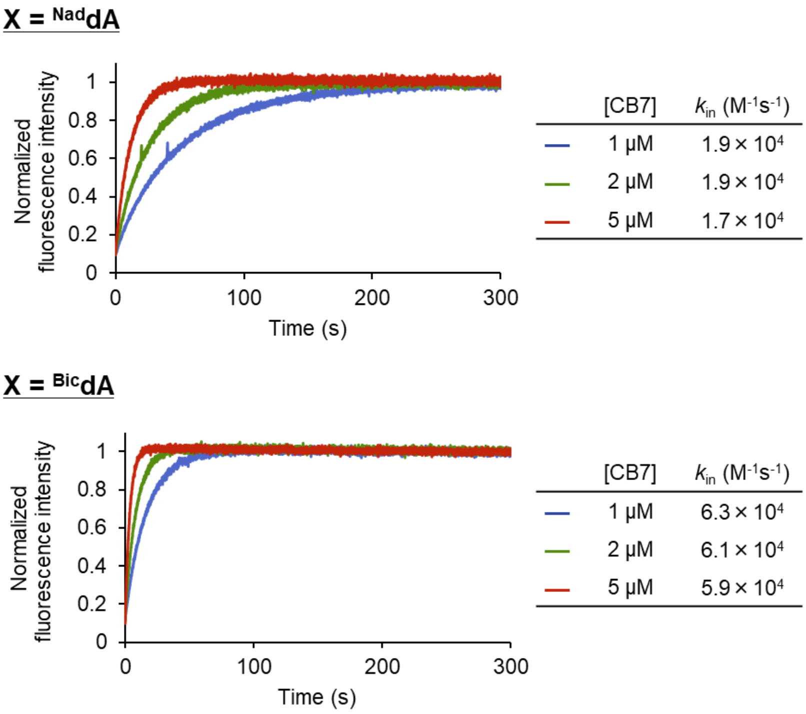

Figure S13. The time course change of fluorescence obtained from the DNA duplex containing **^{Nad}dA** and **^{Bic}dA** in the presence of different concentration of CB[7]. The signal change was monitored by stopped-flow measurement (λ_{ex} = 495 nm and λ_{em} > 525 nm). The kinetic parameters were determined by a non-linear least squares regression analysis of the respective curve.

8. Reversibility and kinetic analyses of the duplex formation (A^{AD})

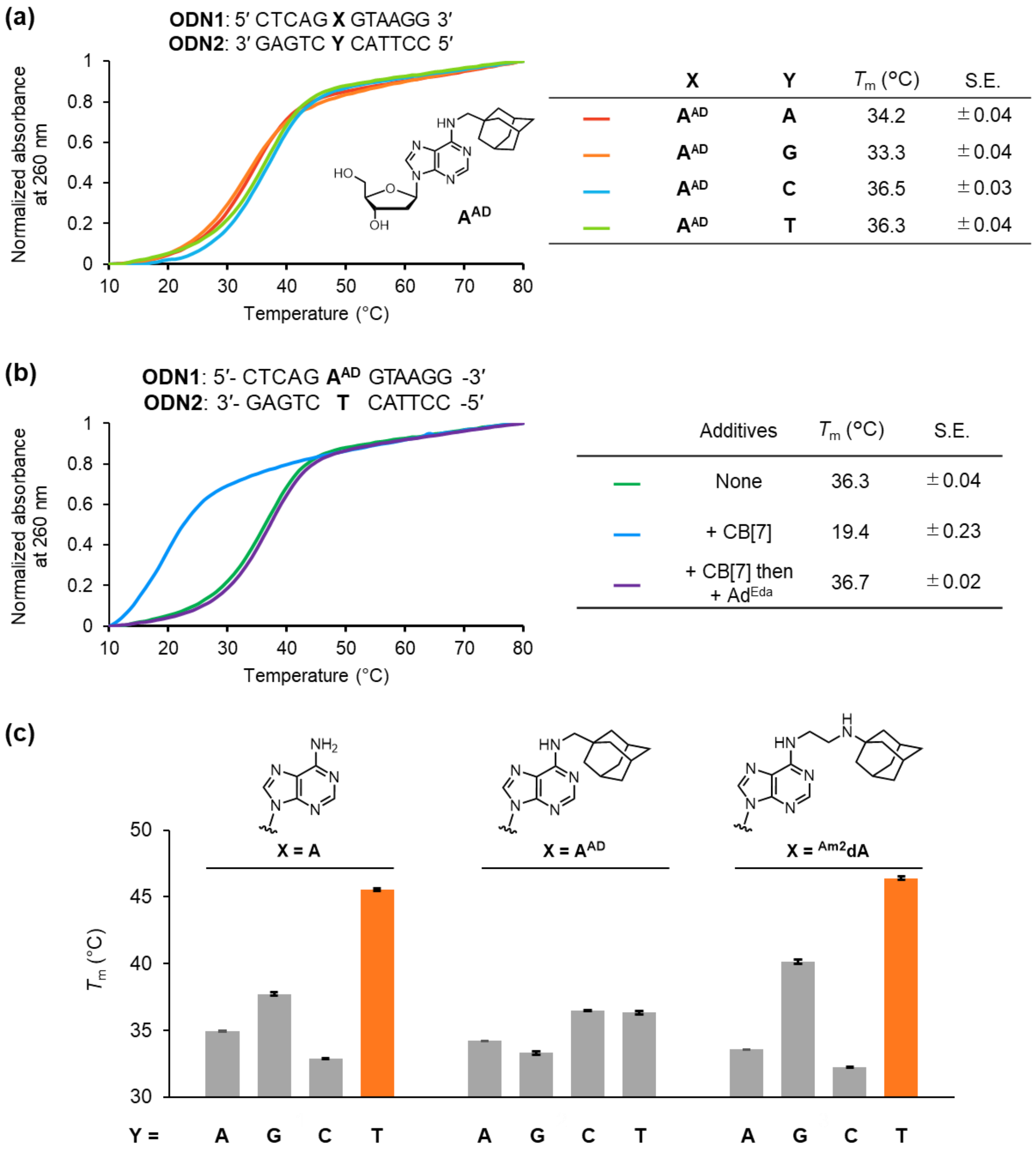

Figure S14. Base pairing properties of $\mathbf{A^{AD}}$ nucleoside reported by Xiao *et al.* (a) UV melting curves and summary of the T_m values of the DNA duplexes containing $\mathbf{A^{AD}}$ at position **X** and each canonical nucleoside at position **Y**. (b) UV melting curves of the DNA duplexes containing $\mathbf{A^{AD}}$-**T** pair upon alternating treatment with CB[7] and Ad^{Eda}. (c) Comparison of the base pairing properties of **A**, $\mathbf{^{Am2}dA}$ and $\mathbf{A^{AD}}$ in DNA duplexes.

9. In vitro transcription of SQ-DNA incorporating the guest-modified adenosines

```
-17                      -1
taatacgactcactataGGTAGGCTACAAGGTGAGCCCAATAATACGGTTTGGGTTAGGA
      T7 promoter
TAGGAAGTAGAGCCGTAAACTCTCTAAGCGGTAGTCTACC
```

Figure S15. The nucleotide sequence of **SQ-DNA** used for the promoter activity assays (100 bps, only the non-template sequence is shown). The sequence encoding Squash aptamer is shown by the capital letter and the others by small letter.The underlined region is the T7 promoter sequence.

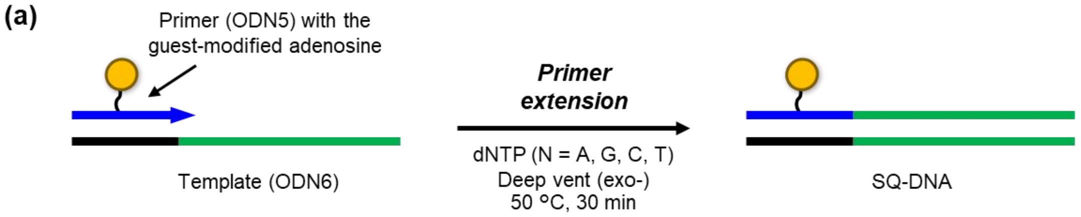

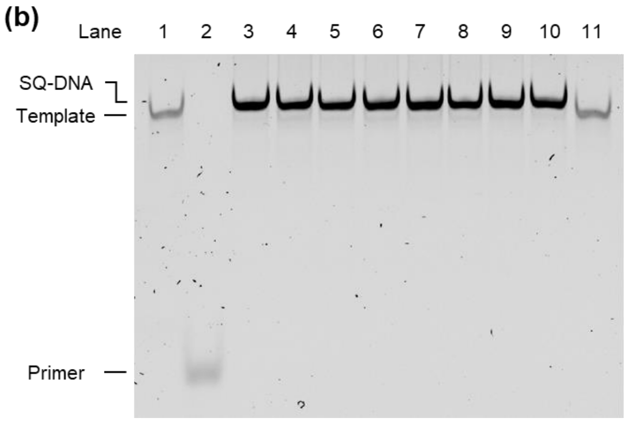

SqDNA	Modified position in the promoter
**SQ-DNA-native**	TAATACGACTCACTATA
**SQ-DNA-1 (X = ^{Nad}dA)**	TAATACGACTCACTAT**X**
**SQ-DNA-2 (X = ^{Nad}dA)**	TAATACGACTCACT**X**TA
**SQ-DNA-3 (X = ^{Nad}dA)**	TAATACGACTC**X**CTATA
**SQ-DNA-4 (X = ^{Nad}dA)**	TAATACG**X**CTCACTATA
**SQ-DNA-5 (X = ^{Nad}dA)**	TAAT**X**CGACTCACTATA
**SQ-DNA-6 (X = ^{Nad}dA)**	TA**X**TACGACTCACTATA
**SQ-DNA-7 (X = ^{Nad}dA)**	T**XX**TACGACTCACTATA

Figure S16. (a) A schematic illustration of the enzymatic synthesis of **SQ-DNA** by primer extension reaction. **ODN5** (primer) was elongated against **ODN6** (template) by Deep Vent (exo-) polymerase in the presence of dNTP. (b) Native PAGE analysis of **SQ-DNA** after spin column purification. Lane 1 and 11: **ODN6** (template). Lane 2: **ODN5-native** (primer). Lane 3: **SQ-DNA-native**. Lane 4: **SQ-DNA-1** (**X** = ^{Nad}**dA**). Lane 5: **SQ-DNA-2** (**X** = ^{Nad}**dA**). Lane 6: **SQ-DNA-3** (**X** = ^{Nad}**dA**). Lane 7: **SQ-DNA-4** (**X** = ^{Nad}**dA**). Lane 8: **SQ-DNA-5** (**X** = ^{Nad}**dA**). Lane 9: **SQ-DNA-6** (**X** = ^{Nad}**dA**). Lane 10: **SQ-DNA-7** (**X** = ^{Nad}**dA**). The DNA bands were stained and visualized using SYBR Gold Nucleic Acid Gel Stain.

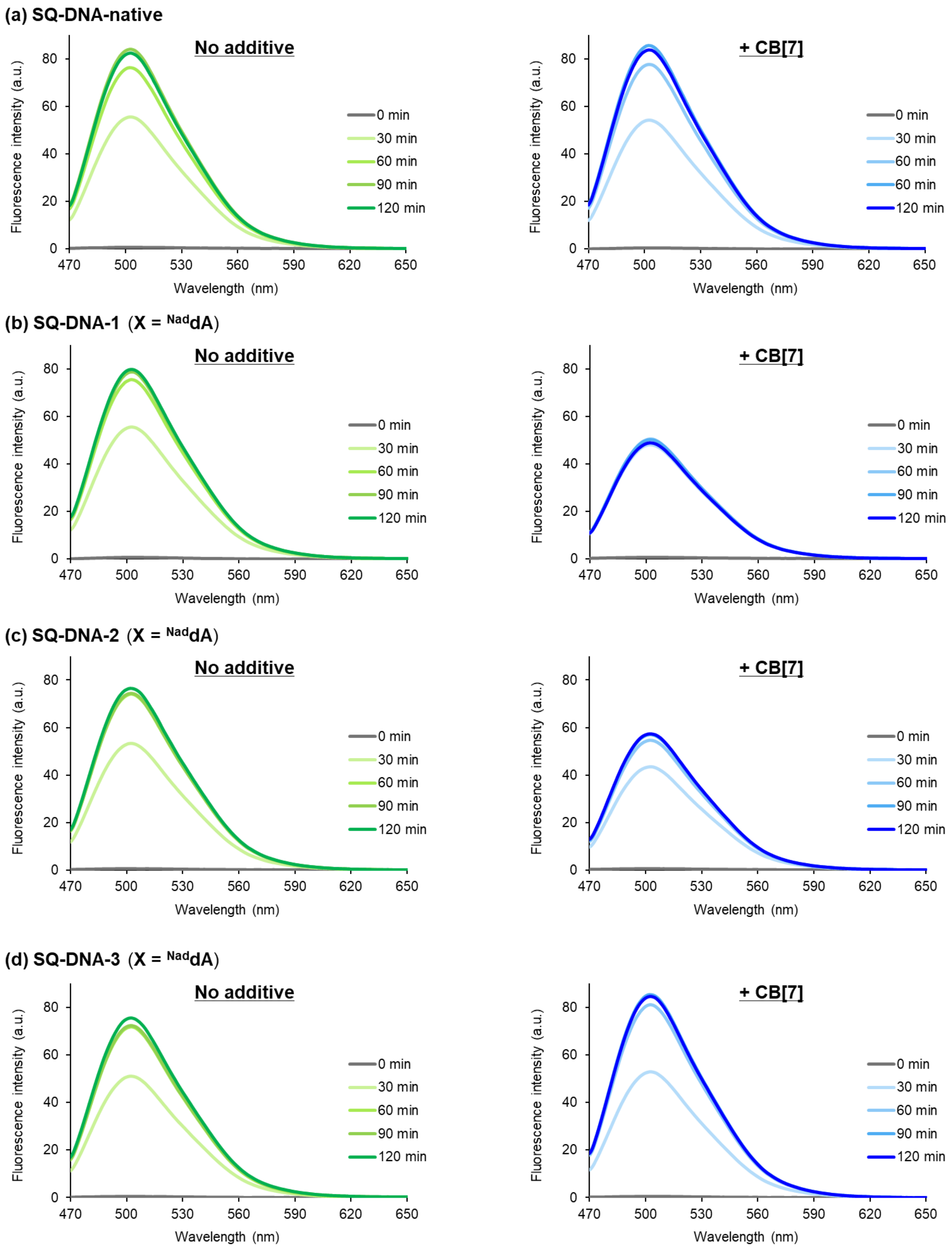

Figure S17. Fluorescence spectra obtained from the transcription reaction of each **SQ-DNA** in the absence and presence of CB[7]. The spectra were measured using aliquots of the transcription mixture sampled at different time points (0, 30, 60, 90, 120 min) in the presence of DFHBI-1T. (a) **SQ-DNA-native**. (b) **SQ-DNA-1** (**X** = ^{Nad}**dA**). (c) **SQ-DNA-2** (**X** = ^{Nad}**dA**). (d) **SQ-DNA-3** (**X** = ^{Nad}**dA**).

(e) SQ-DNA-4 (**X** = ^{Nad}**dA**)

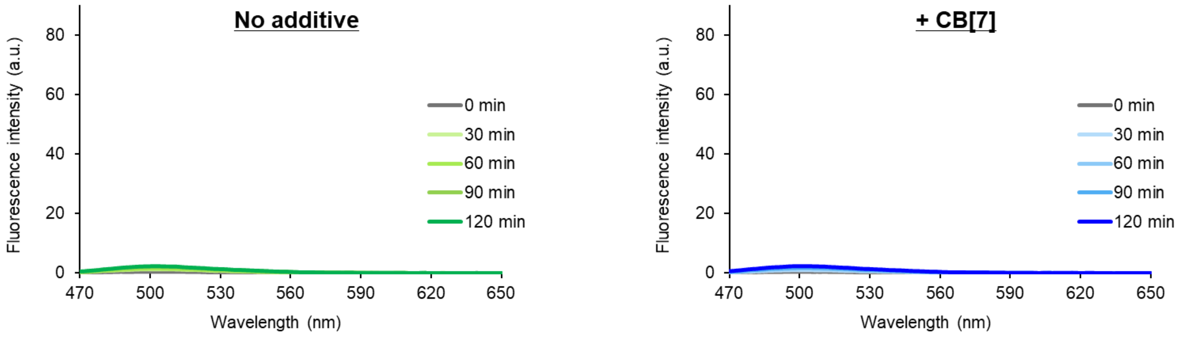

(f) SQ-DNA-5 (**X** = ^{Nad}**dA**)

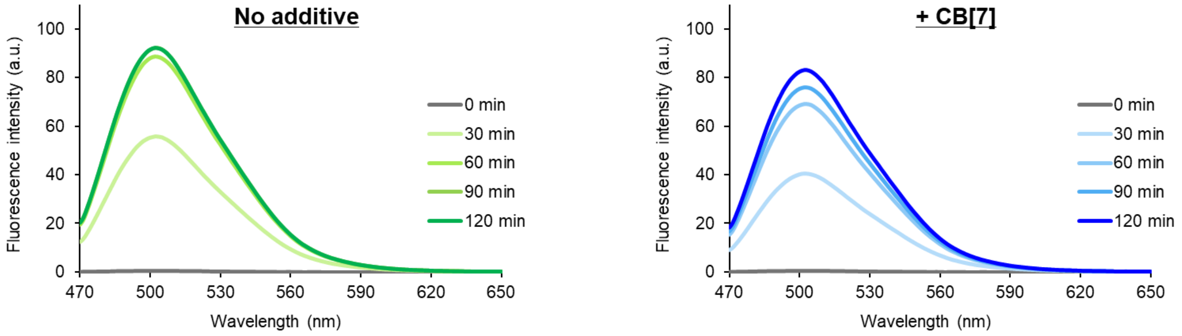

(g) SQ-DNA-6 (**X** = ^{Nad}**dA**)

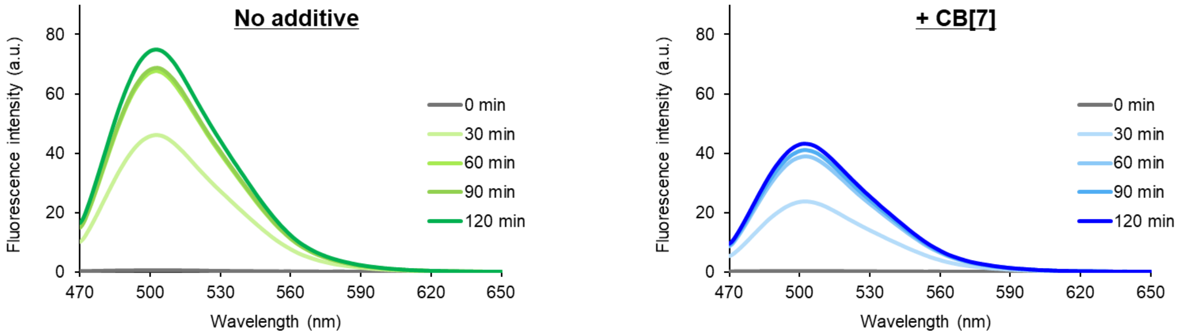

(h) SQ-DNA-7 (**X** = ^{Nad}**dA**)

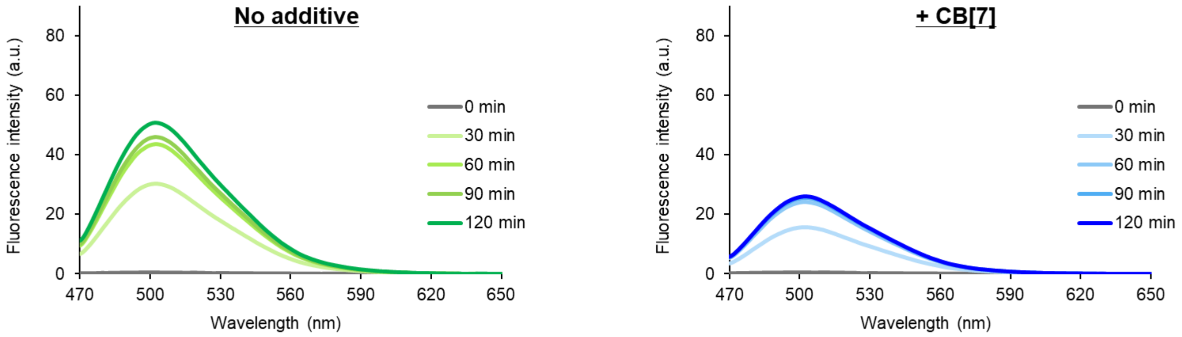

Figure S17 (continued). Fluorescence spectra obtained from the transcription reaction of each **SQ-DNA** in the absence and presence of CB[7]. The spectra were measured using aliquots of the transcription mixture sampled at different time points (0, 30, 60, 90, 120 min) in the presence of DFHBI-1T. (e) **SQ-DNA-4** (**X** = ^{Nad}**dA**). (f) **SQ-DNA-5** (**X** = ^{Nad}**dA**). (g) **SQ-DNA-6** (**X** = ^{Nad}**dA**). (h): **SQ-DNA-7** (**X** = ^{Nad}**dA**).

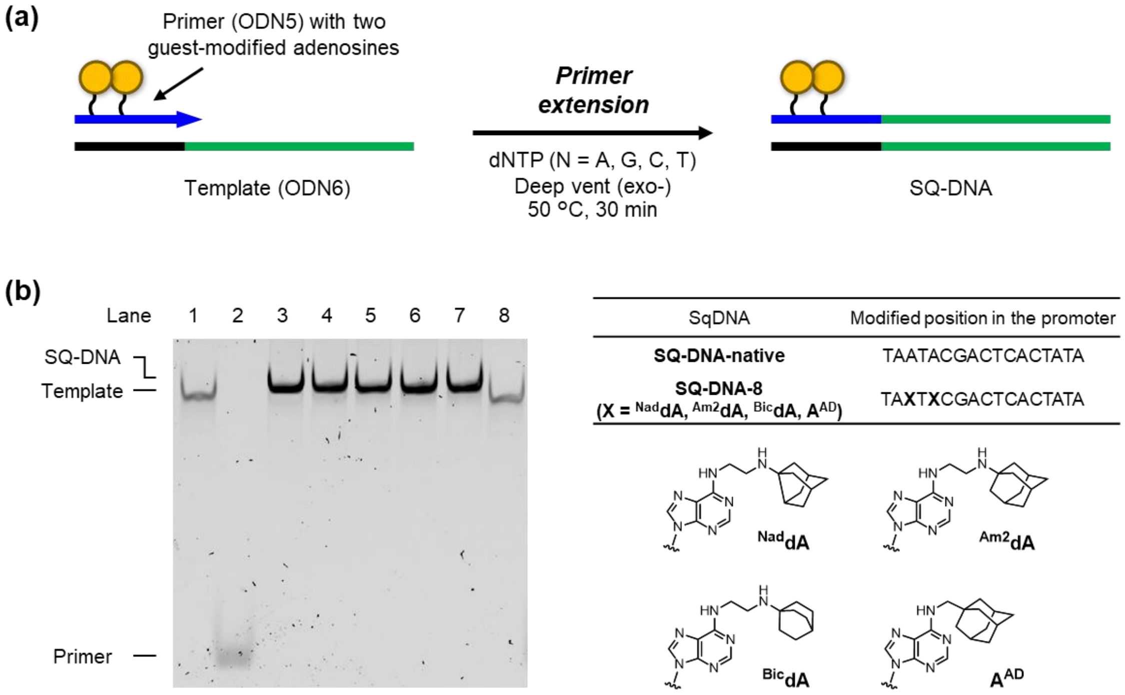

Figure S18. (a) A schematic illustration of the enzymatic synthesis of **SQ-DNA** incorporating two guest-modified adenosines. **ODN5** (primer) was elongated against **ODN6** (template) by Deep Vent (exo-) polymerase in the presence of dNTP. (b) Native PAGE analysis of **SQ-DNA** after spin column purification. Lane 1 and 8: **ODN6** (template). Lane 2: **ODN5-native** (primer). Lane 3: **SQ-DNA-native**. Lane 4: **SQ-DNA-8** (**X** = $^{\mathbf{Am2}}$**dA**). **Lane 5: SQ-DNA-8** (**X** = $^{\mathbf{Nad}}$**dA**). Lane 6: **SQ-DNA-8** (**X** = $^{\mathbf{Bic}}$**dA**). Lane 7: **SQ-DNA-4** (**X** = **A**$^{\mathbf{AD}}$). The DNA bands were stained and visualized using SYBR Gold Nucleic Acid Gel Stain.

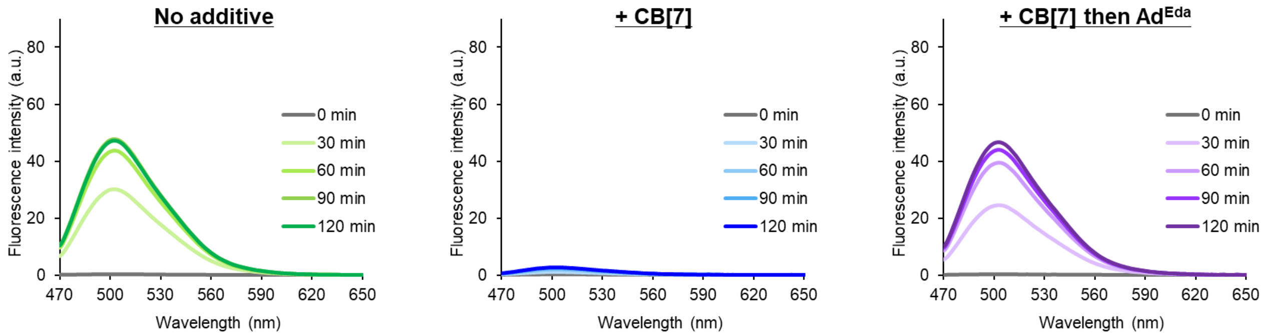

Figure S19. Fluorescence spectra obtained from the transcription reaction of **SQ-DNA-8** (**X** = ^{Nad}**dA**). The reactions were performed in the absence and after alternating addition of CB[7] (8 μM) and Ad^{Eda} (10 μM). The fluorescence was measured using aliquots of the transcription mixture sampled at different time points (0, 30, 60, 90, 120 min) in the presence of DFHBI-1T.

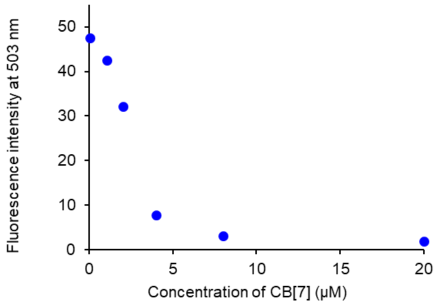

Figure S20. Transcription suppression of **SQ-DNA-8** (**X** = ^{Nad}**dA**) in the presence of different concentration of CB[7] (0, 1, 2, 4, 8, 20 μM). The transcription reactions were performed for 120 min at 37 °C, and the fluorescence at λ_{em} = 503 nm was measured as described above.

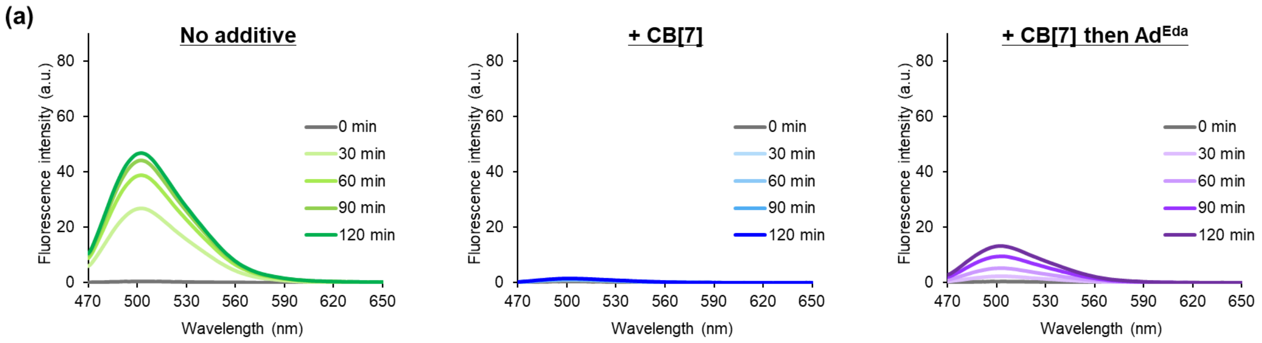

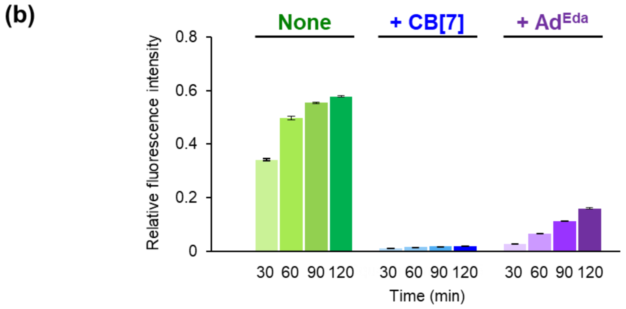

Duplex DNA	Modified position in the promoter
**SQ-DNA-8 (X = ^{Am2}dA)**	TA**X**T**X**CGACTCACTATA

Figure S21. Transcription monitoring of **SQ-DNA-8** (**X** = **^{Am2}dA**). The reactions were performed in the absence and after alternating addition of CB[7] (8 μM) and Ad^{Eda} (10 μM). (a) Fluorescence spectra of each reaction mixture sampled at different time points (0, 30, 60, 90, 120 min). (b) A summary of the relative transcription efficiency.

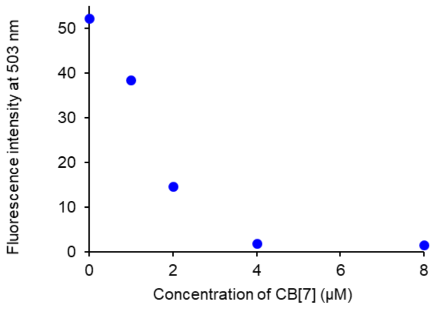

Figure S22. Transcription suppression of **SQ-DNA-8** (**X** = **^{Am2}dA**) in the presence of different concentration of CB[7] (0, 1, 2, 4, 8 μM). The transcription reactions were performed for 120 min at 37 °C. The fluorescence at λ_{em} = 503 nm was measured as described above.

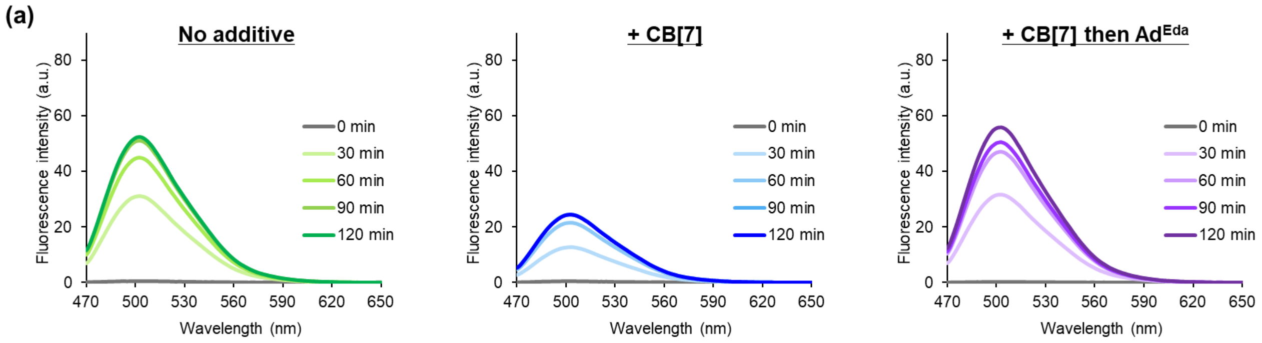

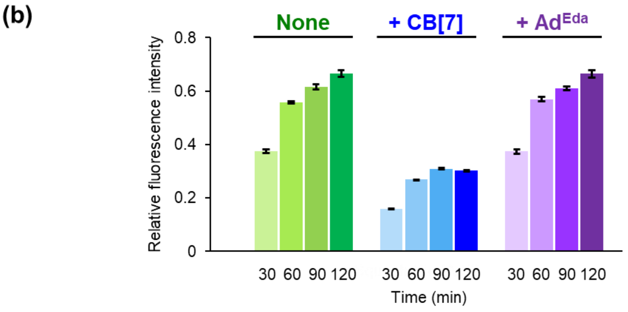

Duplex DNA	Modified position in the promoter
**SQ-DNA-8 (X = ^{Bic}dA)**	TA**X**T**X**CGACTCACTATA

Figure S23. Transcription monitoring of **SQ-DNA-8** (**X** = **^{Bic}dA**). The reactions were performed in the absence and after alternating addition of CB[7] (8 μM) and Ad^{Eda} (10 μM). (a) Fluorescence spectra of each reaction mixture sampled at different time points (0, 30, 60, 90, 120 min). (b) A summary of the relative transcription efficiency.

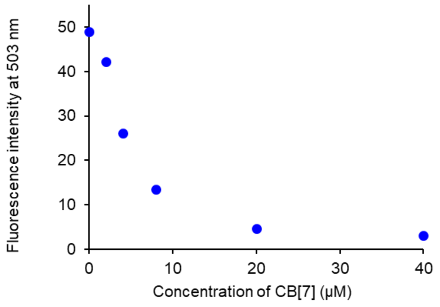

Figure S24. Transcription suppressioin of **SQ-DNA-8** (**X** = **^{Bic}dA**) in the presence of different concentration of CB[7] (0, 2, 4, 8, 20, 40 μM). The transcription reactions were performed for 120 min at 37 °C. The fluorescence at λ_{em} = 503 nm was measured as described above.

(a)

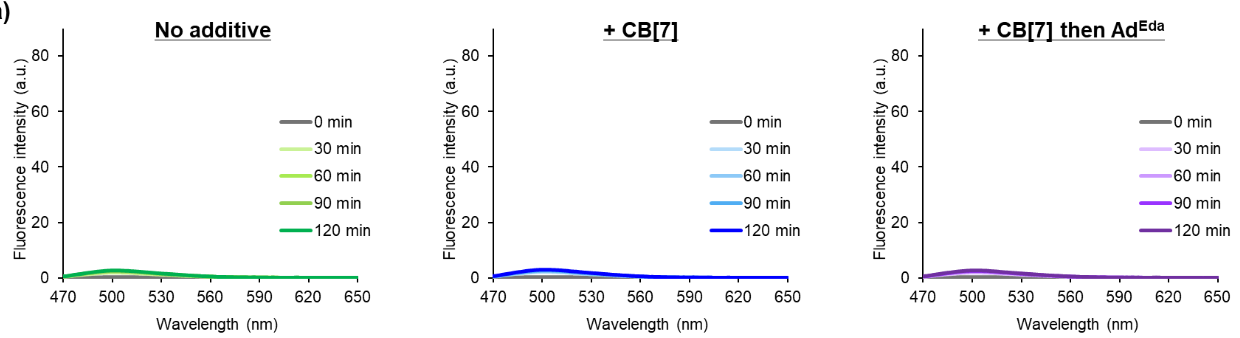

(b)

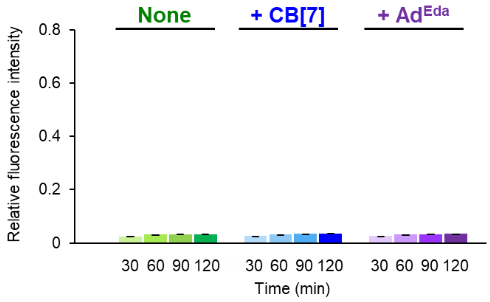

Duplex DNA	Modified position in the promoter
**SQ-DNA-8 (X = A^{AD})**	TA**X**T**X**CGACTCACTATA

Figure S25. Transcription monitoring of **SQ-DNA-8** (**X** = **A^{AD}**). The reactions were performed in the absence and after alternating addition of CB[7] (8 μM) and Ad^{Eda} (10 μM). (a) Fluorescence spectra of each reaction mixture sampled at different time points (0, 30, 60, 90, 120 min). (b) A summary of the relative transcription efficiency. **A^{AD}** induced transcription inhibition regardless of the host-guest interaction.

10. Gene expression control in CFE system using DHFR-DNA

1 gaaatta**X**t**X**cgactcactatagggagaccacaacggtttccctctagaaataattttgt 60

T7 promoter

61 ttaactttaagaaggagatataccaATGATCAGTCTGATTGCGGCGTTAGCGGTAGATCG 120

SD sequence

121 CGTTATCGGCATGGAAAACGCCATGCCGTGGAACCTGCCTGCCGATCTCGCCTGGTTTAA 180

181 ACGCAACACCTTAAATAAACCCGTGATTATGGGCCGCCATACCTGGGAATCAATCGGTCG 240

241 TCCGTTGCCAGGACGCAAAAATATTATCCTCAGCAGTCAACCGGGTACGGACGATCGCGT 300

301 AACGTGGGTGAAGTCGGTGGATGAAGCCATCGCGGCGTGTGGTGACGTACCAGAAATCAT 360

361 GGTGATTGGCGGCGGTCGCGTTTATGAACAGTTCTTGCCAAAAGCGCAAAAACTGTATCT 420

421 GACGCATATCGACGCAGAAGTGGAAGGCGACACCCATTTCCCGGATTACGAGCCGGATGA 480

481 CTGGGAATCGGTATTCAGCGAATTCCACGATGCTGATGCGCAGAACTCTCACAGCTATTG 540

541 CTTTGAGATTCTGGAGCGGCGGtaatgaataactaatcc 579

Figure S26. The nucleotide sequence of DHFR-DNA (dihydrofolate reductase from *E.coli*) with T7 promoter, SD sequence and 3'UTR (579 bps, only the non-template sequence is shown). The ORF encoding DHFR is shown by the capital letter and the others by small letter. **X** in the promoter region refers to **A**, ^{Am2}**dA** or ^{Nad}**dA**. The highlighted (gray) part is the primer binding sites for PCR.

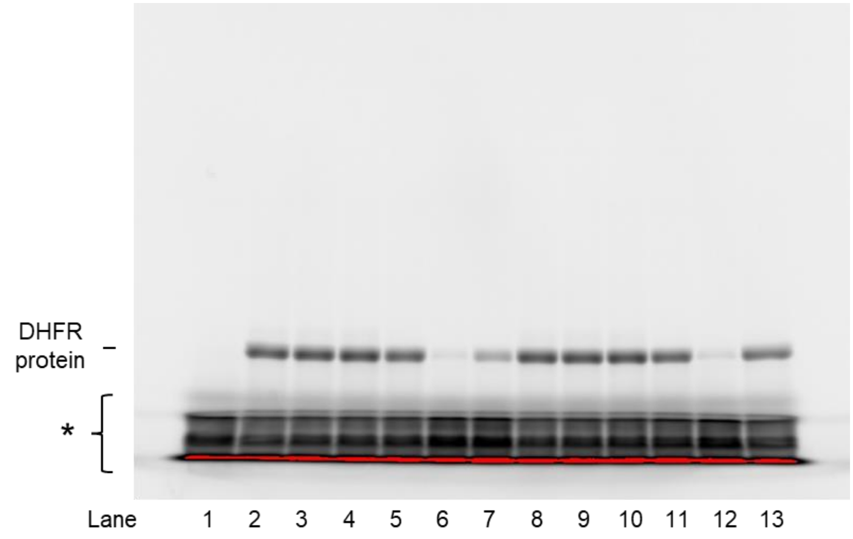

Figure S27. Cell-free expression of **DHFR-DNA** (**X** = **A**, **^{Am2}dA**, **^{Nad}dA**) in the absence and after alternating addition of CB[7] and Ad^{Eda}. The reaction was performed using PUREfrex 1.0 with FluoroTect™ Green$_{Lys}$ in vitro Translation Labeling System and analyzed by SDS-PAGE. The gel image was obtained using ChemiDoc MP Imaging System with an Alexa 488 filter. Lane 1: control without **DHFR-DNA**. Lane 2-4: **DHFR-DNA** (**X** = **dA**) in the absence of CB[7], presence of CB[7] (80 μM) and after the further addition of Ad^{Eda} (100 μM). Lane 5-7: **DHFR-DNA** (**X** = **^{Am2}dA**) in the absence of CB[7], presence of CB[7] (8 μM) and after the further addition of Ad^{Eda} (10 μM), Lane 8-10: same as Lane 2-4. Lane 11-13: **DHFR-DNA** (**X** = **^{Nad}dA**) in the absence of CB[7], presence of CB[7] (80 μM) and after the further addition of Ad^{Eda} (100 μM).

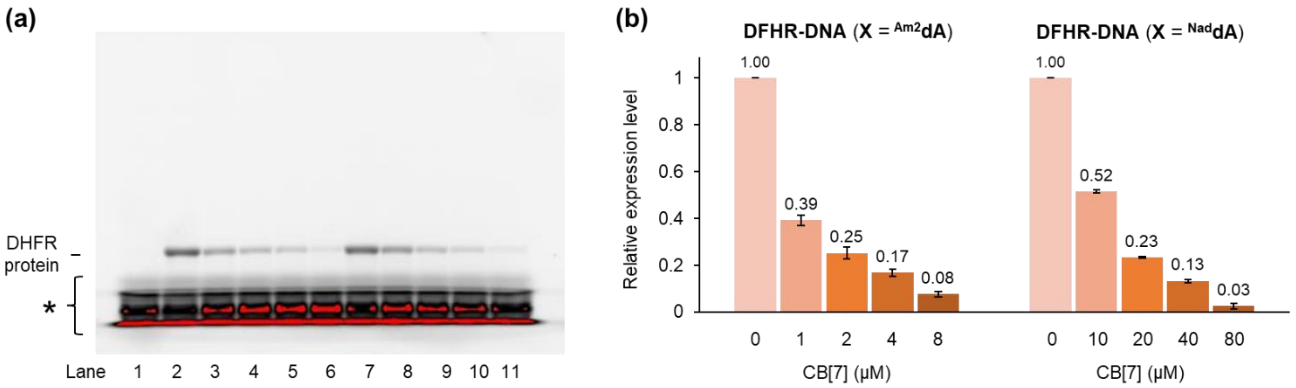

Figure S28. Cell-free expression of **DHFR-DNA** (**X** = **^{Am2}dA**) and **DHFR-DNA** (**X** = **^{Nad}dA**) in the presence of different concentration of CB[7]. (a) A SDS-PAGE analysis of the reaction mixture. Lane 1: control without **DHFR-DNA**. Lane 2-6: **DHFR-DNA** (**X** = **^{Am2}dA**) in the presence of 0, 1, 2, 4, 8 μM of CB[7]. Lane 7-11: **DHFR-DNA** (**X** = **^{Nad}dA**) in the presence of 0, 10, 20, 40, 80 μM of CB[7]. *RNase-digested fragments of FluoroTect^{TM} Green$_{Lys}$ tRNA. (b) Summary of the relative expression level of DHFR proteins from **DHFR-DNA** (**X** = **^{Am2}dA**) and **DHFR-DNA** (**X** = **^{Nad}dA**) in the presence of different concentrations of CB[7].

11. NMR charts of the synthesized compounds

Compoound **S1**

^{1}H NMR (400 MHz, $CDCl_3$)

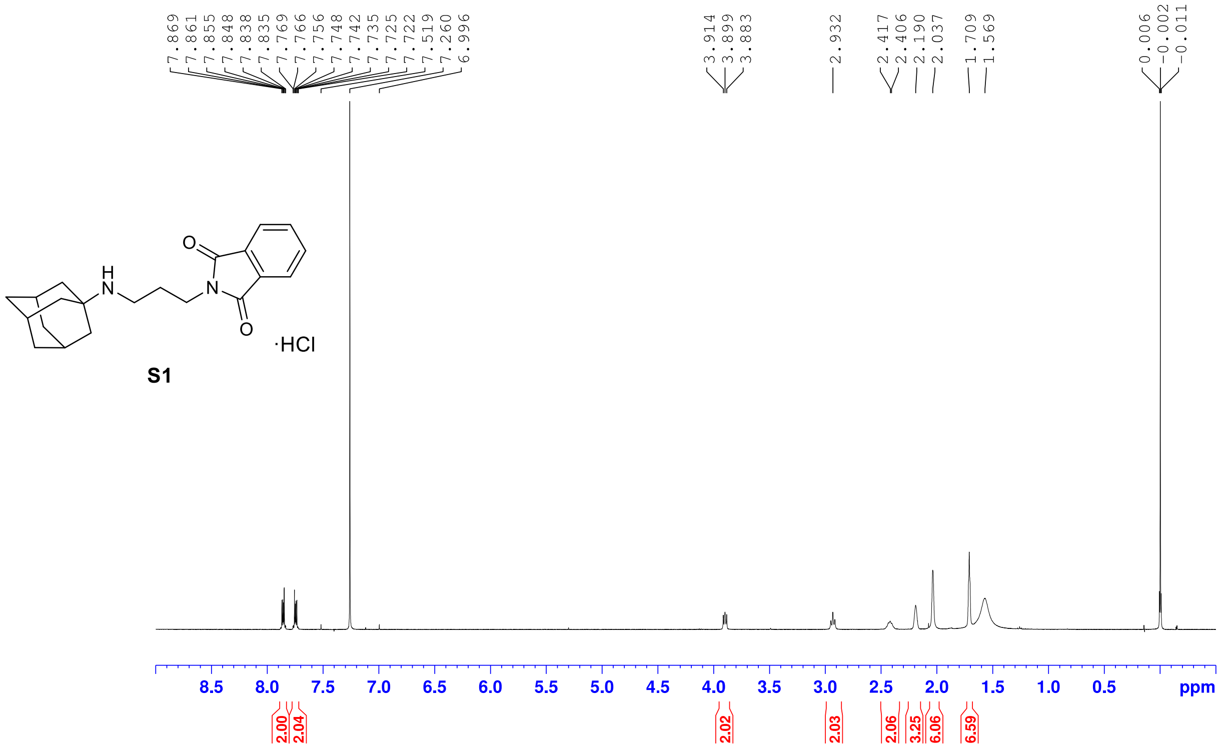

^{13}C NMR (126 MHz, $CDCl_3$)

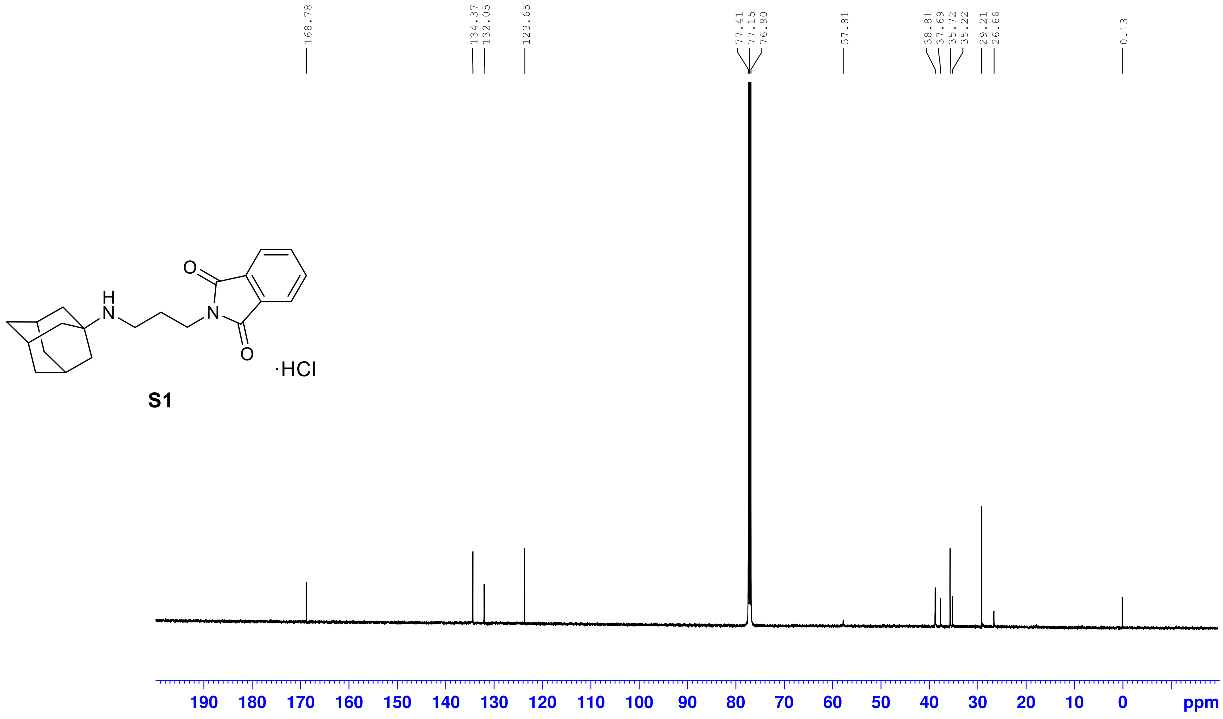

Compoound **3**

^{1}H NMR (400 MHz, DMSO-d_6)

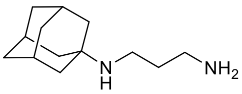

3

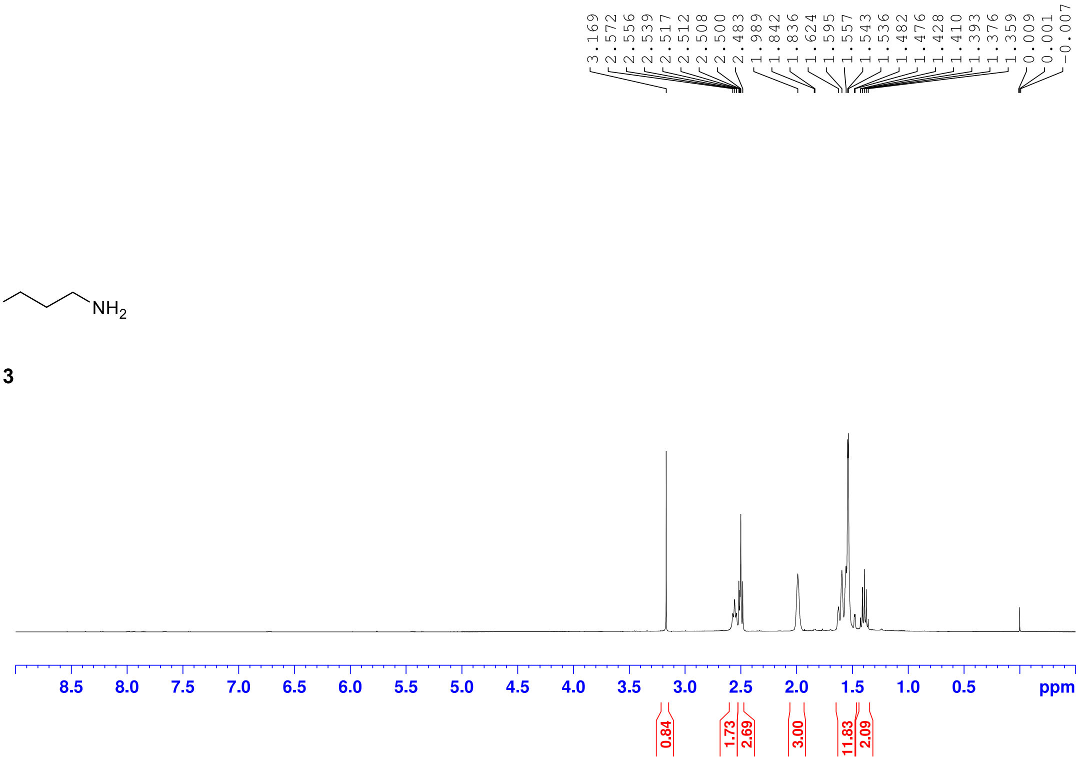

^{13}C NMR (151 MHz, $CDCl_3$)

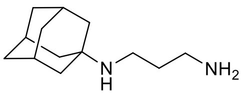

3

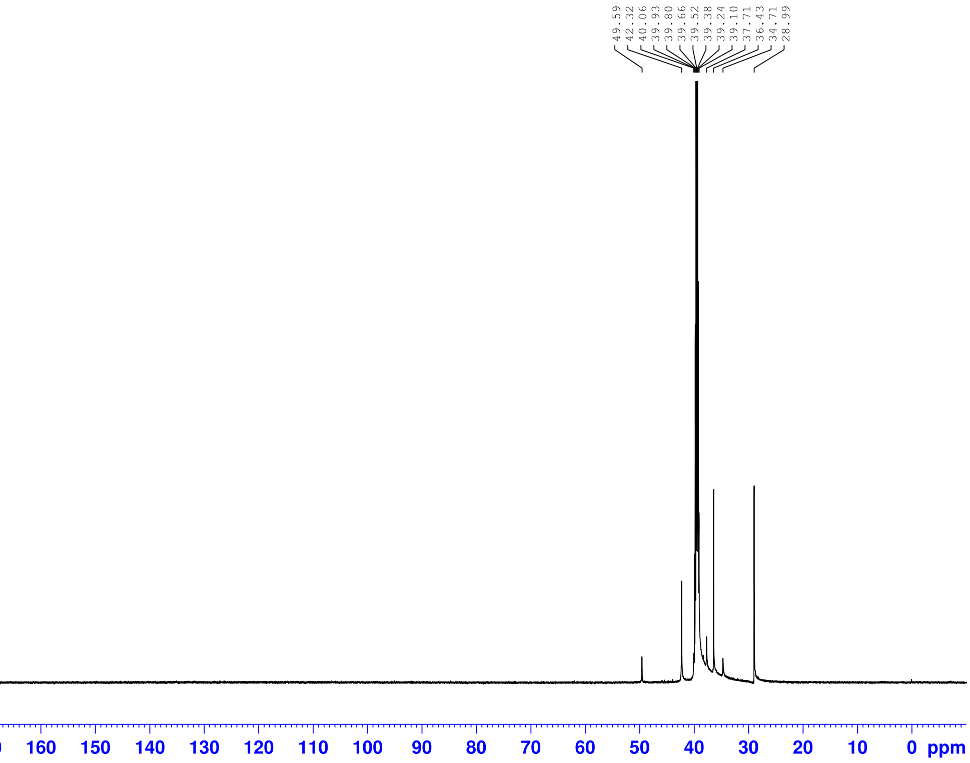

Compoound **S2**

^{1}H NMR (400 MHz, DMSO-d_6)

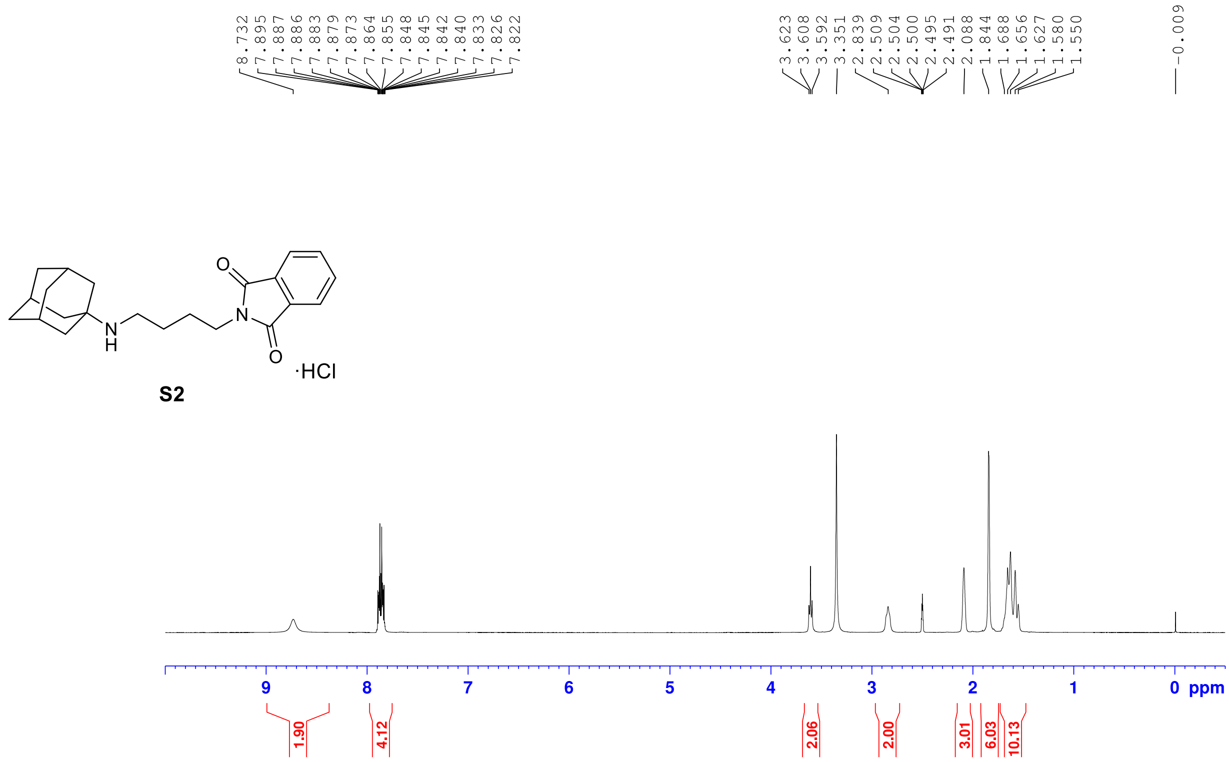

^{13}C NMR (126 MHz, DMSO-d_6)

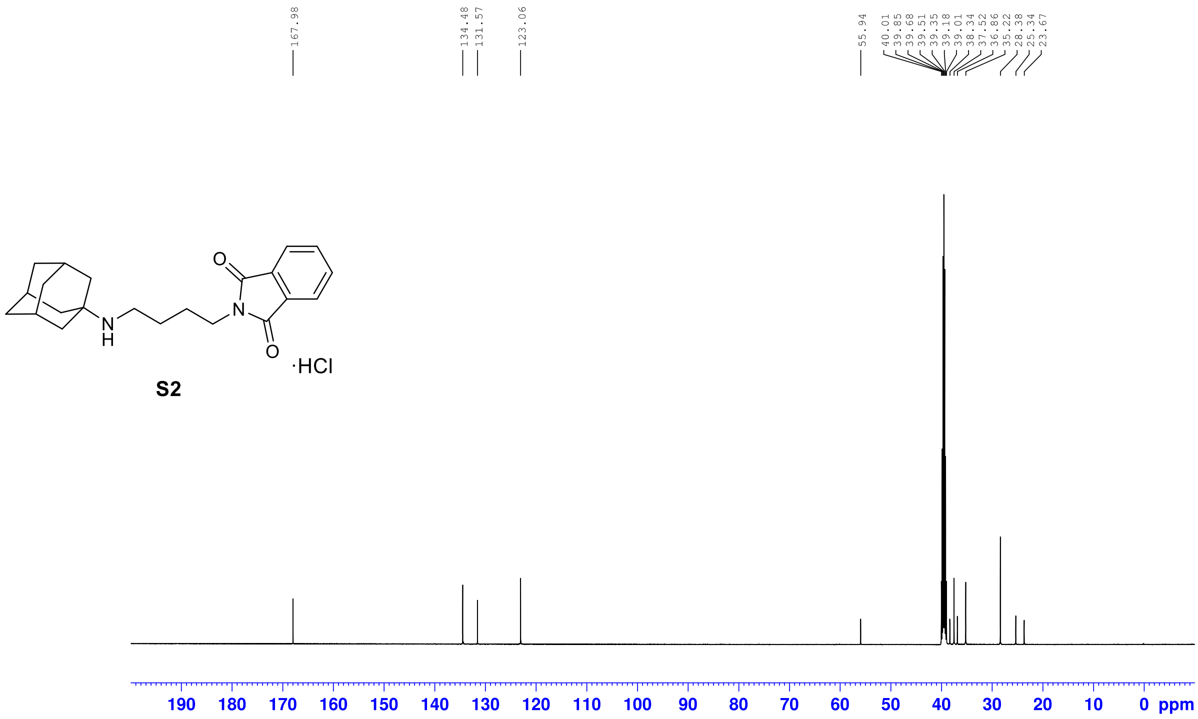

Compooound **4**

^{1}H NMR (600 MHz, DMSO-d_6)

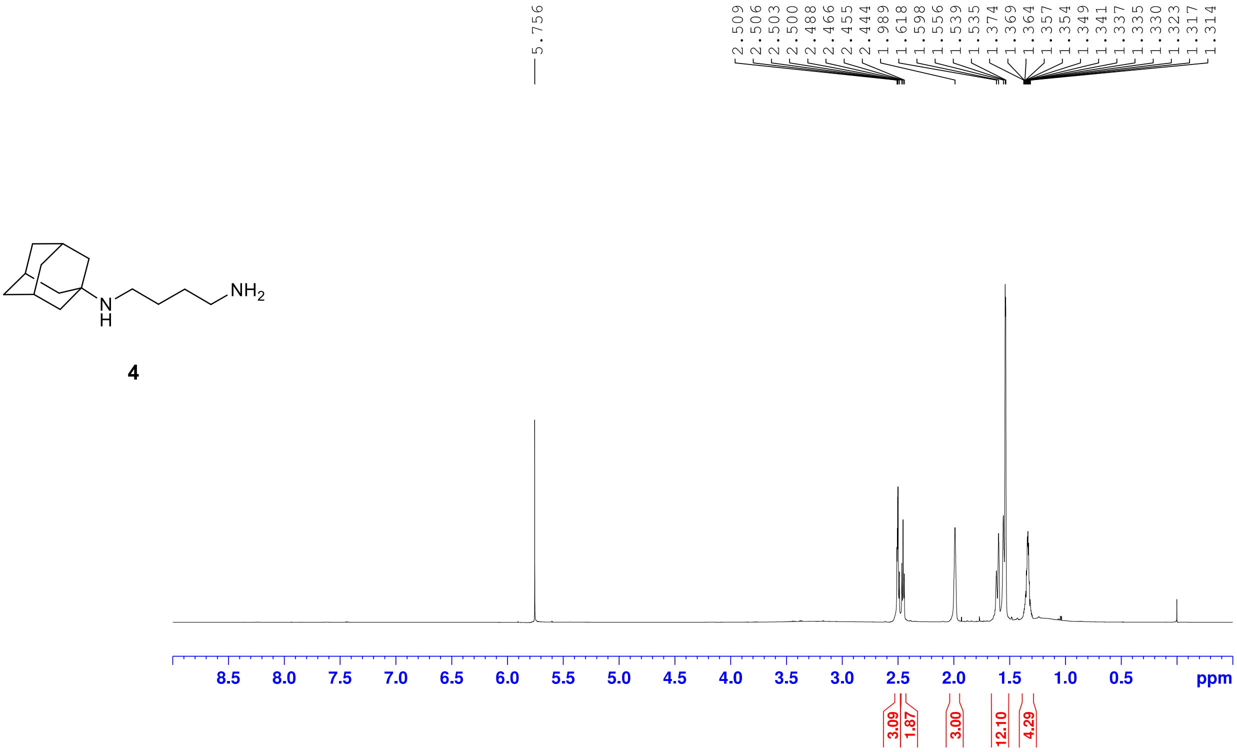

^{13}C NMR (151 MHz, DMSO-d_6)

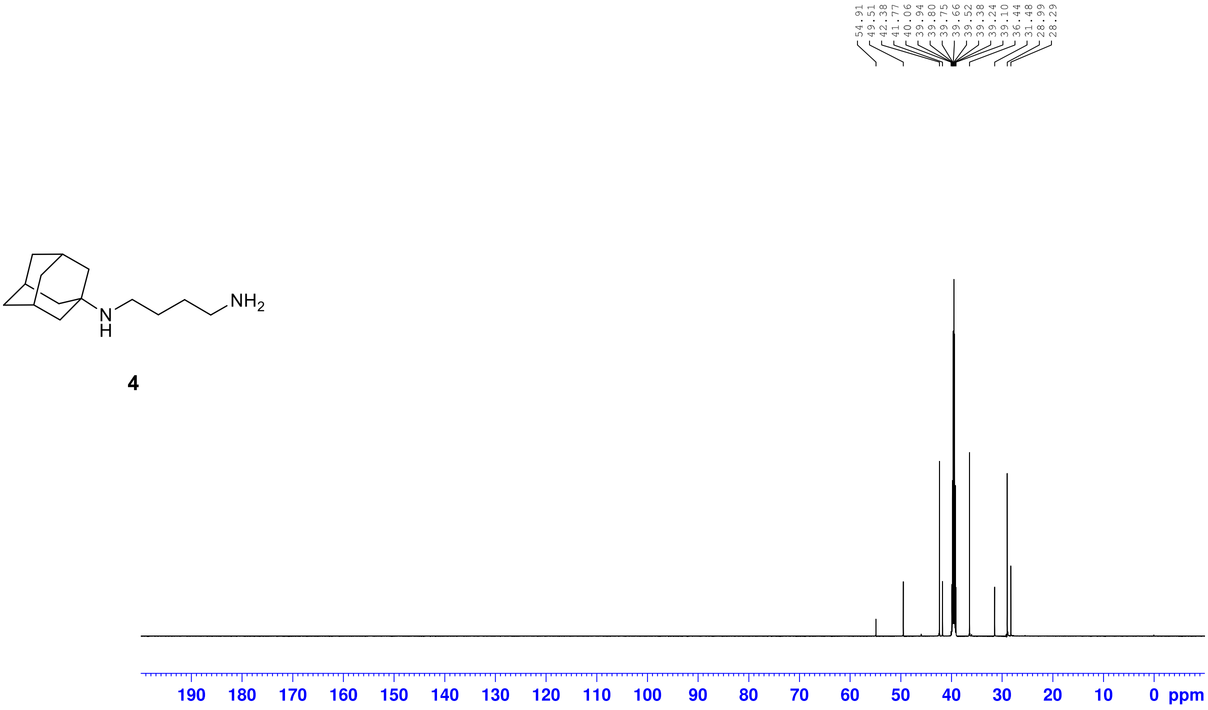

Compooound **S4**

^{1}H NMR (400 MHz, MeOD-d_4)

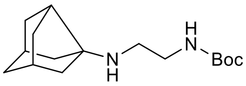

S4

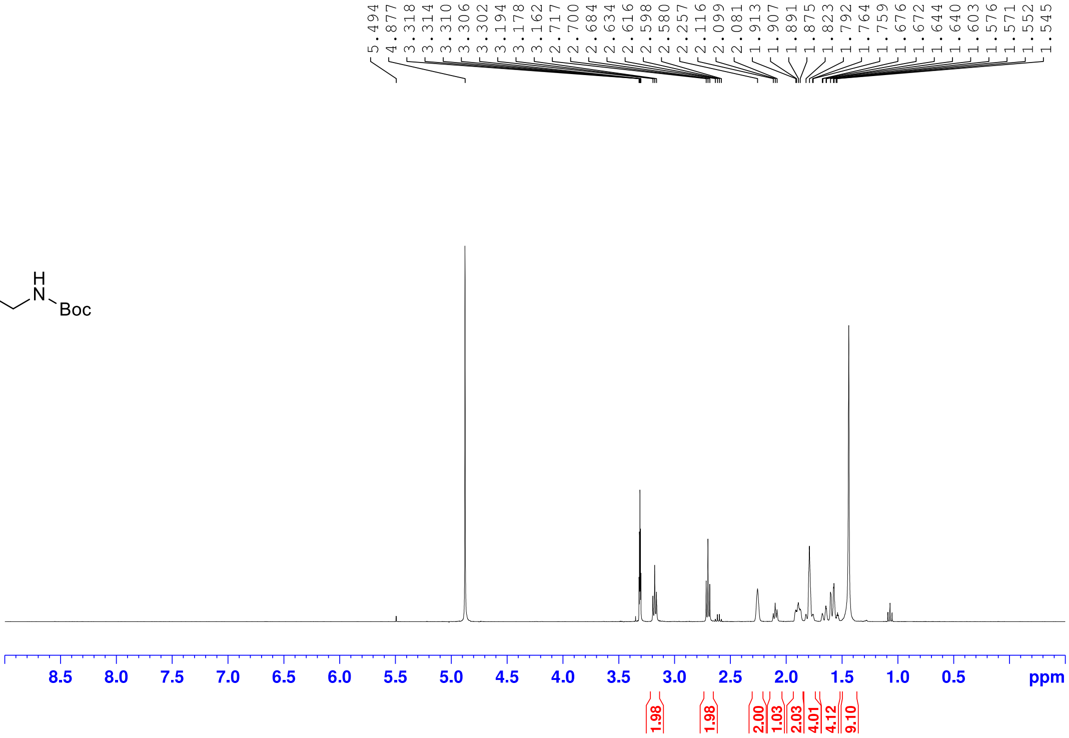

^{13}C NMR (151 MHz, MeOD-d_4)

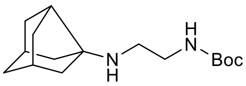

S4

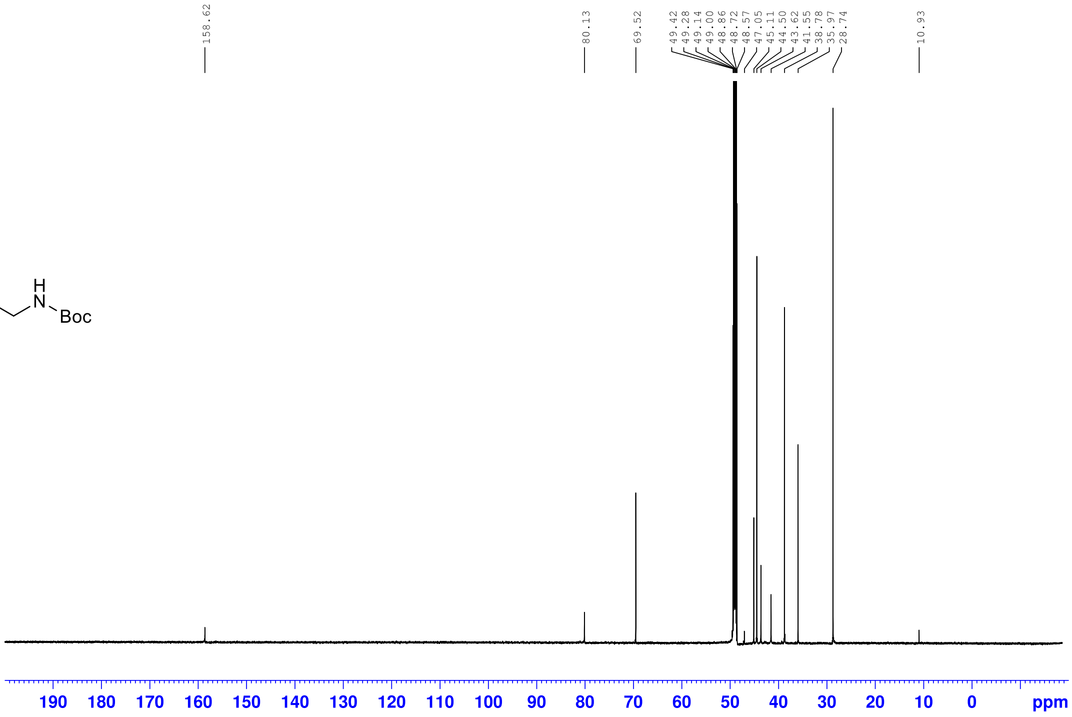

Compoound **5**

^{1}H NMR (400 MHz, MeOD-d_4)

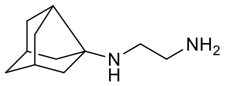

5

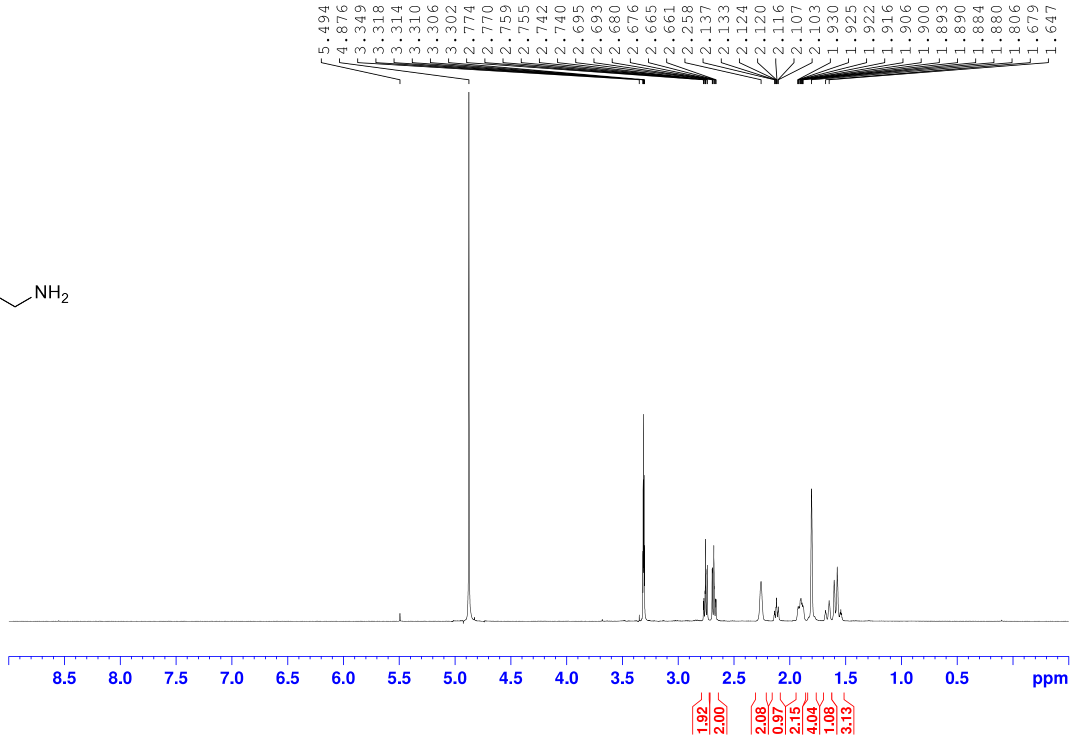

^{13}C NMR (151 MHz, $CDCl_3$)

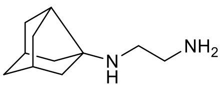

5

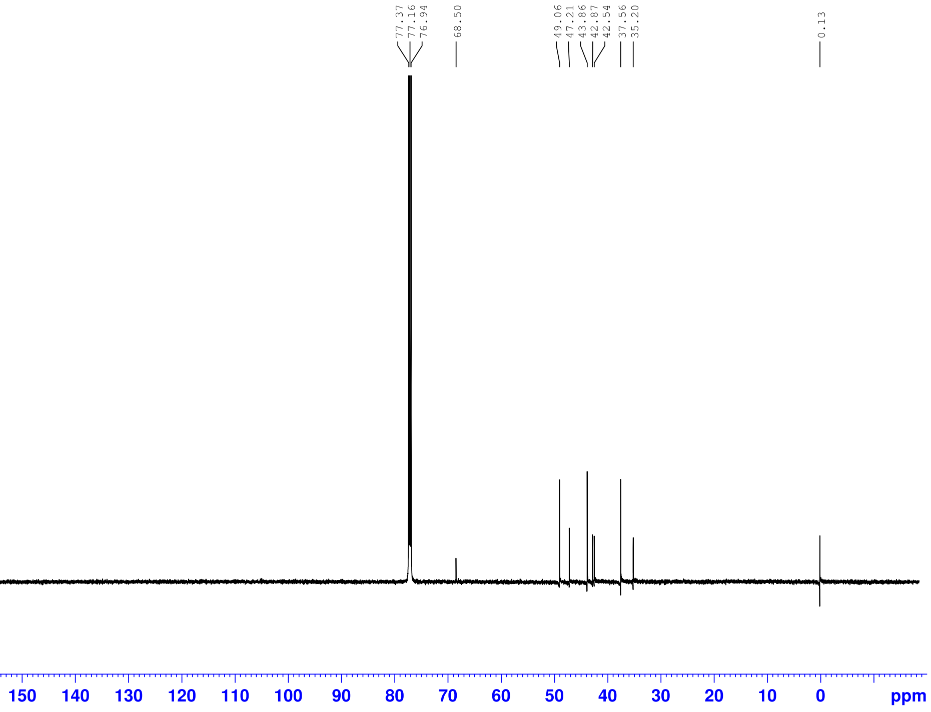

Compoound **S6**

^{1}H NMR (400 MHz, DMSO-d_6)

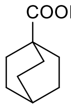

S6

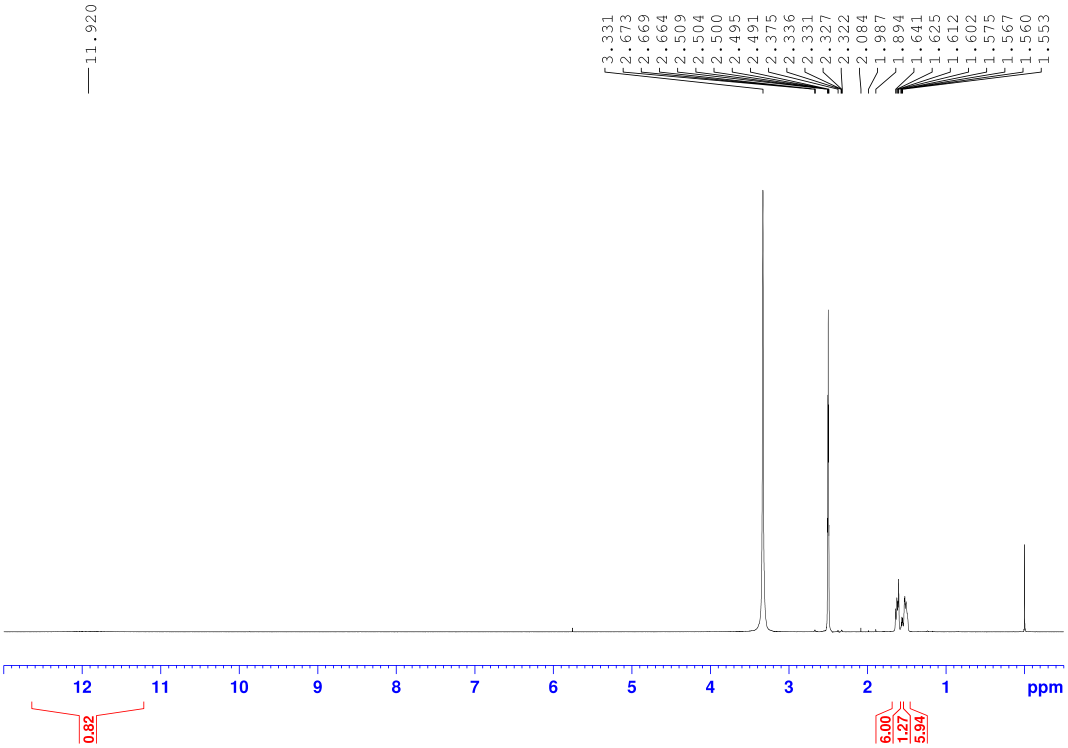

^{13}C NMR (151 MHz, MeOD-d_4)

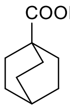

S6

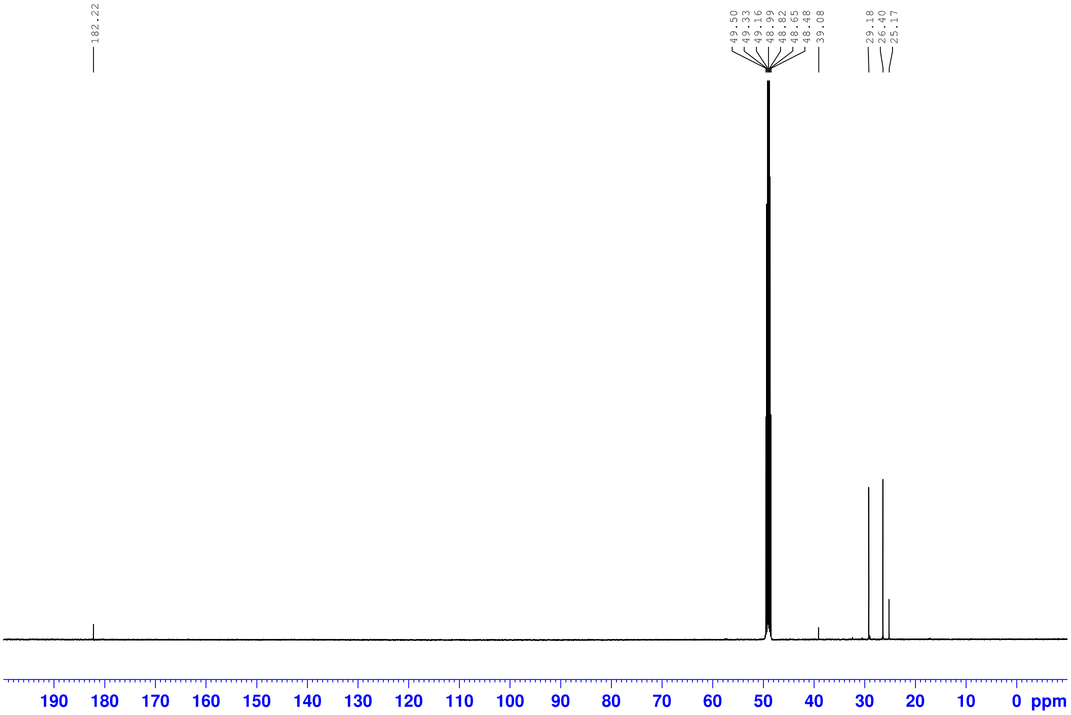

Compoound **S7**

^{1}H NMR (400 MHz, MeOD-d_4)

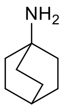

S7

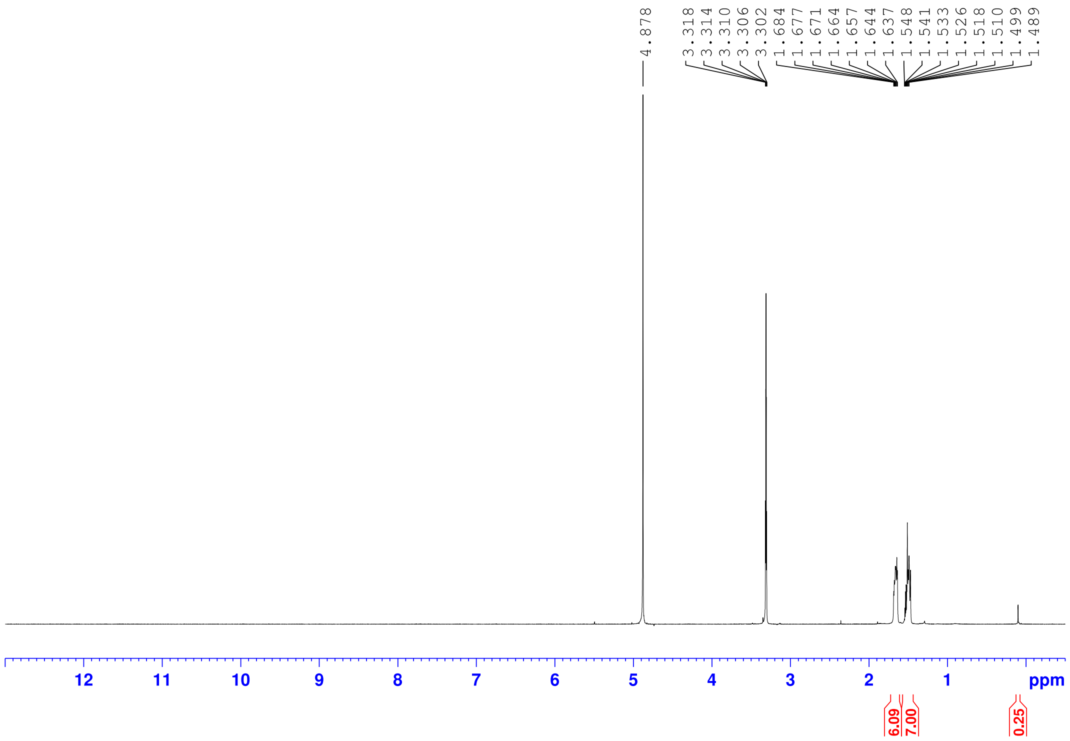

^{13}C NMR (151 MHz, MeOD-d_4)

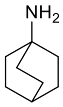

S7

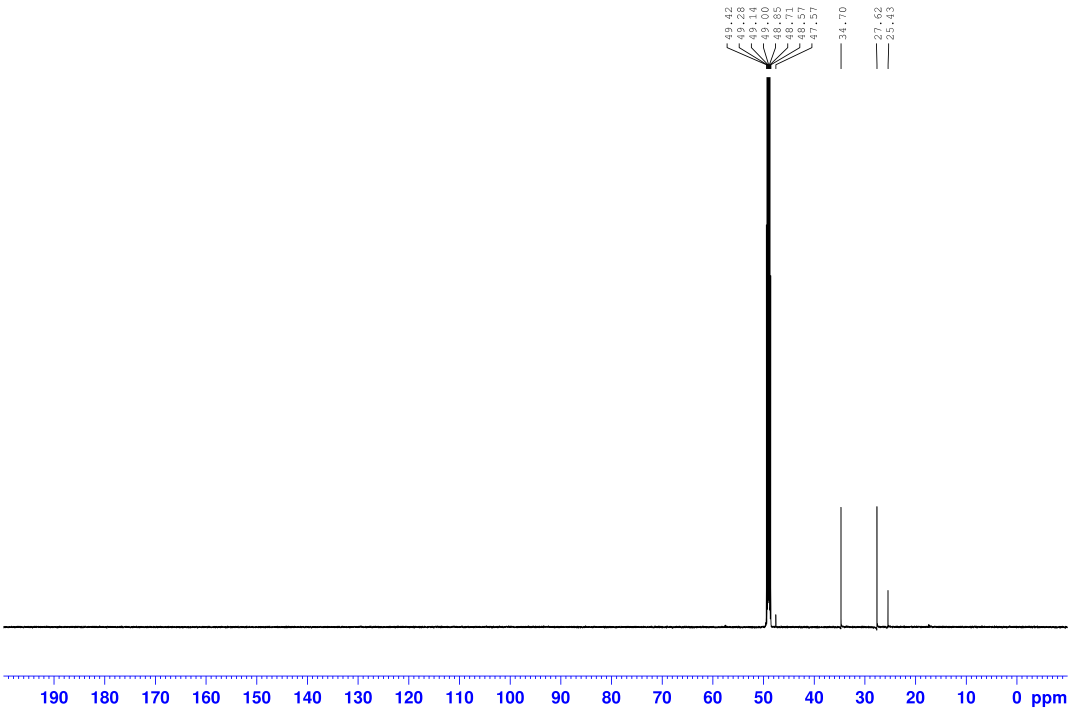

Compoound **S8**

^{1}H NMR (400 MHz, MeOD-d_4)

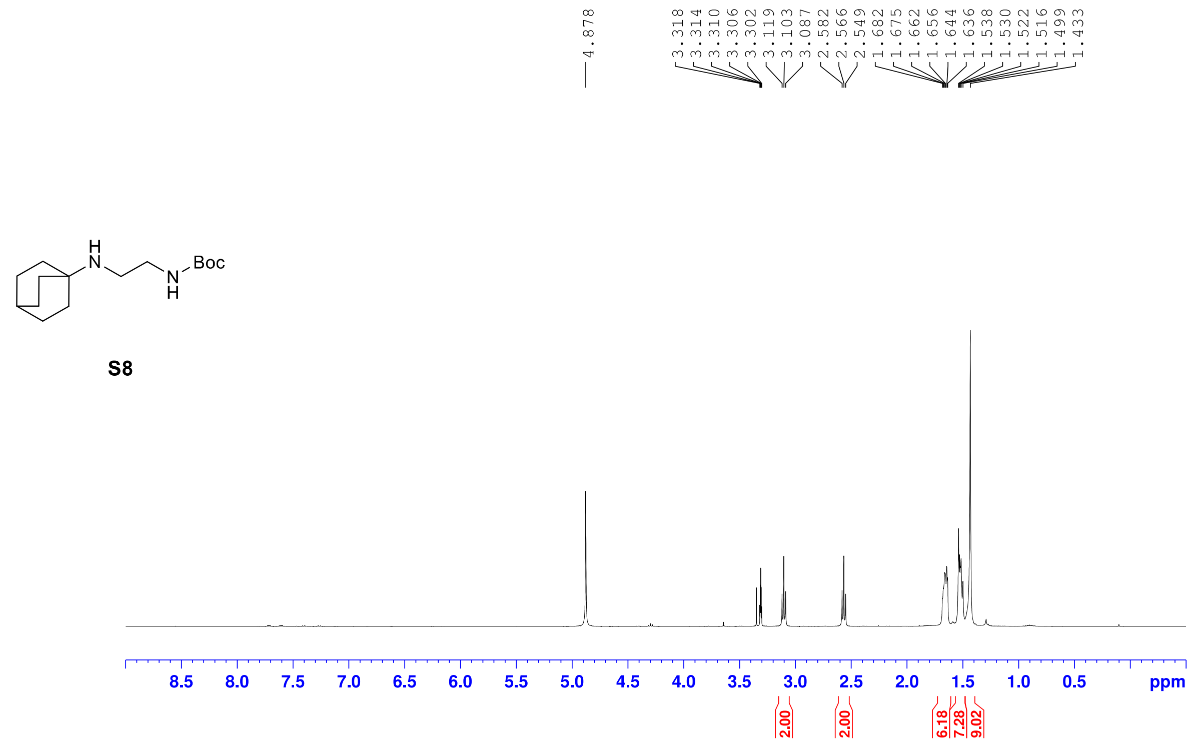

^{13}C NMR (151 MHz, MeOD-d_4)

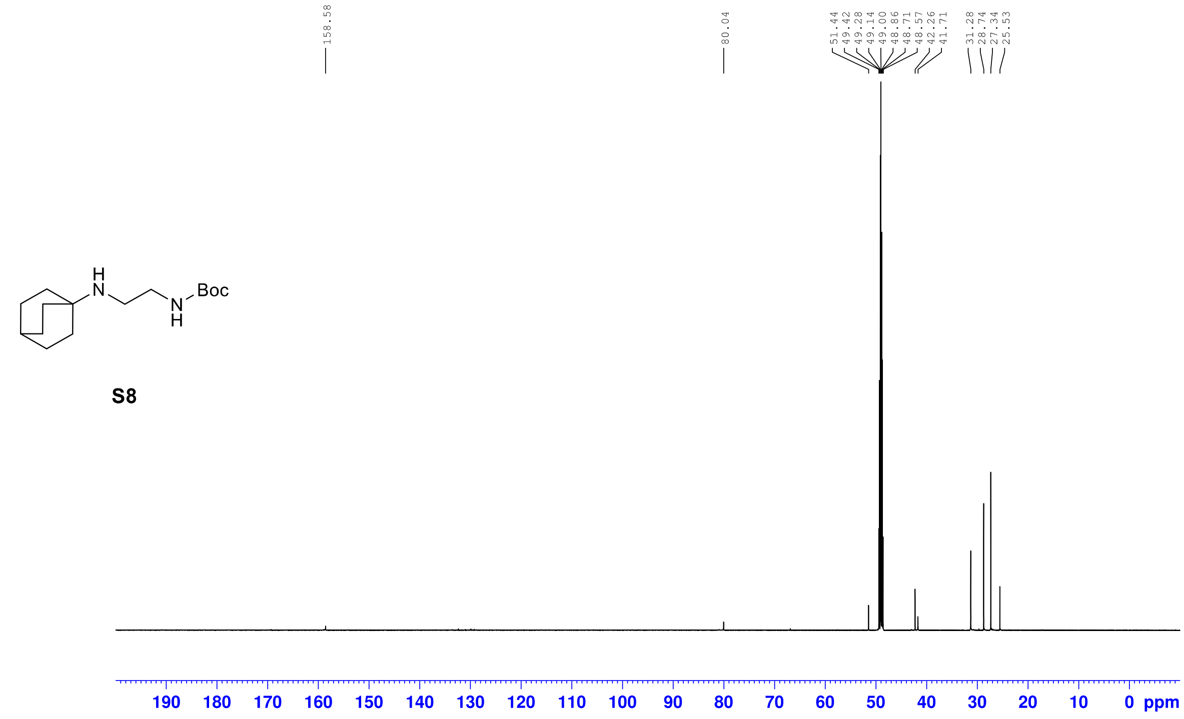

Compoound **6**

^{1}H NMR (400 MHz, MeOD-d_4)

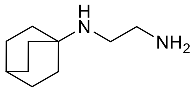

6

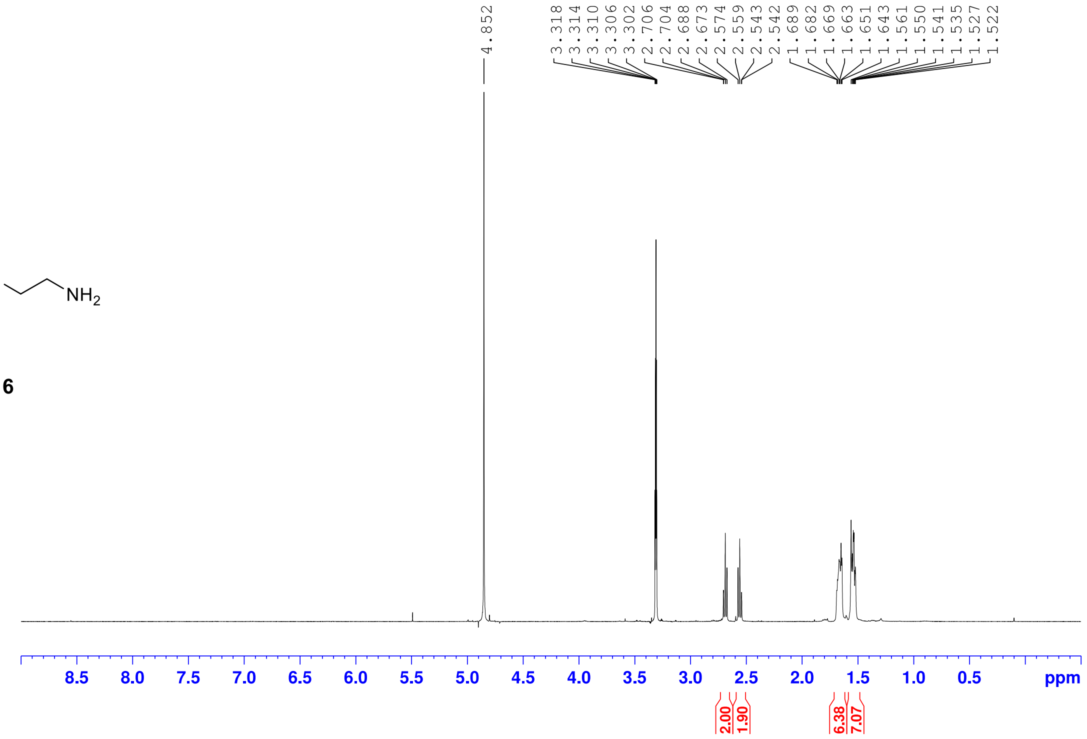

^{13}C NMR (151 MHz, MeOD-d_4)

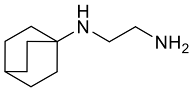

6

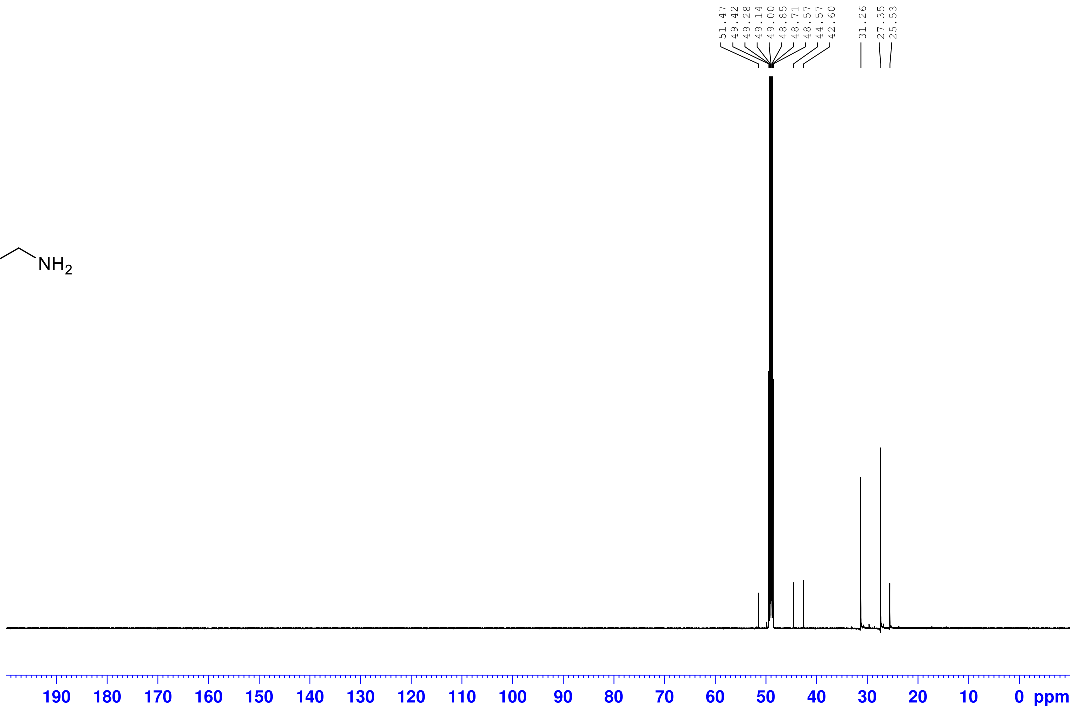

12. References

[1] H. Okamura, G. H. Trinh, Z. Dong, W. Fan, F. Nagatsugi, *Molecules*, **2023**, *28*, 1766.

[2] Y. Shimizu, A. Inoue, Y. Tomari, T. Suzuki, T. Yokogawa, K. Nishikawa, T. Ueda, *Nat. Biotech.*, **2001**, *19*, 751-755.

[3] T. Jiang, S. Bordi, A. E. McMillan, K. Chen, F. Sato, P. L. Nichol, B. M. Wanner, J. W. Bode, *Chem. Sci.*, **2021**, *12*, 6977-6982.

[4] E. B. McLean, D. T. Mooney, D. J. Burns, A. Lee., *Org. Lett.*, **2022**, *24*, 686-691.
